# The Adipokine Component in the Molecular Regulation of Cancer Cell Survival, Proliferation and Metastasis

**DOI:** 10.3389/pore.2021.1609828

**Published:** 2021-09-13

**Authors:** Muhammad Ihtisham Umar, Waseem Hassan, Ghulam Murtaza, Manal Buabeid, Elshaimaa Arafa, Hafiz Muhammad Irfan, Mohd Zaini Asmawi, Xianju Huang

**Affiliations:** ^1^ Department of Pharmacy, COMSATS University Islamabad, Lahore Campus, Lahore, Pakistan; ^2^ Department of Clinical Sciences, Ajman University, Ajman, United Arab Emirates; ^3^ Medical and Bio-allied Health Sciences Research Centre, Ajman University, Ajman, United Arab Emirates; ^4^ Department of Pharmacy, University of Sargodha, Sargodha, Pakistan; ^5^ School of Pharmaceutical Sciences, University of Science Malaysia, Pulau Pinang, Malaysia; ^6^ College of Pharmacy, South-Central University for Nationalities, Wuhan, China

**Keywords:** cell cycle, adipokines, mesenchymal transition, leptin signaling, obesity derived cancers

## Abstract

A hormonal imbalance may disrupt the rigorously monitored cellular microenvironment by hampering the natural homeostatic mechanisms. The most common example of such hormonal glitch could be seen in obesity where the uprise in adipokine levels is in virtue of the expanding bulk of adipose tissue. Such aberrant endocrine signaling disrupts the regulation of cellular fate, rendering the cells to live in a tumor supportive microenvironment. Previously, it was believed that the adipokines support cancer proliferation and metastasis with no direct involvement in neoplastic transformations and tumorigenesis. However, the recent studies have reported discrete mechanisms that establish the direct involvement of adipokine signaling in tumorigenesis. Moreover, the individual adipokine profile of the patients has never been considered in the prognosis and staging of the disease. Hence, the present manuscript has focused on the reported extensive mechanisms that culminate the basis of poor prognosis and diminished survival rate in obese cancer patients.

## Introduction

A sluggish lifestyle is often associated with a disequilibrium between calorie-intake and energy expenditure. Such disproportion accounts for an increase in the bulk of adipose tissue, principally the white adipose tissue (WAT) that eventually precipitates in obesity. WAT serves as a major fat reservoir wherein the upregulation of PPARγ and C/EBPα abet a rapid proliferation and differentiation of precursor cells into unilocular adipocytes [1]. More than 98% mass of these adipocytes is covered by a single lipid droplet, leaving less than 2% space for nucleus and organelles. Committed to store triglycerides in their fat droplets, these cells contain very few mitochondria and remain sedentary. Contrarily, the multilocular adipocytes of the brown adipose tissue (BAT) contain several smaller fat droplets and plenty of mitochondria in their cytoplasm. These adipocytes upregulate uncoupling protein-1 (UCP-1) that instigate heat generation via ‘non-shivering thermogenesis’ [2]. Previously, BAT was believed to exist in neonates exclusively, it is only recently that the anatomical location of BAT is reported in the axillary, perivascular, perirenal, cervical and supraclavicular regions in adults [3]. Under certain conditions such as hypothermia or exercise, some adipocytes in WAT are transformed into a third type of adipocytic lineage that is termed as “beige-like adipocyte”. Congeneric with BAT, the beige-like adipocytes also generate heat, yet they differ from BAT adipocytes in their location, lineage [4] and markers [5]. Aforesaid transformation of WAT into beige lineage is provoked by sympathetic stimulation via upregulation of bone morphogenetic protein 7 (BMP7) [6] and repression of Foxp1 through fibroblast growth factor signaling [7].

The unilocular adipocytes of WAT not only store fats, but they also release hormones that are termed as adipokines. Accordingly, an increasing bulk of WAT in obesity corresponds to a remarkable incline in adipokine levels. In the first place, the consistently higher glucose levels in obesity induce hyperinsulinemia. Secondly, the sensitivity of insulin receptors is dwindled down because of the upsurge in adipokine levels. Such decline in insulin sensitivity not only culminates in type 2 diabetes mellitus, the hyperinsulinemia and insulin resistance directly increase the risk of many cancers such as breast, endometrial, colorectal and pancreatic carcinoma. Consequently, the obese population is considered at higher risk for cancers. This ‘under-rated’ notion requires enormous public attention by virtue of the rapidly increasing obese population that has gone triple since 1975 as per the fact sheets of The World Health Organization [8]. In a cohort study on 1.2 million obese women with high body mass index (age 50–64 years), the occurrence of cancers such as breast, endometrial, colorectal, non-Hodgkin lymphoma, leukemia, ovarian and pancreatic carcinoma was found as much higher as 58.8% [9]. According to the United States population data of 2012, around 3.5% (28,000) of all annually reported cancers in men were associated with obesity [10]. The women were found even more vulnerable with around 9% (72,000) obesity derived cancers reported per year. In a parallel study, around 21% of all cancer cases reported in Nigeria between 2012 and 2014 were linked with obesity [11]. Although of relatively lower magnitude, the risk of pancreatic cancer in obese population was significantly higher than the non-obese subjects [12].

**FIGURE 1:**
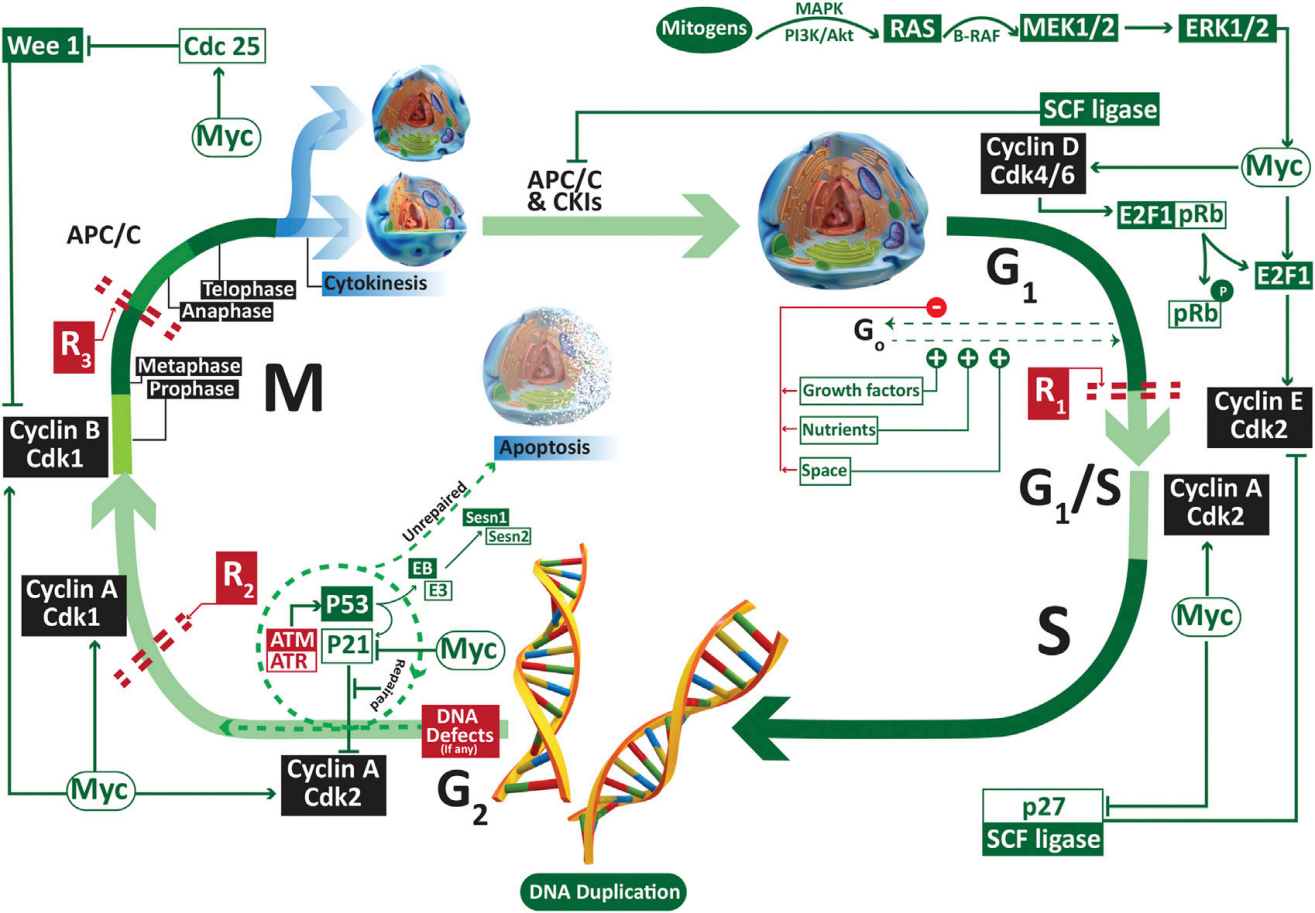
The regulators of G1, S, G2 and M phase of cell cycle. A normal cell remains dependent on external factors such as the nutrients, growth factors and growing space only in the early G1 phase. A lack of even one of these three factors may result into the reversible escape (G_o_ or quiescence) of the cell from cell cycle. Once the cell crosses the first restriction phase R1, it becomes independent of the availability of the external factors. Abbreviations: APC/C anaphase promoting complex/cyclosome; ATM ataxia telangiectasia mutated; ATR ATM-and Rad3 related; Cdk cyclin dependent kinase; R restriction point.

The regulation of cell cycle and its check points are presented in Figure 1. Under normal conditions, the cells remain in a reversible stationary phase (quiescence) until they are provided with optimum growing space, nutrients and growth factors [13]. Quiescence is associated with an upregulation of serum deprived early response genes (SDERGs) including tumor repressor gene SALL2 and MXI1 [14]. Once that the required factors are provided, the cells upregulate proliferative genes such as c-Myc and cylins that promote the entry of these previously quiescent cell to the cell cycle. The basic difference between the normal and cancer cells lies in the fact that the later become independent of either of the above-mentioned factors. Such lack of sensitivity to the mitogen availability and space makes the cancer cells grow beyond the limits, provoking drastic physiological modifications by inducing aberrant angiogenesis to support their freaky growth. Hence, the first deviation of the cancer cells from normal lineage is their escape from quiescence in mitogen deprived conditions. Despite of the detailed studies on the expression of different proliferative genes involved in the escape of cells from quiescence, the role of adipokine upswing in the dysregulation of quiescence check points necessitates immense consideration.

**FIGURE 2:**
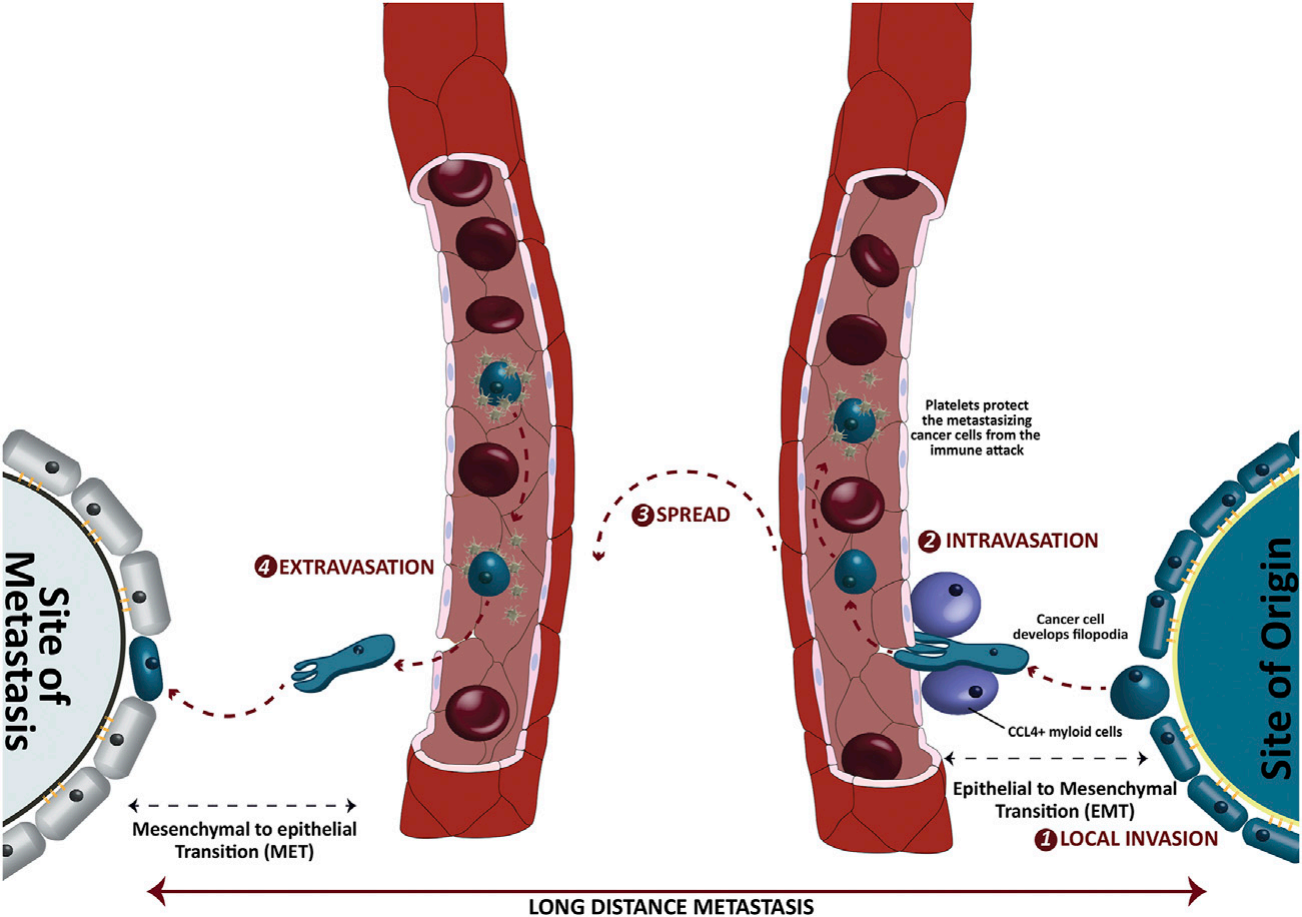
Different stages of long-distance metastasis of epithelial cancer cells. The cancer cells invade the surrounding tissues and intravasate a nearby blood capillary via chemokine ligand 4 positive (CCL4+) myeloid cells. Once entered in a capillary, the platelets protect these spreading cancer cells from a possible immune attack. The epithelial to mesenchymal transition (EMT) helps these cancer cells to downregulate the epithelial markers such as E-cadherins and develop filopodia. On the other side, the mesenchymal to epithelial transition (MET) upregulates the epithelial characters to get localized at the distant new location.

In addition to the dysregulation of cell cycle regulation, the metastasis is another major impediment in the management of cancers. As an estimate, more than 90% of cancer caused deaths involve the spread of cancer cells from their site of origin to the distant locations [15]. The tendency to spread depends on many factors, one of which is the type of the disease itself. For instance, brain tumors and skin carcinomas seldom metastasize [16], albeit their high tendency to invade locally. On the other side, bone sarcomas metastasize quickly to other tissue, especially the lungs [17]. The different stages of metastasis are graphically presented in Figure 2. In general, the process of metastasis can be divided into four major stages, the local invasion, intravasation, spread and extravasation where local invasion is the primary step that paves way to the distant colonization of the cancer cells. Despite of being fully differentiated, the epithelial cancer cells have to endure a morphological shift from epithelial to mesenchymal attributes to invade the surrounding tissue through a process that is termed as the epithelial to mesenchymal transition (EMT). Although such EMT is a much-needed physiological phenomena in embryogenesis and wound healing, it sets the stage for the more detrimental distant translocation of cancer cells through blood or lymph vessels via intravasation. Once metastasized, these cells may undergo a reverse mesenchymal to epithelial transition (MET) that facilitates the successful colonization of these cells into a new distant site through extravasation. Since the role of some of the potential adipokines in instigating EMT and cell cycle dysregulation is established, we need to link all possible points on paper to fully understand the role of adipokine upregulation in the pathological quintessence of tumor microenvironment.

It is recently reported that the increasing bulk of fat in obesity is not the independent contributor to cancer pathogenesis. A cohort study on 319,397 Korean subjects has suggested that the people with obesity derived metabolic derangements are at relatively higher risk of colorectal cancer [18]. Despite of the high body mass index, the risk of colorectal cancers and cardiovascular ailments was remarkably minimum in a sub-population of obese subjects who did not show metabolic dysregulations such as high blood pressure, hyperinsulinemia, dyslipidemia, and insulin resistance. Such findings suggest a possible link between the metabolic dysregulation and the neoplastic shift among different tissues particularly the colorectal cells in both male and female subjects. It seems as if the the higher levels of adipokines, pro-inflammatory cytokines and hyperinsulinemia together shift the cellular microenvironment to an immune-suppressant tumor supportive environment that favors the survival, proliferation, and metastasis of cancer cells. Currently, the prognosis and staging of the cancer patients is made according to the tumor node metastasis (TNM) classification that is advised by the Union of International Cancer Control (UICC) [19]. The TNM classification provides current guidelines to classify the metastatic extent, however many inclusions have been suggested over the years to further improve disease prognosis. These suggested inclusions comprise of the genetic factors [20], deep proteomics [21], immunohistochemistry [22] and the molecular signatures [23]. Despite of the established role of obesity in cancer pathogenesis, the consideration of adipokine profile has never been suggested in the staging and profiling of the disease. In the next sections of the manuscript, we now focus on the different molecular mechanisms reported so far to establish the pathological basis of adipokines and obesity mediators in cancer.

### Obesity-Derived Mediators and Cancer

The adipokines released from the adipocytes help these cells to communicate with surrounding tissues to accomplish the regulation of glucose and fat metabolism. Some of these adipokines are exclusively released from adipose such as leptin, resistin, visfatin and omentin whereas the others are secreted from other tissues as well. For instance adiponectin is released from placenta, myocytes, liver parenchymal cells and the osteoblasts. Likewise, tumor necrosis factor (TNF) and interleukin 6 (IL-6) are released from adipocytes and the infiltrating macrophages. Under normal physiological conditions, these adipokines perform valuable functions to maintain homeostasis. However, despite of their potential biological significance, there is a compelling scientific evidence that supports the involvement of these adipokines in the growth and spread of cancer cells. For instance, leptin is not expressed in the normal breast tissue. However, a growing ductal carcinoma induces a significant expression of leptin as a major proliferative factor in the surrounding normal breast tissue, suggesting a possible role of leptin in disease progression [24]. The higher expression levels of resistin and visfatin are reported in a number of malignancies such as colorectal [25], lung [26], gastroesophageal [27], endometrial [28] and breast cancer [29].

Apart from the above-mentioned mediators, there are other mediators whose serum level is often associated with obesity. These include angiopoietin (Ang) and angiopoieting like proteins (ANGLP), chemerins, endotropin, fibroblast growth factor 2, lipocalin 2, neuregulin 4, retinol binding protein 4, autotaxin and its derivative lysophosphatidic acid, bone morphogenic proteins, sphingolipids, fatty acid esters of hydroxyl fatty acids, uric acid and uridine [30]. The over-expression of these mediators is reported in several different malignancies (Please see Supplementary Material Table 1). Interestingly, some of these mediators are found to be specific for a particular type of cancer. For instance, leptin over-expression is found to be relatively more associated with breast malignancies, although its expression is also reported in other cancers as well. Likewise, the over activity of lysophosphatidic acid is highly associated with ovarian cancers. It has been reported earlier that the expression of Ang 1 is negatively associated with the adipose tissue weight. Contrarily, Ang 2 expression is increased in the adipose tissue where it modulates adipose tissue vasculature [31]. Likewise, ANGLP 2 (along with other ANGLPs) is a pro-inflammatory cytokine that is released from the adipose tissue to activate macrophages. The tumor angiogenesis in the tumor supportive microenvironment is attributed to an over-expression of Ang 1, 2, 3 and 4 as well as many ANGLPs in different cancer types.

Owing to the complexity of cancer pathogenesis, several different theories have been postulated in past few years to relate these mediators with cancer initiation and progression. However, the certainty regarding the extent of involvement of these suggested disease mechanisms may incite another discussion that does not come under the scope of this article. For instance, a relatively higher prevalence of malignancies such as the breast, colon, pancreas, and prostate in obese population is already established. Nonetheless, all obese people do not suffer from these cancers in their entire lifespan despite of the fact that their cells are exposed to a microenvironment rich in obesity mediators. We hereby describe the suggested involvement of these mediators with malignancies in a concise manner.

### Leptin

Leptin is a peptide adipokine that plays a significant physiological role in controlling the total mass of adipose tissue to maintain homeostasis. Its physiological significance can be judged by the fact that the genetic silencing of leptin or its receptor have resulted into serious pathological defects such as metabolic derangements, infertility, immune deficiencies and diabetes in knockout mice [32]. Moreover, it affects several cell populations under physiological conditions, including the immune cells, neurons, pancreatic β cells, adipocytes and endothelial cells that suggests a broader physiological role of leptin compared with other adipokines. Once released from the adipocytes, leptin elicits its hunger-suppressing effects by inhibiting neuropeptide Y and anandamine via activating its receptors in the lateral hypothalamus. Under the normal physiological conditions, it regulates proliferation [33], invasion [34] and the absorptive properties [35] of colon cells. Owing to its higher serum concentration in obesity, leptin is one of the most studied adipokines in the progression and metastasis of breast cancer. Higher leptin levels are frequently reported in breast, colon and pancreatic cancers [36,37]. Leptin levels are also correlated with different breast cancer types. For instance, its levels were found higher in patients with ductal carcinoma *in situ* as compared to the invasive breast cancer patients where adiponectin was significantly higher than leptin [38]. Although a previous study claimed either no or even a negative correlation between the serum leptin levels and pre-menopausal breast cancer incidence [39,40], the interrelationship between leptin serum levels and breast cancer risk in post-menopausal women is well-established. In addition to the adipose, leptin is also synthesized and released in significantly higher concentrations from glioblastoma cells that majorly accounts for the morbidities associated with glioblastoma [41]. It elicits its biological function through the leptin receptor B (ObRB) via activating PI3K, JAK2/STAT3 and ERK1/2 signaling. The recent evidence not only supports the possible role of leptin in the initiation of cancer, but it has also been confirmed to promote the invasion and metastasis of cancer cells by activating EMT, inhibiting immune checkpoints and bolstering angiogenesis.

**FIGURE 3:**
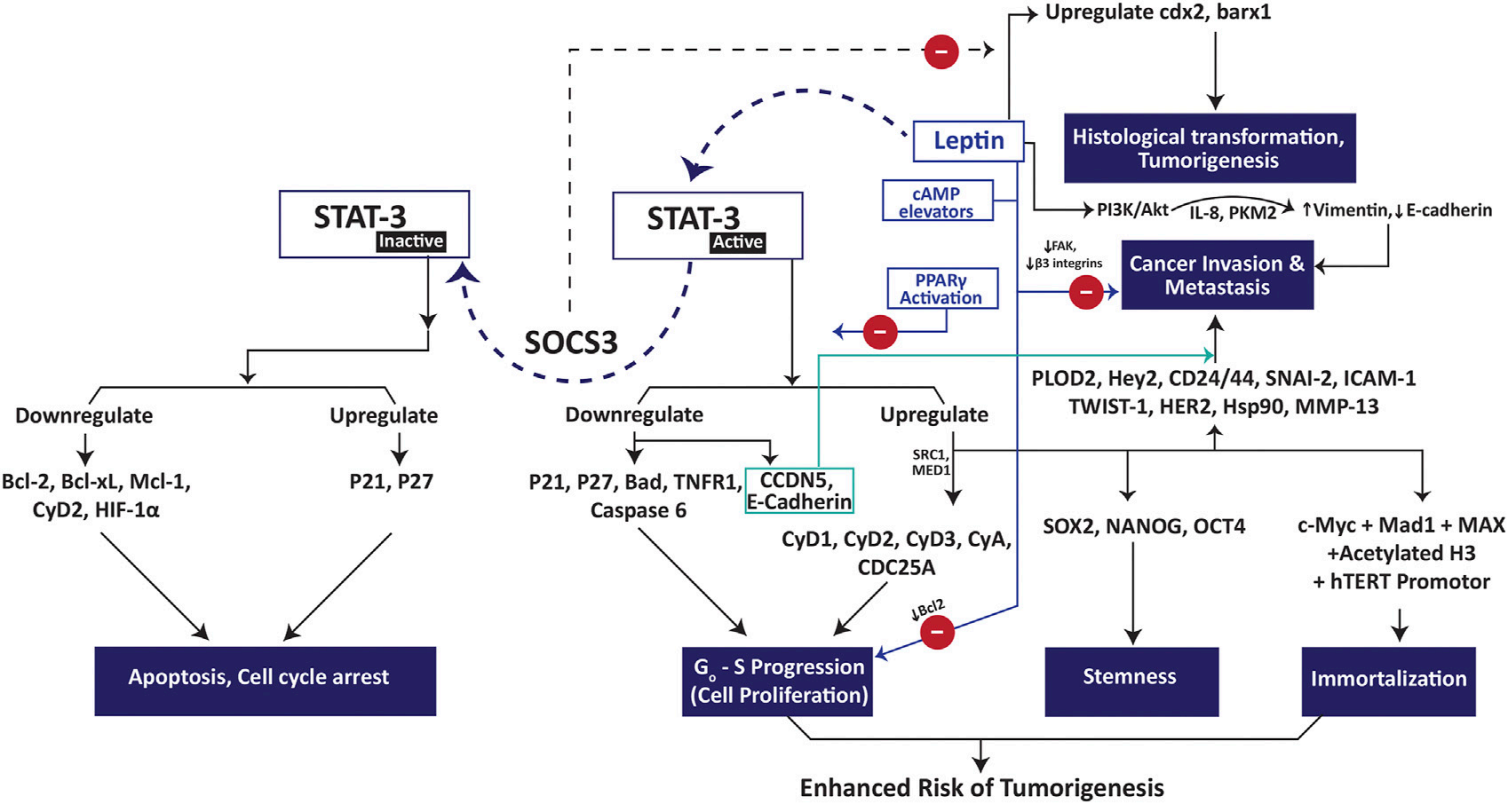
Role of STAT3 in leptin-induced tumorigenesis, cancer cell proliferation and metastasis. Abbreviations: Bad Bcl-2 associated agonist of cell death; Bcl-2 B-cell lymphoma-2; Bcl-xL B-cell lymphoma 2 extra large; CyA cyclin A, CyD1 cyclin D1; CyD2 cyclin D2; CyD3 cyclin D3; Hey2 hairy/enhancer-of-split related with YRPW motif protein 2; HIF-1α hypoxia inducible factor-1 alpha; hTERT human telomerase reverse transcriptase; MAD-1 mitotic arrest deficient-1; MAX myc-associated factor X; Mcl-1 myeloid cell leukemia 1; MMP13 matrix metallopeptidase 13; PLOD2 procollagen-lysine 2-oxoglutarate 5-dioxygenase 3; SOCS3 suppressor of cytokine signaling 3; STAT-3 signal transducer and activator of transcription 3; TNFR1 tumor necrosis factor receptor 1.

The impact of leptin treatment on cancer cell proliferation varies greatly from one type of cells to the other. It exhibited anti-proliferative effect on Chang liver cells, where the proliferation rate in the leptin-treated cells was found significantly lower than the control [42]. Contrarily, it induced proliferation in several cell lines such as the hepatocellular carcinoma cells HepG2 [43], Huh7 [44] and the ovarian adenocarcinoma cells OVCAR3 [45]. When the HepG_2_ and OVCAR3 cells were exposed to different leptin concentrations (0–200 ng/ml), a significantly higher population of cells crossed the G_0_–G_1_ phase in a dose dependent manner and were found in S and G_2_-M phase [43,45]. These findings suggested a direct impact of leptin on DNA synthesis and mitotic division. Leptin triggers JAK/STAT, p44/p42, Erk1/2 and Akt signaling where STAT3 plays pivotal role in exhibiting its proliferative and carcinogenic effects [44,46–49]. The STAT3 role in leptin signaling is further attested by the fact that the inhibition of STAT3 had shown to promote cell cycle arrest, apoptosis and impede tumor invasion [47,50,51]. Moreover, a differential pattern of gene expression was observed in the leptin exposed tissue samples from patients suffering from colorectal cancers where a higher expression of STAT3, Akt1 and MCL-1 was observed in the earlier non-metastatic stages whereas a highly expressed CCND1 and VEGFC genes were observed in the advanced metastatic stages [52]. The role of STAT3 in leptin induced cell proliferation and metastasis is described in Figure 3. It has been suggested that STAT3 mediated mitogenic effects are shared by the signaling through leptin and IL6Rα receptors. A decline in IL6Rα receptors on hepatocytes has boosted the expression of leptin receptors in IL6Rα deficient mice, suggesting the direct involvement of leptin-IL6 interplay in the tumorigenesis of hepatocellular carcinoma [53]. Further, leptin signaling was reported to maintain the stemness in triple negative breast cancer cells via upregulating the self-renewal transcription factors such as SOX2, OCT4 and NANOG via STAT3 signaling [54,55].

The proliferative properties of leptin were reported to get influenced by several cofactors. For instance, leptin has increased proliferation of colon cancer cells *in vitro* however, it failed to support the tumor growth *in vivo* [56]. Likewise, it induced proliferation in androgen-sensitive prostate cancer cells (LNCaP) but failed to influence the proliferative rate of androgen-insensitive PC3 and Du-145 cells [57]. Besides, it inhibited the cell migration and invasion in the mentioned androgen-insensitive cells by activating MAPK pathway, a change that was not observed in androgen-sensitive cells. Further, it activated STAT3 signaling in both types of the cells but with a significant difference. In androgen-insensitive cells, the STAT3 signaling was observed in the earlier phase of the cell cycle with a gradual decline in the later phases. Hence, the leptin-induced STAT3 remained unable to trigger proliferation and precipitated in the blockage of invasion and cell migration in PC3 and Du-145 cells. However, in the androgen-sensitive LNCaP cells, a relatively delayed but persistent activation of STAT3 was observed that boosted the proliferative rate in these cells [57]. Interestingly, leptin associated cell proliferation and migration via STAT3 signaling are reversed if cAMP levels are elevated. For instance, the co-treatment of MDA-MB231 cells with leptin and cAMP elevating agents (8-pCPT-2′-O-Me-cAMP, 8-Br-cAMP) blocked the proliferative effect of leptin and induced apoptosis in cancer cells by downregulating Bcl2 [58]. Such elevation of cAMP also blocked leptin-induced cell migration in MDA-MB231 cells by downregulating FAK and β3 integrins via cAMP/PKA and cAMP/Epac signaling [59]. Moreover, the activation of PPARγ by rosiglitazone has also abrogated leptin induced cell proliferation and metastasis by blocking the activation of MAPK/STAT3/Akt signaling in both the *in vitro* and *in vivo* experiments [60].

In ER negative breast cancer cells (MDA-MB-231 and BT-549), leptin induced the expression of oncogenic enzyme sphingosine kinase 1 (SK-1) via Src family kinase (SFK) and Erk1/2 signaling. However, such upregulation of SK-1 was not observed in ER positive breast cancer cells [61]. Importantly, the role of the suppressor of cytokine signaling (SOCS) should also be considered while discussing the proliferative properties of leptin. The upregulation of SOCS3 inhibits ERK and Jak2 activation [62] and attenuates Akt signaling [63]. The persistent stimulation of leptin receptors precipitates in the higher expression of SOCS3 as a negative feedback mechanism [64]. Moreover, p38-MAPK signaling also results into an upregulation of SOCS3 [65]. Hence the anti-proliferative effect of leptin in H4IIE cells could possibly be attributed to the SOCS3 mediated attenuation of the mitogenic pathways [66]. Contrarily, the irrepressible leptin signaling in SOCS3 deficient mice has exacerbated tumorigenesis in gastric and intestinal epithelium by upregulating cdx2 and downregulating the transcription activities of barx1 [67]. Aside from the mentioned factors, the expression of both methionine adenosyltransferase 1α (MAT 1α) and MAT 1β is necessary for the mitogenic effect of leptin in hepatocellular cancer cells [68].

Long-term exposure of cells to leptin results into the upregulation of human telomerase reverse transcriptase (hTERT) expression by STAT3 mediated binding of c-Myc, Mad1, Max and acetylated H3 with hTERT promotor [69]. The above notion is further confirmed by the fact that leptin-induced cancer cell proliferation is inhibited by 1 alpha, 25-dihydroxyvitamin D3 through miR-498 mediated downregulation of hTERT [70]. hTERT elongates the telomere length to prevent the cells entering senescence after several cell divisions, a process that is generally termed as immortalization. Together with the proliferative properties of leptin, this immortalization of cells after being exposed to leptin for longer may lead to the basis of tumorigenic transformation of cells. STAT3 activates glycoprotein 130 (gp130) subunit of cytokine receptors that further promotes the progression of cells from G_o_ to S phase through upregulating Cyc D1-3, A, cdc25A and downregulating P21, P27 [47,71]. Besides, leptin is reported to induce the expression of Notch1-4, JAG1 and DLL4 in pancreatic cancer cells [72]. The resultant activation of Notch signaling further accounts for the leptin-induced cell proliferation in cancer. More recently, the supportive role of ribosomal protein S27/Metallopanstimulin-1 (MPS-1) in cancer proliferation and invasion is reported in different cancer cells, including glioma [73], colorectal [74] and gastric cancer cells [75]. Leptin is found to upregulate MPS-1 in colorectal cancer cells that further promoted the cell survival and proliferation via activating JNK/c-JUN signaling [74]. Moreover, the role of insulin growth factor-1 (IGF-1) in promoting cancer cell proliferation and invasion is well documented [76]. Leptin treatment had significantly upregulated IGF-1 in breast cancer cells via activated protein-1 (AP-1) transcription factor [77]. The silencing of the three MAPK mitogenic pathways i.e., JNK1/2, p38 MAPK and Erk1/2 abrogated IGF-1 upregulation suggesting the direct involvement of these signaling pathways in leptin-induced IGF-1 upregulation. It is worth mentioning that the extracellular cell survival factors, such as the nutrients, trigger cell proliferation by upregulating JAK/STAT, Erk and PI3K/Akt signaling. Leptin does not appear to halt the proliferation of cells directly. Instead, it exhibits its anti-proliferative effect by attenuating these mitogenic signaling pathways. The evidence to support this notion comes from the findings of Thompson et al. 2011 wherein leptin failed to affect the proliferation of rat H4IIE cells in the serum depleted medium [66]. However, it showed a significant inhibitory effect on these cells in the presence of serum rich medium by upregulating p38-MAPK signaling.

Leptin supports cancer cell survival by inhibiting the apoptotic pathways through downregulating Bad, tumor necrosis factor alpha receptors 1 (TNFR1) and caspase 6 expression [45]. It also supports growing tumor by promoting lymphangiogenesis via upregulating proangiogenic proteins VEGF, IL-1β and leukemia inhibitory factor (HIF) in cancer cells [78–80]. The upregulation of VEGF was found to involve an elevated expression of HIF-1α, NF-κB [81], Notch and IL1 [82] signaling. Further, the mentioned upregulation of HIF-1α involved both the canonical and non-canonical pathways, whereas the NF-κB regulation was mainly attributed to the non-canonical pathway [81]. It is a worth-mentioning finding here that leptin at higher dose induces senescence and G_o_-S phase arrest in chondrogenic progenitor cells (CPC) by inhibiting Sirtuin 1 pathway and upregulating p57/p21 expression [83]. These changes impart osteogenic properties to CPC that may account for the pathophysiology of obesity derived osteoarthritis. The adipocytes released leptin and IL-6 to promote metastasis in breast cancer cells by upregulating PLOD 2 expression via JAK/STAT and PI3K/AkT signaling [84]. In colon cancer cells (HCT116), it promoted Rac-1 and Cdc25 induced invasion, lamellopodia formation and metastasis by activating PI3K and Src kinase pathway [85]. The secretion of leptin is significantly increased from the obesity modified adipose stem cells (obASC) [86] that further promotes EMT in cancer cells, paving a way towards cancer metastasis. Leptin promoted the phosphorylation and inactivation of GSK3β via STAT3 mediated Akt phosphorylation [87]. Moreover, leptin also upregulated metastasis associated protein 1 (MTA1) in breast cancer cells that further contributed to the inactivation of GSK3β via Wnt1 signaling. The mentioned GSK3β inactivation abrogates the formation of its complex with liver kinase B1 (LKB1) and axin, precipitating in the cytosolic accumulation and nuclear translocation of β-catenin. Once inside the nucleus, β-catenin induces the expression of a set of genes including Cyc D1, Cyc D2, Myc, WISP1 (CCN4) and WISP2 (CCN5) to execute EMT. The blockage of Wnt/β-catenin signaling completely abrogated the leptin induced proliferation in breast cancer cells, suggesting its direct involvement in cancer cell proliferation [88]. Interestingly, leptin downregulated the expression of CCN5 in breast cancer cells MCF-7 and ZR-75-1 cells via activating JAK/STAT and Akt pathways [89]. Such downregulation of CCN5 did not affect cell proliferation, but enhanced cell viability, invasion and EMT.

Leptin upregulates the expression of Notch1 by transactivation of epidermal growth factor receptor [90]. This is followed by the recruitment of Notch-1 intracellular domain on the promoter region of survivin. These findings are further supported by the results of another study wherein leptin promoted breast cancer metastasis in mice model by upregulating the levels of Notch 3, Jag1 and survivin [91]. It also enhanced the expression of Notch1, 1-4, JAG1, DLL4 and surviving in pancreatic cancer cells [72]. The resultant upregulation of survivin promotes the migration and invasion of cancer cells, suggesting a direct role of leptin-Notch-EGFR axis in leptin induced metastasis. Interestingly, an increased release of leptin from the pancreatic cancer cells was found to modify the tumor microenvironment further by upregulating the expression of leptin receptors in an autocrine/paracrine fashion [72]. Such upregulation of leptin receptors in also reported in breast cancer cells where the leptin-treatment increased the expression of AdipoR2 receptors in MCF-7 cells [92].

Hey2 (Hairy/enhancer of split related with YRPW motif protein 2) is a transcription factor that is recently reported to play a critical role in cell proliferation and metastasis in hepatocellular carcinoma [93]. Although its expression in hepatocellular carcinoma cells is not linked with leptin elicited effects, its expression along with other pancreatic cancer stem cell markers (including Oct-4, CD133, CD24/44 and ALDH) is significantly increased in leptin treated pancreatic cancer cells [72]. The overexpression of CD24 reduces E-cadherin but at the same time upregulates N-cadherin to accelerate cancer invasion [94]. Likewise, the overexpression of CD44 confers aggressive and distant metastasis in cancer cells [95]. In lung and breast cancer cells, leptin treatment has amplified the levels of soluble ICAM-1 by upregulating ERK, GSK3αβ, JAK1/2, STAT3 and focal adhesion kinase (FAK) signaling [96] without affecting the expression of cell surface ICAM-1. Such elevation of soluble ICAM-1 in cancer cells promotes bone metastasis by inducing osteoclast-like cell formation [96]. It promoted cell migration and metastasis in gastric cancer cells (AGS, MKN-45) by enhancing the expression of ICAM-1 (both soluble and surface) via Rho/ROCK signaling [97]. The stromal cell derived leptin is reported to support cancer metastasis in estrogen receptor positive [98] and triple negative [99] breast cancer cells by enhancing EMT via upregulating SERPINE 1, IL-6, MMP-2, SNAI 2, TWIST 1, vimentin and downregulating E-cadherin [100]. The leptin induced EMT in breast cancer cells was reported to involve an upregulation of IL-8 [101] and pyruvate kinase M2 (PKM2) [100] via PI3K/Akt signaling. These findings were further confirmed by the fact that the silencing of leptin signaling in highly metastatic MDA-MB-231 cells had resulted into MET via upregulating E-cadherin and downregulating vimentin [54]. Leptin also induced EMT in lung cancer cells by downregulating E-cadherin, keratin and upregulating vimentin and the transcription factor ZEB-1 via Erk signaling [102].

Leptin exhibits a dose and time dependent activation of mTORC and its downstream protein p70S6 kinase that precipitates into an increase in cell proliferation and upregulation of cyclooxygenase 2 (COX2), transforming growth factor-beta (TGF-β), monocyte chemoattractant protein 1 (MCP-1) and cytokine induced neutrophil chemoattractant-1 (CINC-1) [103]. Likewise, leptin upregulates the expression of TNF-α in macrophages [104]. The activated p70S6 kinase is reported to boost cancer metastasis and invasion through IL-6 mediated EMT in squamous cell carcinoma [105]. Moreover, the upregulation of TGF-β, TNF-α and COX-2 promote EMT in different cancers that results into the poor prognosis of cancer therapy [106–110]. The upregulation of heat shock protein 27 (hsp27) in leptin treated MCF-7 cells may also account for the leptin-induced metastasis [111], since the role of hsp27 is well documented in suppressing apoptosis and promoting cancer cell invasion and metastasis [112]. Leptin also promoted cancer growth and progression by STAT3-mediated upregulation of MMP-13 [113], HER2 and hsp90 [114] in pancreatic and breast cancer cells respectively.

It is recently reported that the treatment of triple negative breast cancer cells with leptin has upregulated the expression of oncogenic and chemotherapy-resistant genes including ITGB3 (integrin β3), LL22NC03, ABCB1 (p-glycoprotein 1), WNT4 (WNT4 protein), TBC1D3 (TBC1 domain family member 3E/3F), ADHFE1 (alcohol dehydrogenase, iron containing 1) and RDH5 (11-cis retinol dehydrogenase) [115]. Likewise, it promoted tumorigenesis in ovarian epithelial cells by enhancing the expression of histone deacetylase (HDAC) class I and II [116]. It is thereby suggested that leptin is not only involved in tumorigenesis, it also contributes to the resistance of cancer cells against chemotherapy [115,117]. For instance, the cytotoxic potential of 5-fluorocracil is countered by leptin by attenuating the pro-apoptotic proteins Bax and caspase 3 [118]. It is also important to state that the major tumorigenic aspects of cancer biology such as the immunosuppression, malignant transformation, angiogenesis, and metastasis involve a complex exosomal cell-to-cell communication between cancer cells [119]. Leptin is reported to enhance such exosomal cell communication in estrogen positive MCF-7 and triple negative MDA-MB-231 breast cancer cells via hsp90 mediated post-transcriptional increase in the levels of tumor susceptibility gene 101 (Tsg 101) protein [120].

In addition to its role in cell proliferation and metastasis, leptin also supports the survival of cancer cells under nutrient-deficient tumor microenvironment by promoting autophagy. It is reported to induce autophagy in breast cancer and hepatocellular carcinoma cells by enhancing the expression of LC3II, Atg5, Beclin1 and downregulating p62 expression [121,122]. Such induction of autophagy by leptin involved the activation of p53 and AMPK/FoxO3A pathway. Leptin induced an upregulation of p53 in HepG2 and MCF-7 cells without affecting the p53 mRNA levels [123]. Instead, the upregulation was found to involve the regulation of p53 ubiquitination via ubiquitin specific protease-2. In breast cancer cells, the expression of autophagy genes was found to involve estrogen receptors ERα signaling, since the inhibition of ERα abrogated leptin-induced autophagy [121]. The leptin signaling also suppresses mitochondrial respiration in cancer cells, an act that further promotes invasion, metastasis and enhances the survival of these defiant cells under hypoxic conditions [124,125].

### Resistin/Adipose Tissue Specific Secretory Factor (ADSF)/FIZZ3

The expression of resistin was found significantly higher in estrogen negative breast cancer patients and was reported as a potential biomarker for early cancer detection [29]. The positive correlation between serum resistin levels, cachexia and metastasis in gastroesophageal cancers affirms its direct involvement in cancer pathogenesis [27]. Further, its higher expression is found associated with poor prognosis, nodal metastasis, and advanced cancer stage in colorectal cancer patients [126]. It is important to state that its expression was negatively associated with high density lipoprotein cholesterol (HDL-C) in colorectal cancer patients because of a possible interaction of its release with HDL-C [127].

Mechanistically, resistin was found to promote invasion, migration and cellular protrusions in cancer cells by inducing the reorganization of F-actin filaments [128]. In the breast and ovarian cancer cells, resistin upregulated the expression of SNAIL, SLUG, ZEB1, TWIST1, fibronectin, vimentin and downregulated claudin-1 and E-cadherin via AXL tyrosine kinase receptor signaling [128–130]. Further, it induced the adhesion molecules ICAM-1 and VCAM-1 in hepatocellular carcinoma cells SK-Hep1 [131] and colorectal cancer cells HCT-116 [132] through NF-κB signaling. These adhesion molecules tend to attach the cancer cells with the HUVECs, a process that may account for intravasation. It also potentiated the nuclear translocation of SNAIL to induce EMT, an important pre-requisite for SNAIL induced mesenchymal transition. Further, the resistin induced EMT was completely abrogated after silencing adenyl cyclase associated protein 1 (CAP-1), suggesting a direct involvement of CAP-1 in resistin induced metastasis [128]. Moreover, the significant role of CXC chemokine receptor 4 (CXCR4) in lymphangiogenesis and cancer metastasis is well documented. Resistin is reported to enhance the expression of CXCR4 in gastric cancer cells via TLR4 receptor signaling [133]. Further, the resistin induced upregulation of CXCR4 is abrogated by blocking p38-MAPK and NF-κB pathway, suggesting the involvement of these signaling proteins in resistin induced CXCR4 upregulation.

The resistin treatment enhanced stemness in cancer cells by upregulating the stemness markers aldehyde dehydrogenase-1 (ALDH1), CD44, Oct4, Nanong and Sox2 and downregulating CD44 cell surface expression [130,134]. It also promoted cell proliferation in gastric cancer cells by upregulating the expression of hTERT gene [135]. Apart from its impact on cell proliferation and EMT, it also exhibits chemotherapy resistance. For instance, it was found to exert doxorubicin resistance in breast cancer cells by blocking the chemotherapy-induced apoptosis via activating STAT3 signaling [134]. Likewise, the efficacy of dacarbazine was also attenuated by resistin through upregulating caveolin-1 [136] in melanoma cells [136]. It also induced accumulation of LC3 in breast cancer cells to support their survival under drastic chemotherapeutic conditions via activating autophagic pathways [137]. It was found that the treatment with resistin culminates into autophagy by upregulating BECN1, LAMP1, LC3B-II and downregulating SQSTM1. Such activation of autophagic pathways was completely abrogated by blocking the JNK and AMPK/mTOR/ULK1 signaling [137], suggesting the direct involvement of these two pathways in resistin induced autophagy.

### Visfatin

Visfatin is reported to induce cell proliferation in different cancer cells such as melanoma and breast cancer cell [138–140]. Like leptin, visfatin also stimulates a number of cell signaling pathways that support cell proliferation and survival, such as the PI3K/mTOR, ERK1/2, p38 MAPK, JAK/STAT and JNK [141–145]. This proliferative effect of visfatin is conferred by its ability to stimulate notch 1 signaling through activating the p65 unit of NF-κB [146]. It induces cell survival in melanoma cells by inhibiting p53 dependent apoptosis [140]. The intensity of cancer invasion and metastasis in gastric [147] and colorectal cancer patients [148] was found correlated with their higher serum visfatin levels. Its high levels were also found correlated with the ability of small cell lung cancer (SCLC) cells to metastasize into brain via transendothelial migration across the blood brain barrier [149]. Such migration of SCLC cells across the brain endothelial cells was found to involve upregulation of chemokine ligand 2 (CCL2). It is worth mentioning here that the CCL2-CCR2 signaling plays a crucial role in both intravasation and extravasation through the recruitment of CCR2 positive myeloid cells [150]. These myeloid cells help the recalcitrant CCL2 positive cancer cells to enter into blood/lymph vessels from their site of origin and then extravasate to a new location. Likewise, it also imparts motility in cancer cells via NF-κB and IL-6 signaling [151] and promotes EMT by facilitating the nuclear transport of the transcription factors SNAIL and β-catenin [148]. Moreover, it also inhibits its binding with GSK3β in cytosol to prevent its’ ubiquitination [148]. The ascites-driven visfatin was found to promote metastasis in ovarian cancer cells by inducing lamellipodia and filopodia via Rho/ROCK signaling [149]. In non-small cell lung cancer cells, it induced resistance to the chemotherapeutic effect of doxorubicin by Akt induced upregulation of multidrug resistance protein-1 (MRP-1) [152].

### Omentin

Since omentin-1 (intelectin-1) is the major adipokine from the visceral fat (that is accumulated in the omentum), its emerging significance in the metastasis of ovarian and gastric cancers is well reported [153,154]. The omentum is covered with a thin layer of mesothelial cells. To metastasize, the cancer cells have to bind and penetrate through the mesothelium. At first, the anti-metastatic potential of omentin-1 tries to block the penetration of the cancer cells through mesothelium, probably by involving lactotransferrin (LTF). The interaction of LTF with its receptor is crucial for the up-regulation of matrix metalloproteinase 1 (MMP1). By inhibiting LTF binding with its receptor LRP-1 (low intensity lipoprotein receptor related protein 1), omentin-1 attenuates the upregulation of MMP1 in ovarian cancer cells [153]. As MMP1 plays a significant role in invasion of cancer cells through extracellular matrix, its down regulation in ovarian cancer cells accounts for the anti-metastatic potential of omentin-1 [153]. Likewise, omentin-1 blocks the PI3/AKT mediated up-regulation of NF-κB signaling [154]. Since NF-κB suppresses the expression of hepatic nuclear factor 4 alpha (HNF4α), omentin-1 upregulates its expression in gastric cancer cells via attenuating NF-κB signaling [154]. HNF4α blocks the metastatic effects of β-catenin by inhibiting its nuclear translocation, a mechanism that accounts for the anti-metastatic effect of omentin-1 in gastric cancer cells [154]. However, the cancer cells escape omentin-1 mediated anti-invasive barrier by downregulating its expression in mesothelial cells through TNF-α signaling [155]. Likewise, the ovarian cancer cells upregulate fibronectin in the mesothelial cells of omentum via TGF-β dependent Rac1/SMAD signaling. Such upregulation promotes invasion and metastasis in these cells, departing the anti-metastatic effect of omentin-1 [156].

### Adiponectin

As mentioned earlier, adiponectin exhibits promising anti-proliferative and pro-apoptotic properties. Such potent anti-cancer potential of adiponectin is also reflected from the fact that the expression of adiponectin receptor 1 and 2 is downregulated in highly metastatic cancers. For instance, the tissue samples from colorectal cancer patients with nodal metastasis exhibited a significant downregulation of adiponectin receptors 1 and 2 as compared to the normal tissue samples [157]. Such downregulation of adiponectin receptors could be considered as a survival mechanism in these metastatic cancer cells to evade the anti-proliferative and pro-apoptotic effect of adiponectin. Adiponectin attenuates cancer metastasis by upregulating the expression of tumor suppressor protein LKB1 [158]. LKB1 inhibits mTOR complex 1 (mTORC1) by phosphorylating tuberous sclerosis proteins 2 (TSC2) via phosphorylating AMP-activated protein kinase (AMPK) [158,159]. It is worth mentioning here that the blockage of mTORC1 by LKB1 does not only depend upon TSC2 activation as it is equally suppressing mTORC1 in TSC2 lacking cells. Such TSC-2 independent blockage of mTORC1 by activated AMPK involves a direct phosphorylation of raptor protein on two distinct positions i.e., serine 722 and serine 792 which precipitates into the binding of raptor with 14.3.3 protein [160]. By blocking mTORC1 via LKB1, adiponectin suppresses the activation of its downstream protein P70SK [158] which is a key regulator of cell invasion and metastasis in many different types of malignancies [161,162]. Importantly, such antimetastatic effect of adiponectin is not observed in ER positive breast cancer patients. In such patients, the EAα-LKB1 interplay attenuates the LKB1/AMPK mediated inhibition of mTORC1 with a subsequent promotion of invasion and metastasis in ER positive breast cancer cells [163]. Hence, the LKB1 mediated blockage of p70SK by adiponectin may account for its anti-metastatic properties. Moreover, adiponectin inhibits the intracellular accumulation of β-catenin via blocking GSK3β phosphorylation [164]. Such inhibition of β-catenin may account for the anti-metastatic potential of adiponectin.

Adiponectin inhibits the proliferation of cancer cells by downregulating STAT3 and upregulating SOCS3 [165,166]. Further, its inhibitory effect on the proliferation, invasion and metastasis of cancer cells could also be attributed to the attenuation of PI3K/Akt [167], Erk 1/2 [168–170], mTOR/S6 [171] and NF-κB signaling [172]. It also downregulates the expression of Cyc D1 via blocking the Akt-mediated phosphorylation of GSK3β [173]. Likewise, it downregulates c-Myc, Cyc D, Bcl2 in cancer cells with an upregulation of p53, p21, p27 and Bax [170,174,175]. In colon cancer cells, adiponectin treatment halted the proliferation by activating AMPK signaling. Further, it also suppressed the expression of SREBP1c that contributed to its antiproliferative effect in colon cancer cells [175].

### Angiopoietin

The impact of Ang 1 expression on cancer metastasis stands dubious since a number of studies have reported “conflicting’’ results regarding its pro-metastatic or anti-metastatic potential. Owing to the fact that the cancer cells tend to invade the surrounding tissue by breaking their cell-cell and cell-ECM attachments, the mediators that strengthen such intercellular junctions suppress such invasion and metastasis. Kitajima and colleagues have recently reported that the overexpression of Tie2 receptors in oral squamous cell carcinoma bolster the cell-cell and cell-matrix attachment [176]. In a subsequent study, Kitajima and colleagues claimed a significant enhancement in the tie2-induced cell-cell and cell-matrix attachment by Tie2/Ang 1 interaction [177]. They also suggested a negative correlation between nodal metastasis and Tie2 and/or Ang 1 expression [177]. In another parallel study, the Ang 1 knockout mice showed significant increase in the metastasis of breast and melanoma cells to the lung tissue without affecting the growth of the primary tumor [178]. Since the colonization of cancer cells in lungs was significantly increased in Ang 1 knockout mice after direct intravenous injection of melanoma cells, it was suggested that Ang 1 deficiency might have promoted the last stage of metastasis i.e., the extravasation in the knockout mice [178]. Likewise, Ang 1 and vasculoid (Tie2 agonist) blocked the *trans*-endothelial migration of colon, breast and renal cancer cells *in vitro* [179]. Moreover, vasculoid also delayed the spread of breast cancer cells to lungs [179]. In contrast, the expression of Ang 1 in cancer cells through adenoviral vector-mediated transfection has been reported to induce cancer metastasis via overexpression of β1 integrin and CD44 [180,181]. The expression of β1 integrin and CD44 was found to increase the attachment of gastric cancer cells with the extracellular matrix that might have increased their invasive potential [180]. The findings of Holopainen and coworkers suggested that Ang1 expression enhances vascular enlargement, without significantly affecting vascular density. Such enlargement of blood vessels assists in the extravasation of cancer cells by promoting their dissemination in blood [181]. Summarizing, Ang1 exhibits a dual role in cancer metastasis. In the early stages, it exerts an inhibitory effect on cancer invasion by strengthening the cell-cell junctions via Tie2 receptors. However, once the cancer cells succeed in invading the extracellular matrix via EMT, the Tie2/Ang 1 signaling promotes the *trans*-endothelial migration of these cells via bolstering their attachment with endothelial cells, a prerequisite for both intravasation and extravasation. It is important to mention here that the pulmonary metastasis in Lewis lung carcinoma cells (LLCC) as well as in the TA3 mammary carcinoma cells was found to involve VEGF and Ang 1 induced angiogenesis [182]. VEGF and Ang 1 promote angiogenesis in these carcinoma cells through ERK 1/2 and Akt pathways. Ang 3 blocks pulmonary metastasis in LLCC and TA3 carcinoma cells by suppressing the expression of both VEGF and Ang 1 [182]. The binding of Ang3 with the cell surface through heparan sulfate proteoglycans was found mandatory to inhibit Ang 1 and VEGF mediated angiogenesis and metastasis in LLCC and TA3 cells [182].

Ang2 is reported to promote cancer metastasis by activating EMT in breast cancer [183] and oral squamous cell carcinoma [184]. In Tie-2 deficient cells, Ang 2 promotes the activation of ILK, GSK3β and Akt via its binding with α_5_β_1_ integrin [183]. This activation of ILK and GSK3β induces SNAIL expression, downregulates E-cadherin and promotes EMT to induce metastasis [184]. An estrogen depletion therapy in ER + breast cancer patients may instigate cancer recurrence and metastasis via Ang 2 involvement. Ang 2 exhibits such revival of dormant ER + breast cancer cells in the vascular niche of bone marrow either by directly inhibiting Ang 1-Tie2 signaling, or via stimulating surface integrin β1 receptors in these dormant cancer cells [185].

### Angiopoietin Like Proteins (ANGLP)

Several reports have correlated the expression of ANGLP 2 with cancer metastasis. For instance, the overexpression of ANGLP 2 was found associated with the lymph nodal metastasis in the clinical samples from the surgically treated non-small cell lung cancer patients [186]. Likewise, ANGLP 2 was reported to promote carcinogenesis and metastasis to distant organs in mice by promoting EMT [187]. The expression of transcription factors such as the nuclear factor of activated T cells (NFATc), activating transcription factors (ATF) and c-JUN in aggressive tumor cells result into the upregulation of ANGLP 2 [188]. Further, HIF-1α is recently reported to upregulate ANGLP 2 expression in hypoxic osteosarcoma cells [189]. The upregulation of ANGLP 2 further promotes motility and invasion in the cancer cells in either an autocrine or a paracrine fashion [188]. ANGLP 2 promotes metastasis though facilitating angiogenesis as well as glycolysis via upregulating VEGFA, Ang 2 and hexokinase 2 [189]. Further, it promoted intravasation in osteosarcoma cells via upregulating p38 MAPK signaling, MMP as well as integrin α_5_β_1_ [190].

Earlier, ANGLP 4 was reported to inhibit metastasis in melanoma cells by inhibiting cancer cell motility and reducing vascular permeability to the invading cells [191]. However, the subsequent studies have reported the opposite findings. ANGLP 4 is found to promote lung metastasis in breast cancer cells via blocking endothelial to endothelial cell interactions [192]. The inhibition of these endothelial cell interactions is necessary for the *trans*-endothelial migration of cancer cells during the intravasation and extravasation. Since TGFβ was found to upregulate the expression of ANGLP 4 in breast cancer cells, the lung metastasis induced by TGFβ was found to involve ANGLP 4 mediated increase in vascular permeability [193]. Likewise, HIFα also exhibited its metastatic potential by upregulation of ANGLP 4 [192]. ANGLP 6 is found within the blood vessels of the colorectal cancer patients. It is reported to bind with tumor cell specific integrin α6 and E-cadherin on the surface of the tumor cells that precipitates into the liver metastasis of colorectal carcinoma cells [194].

### Chemerin

Chemerin exerts its biological functions through G protein coupled receptor 1 (GPR 1) and chemokine like receptor 1 (CMKLR 1) [30]. The expression of chemerin and its receptors are highly upregulated in adipose tissue and the recent research has reported a positive correlation between circulating chemerin levels and obesity [195]. Its role in promoting the invasion and metastasis of cancer appears to be dubious, since it exhibits both tumor suppressive and promoting effects that depends upon the type of the disease. For instance, it is reported to exhibit cancer suppressive effect in hepatocellular carcinoma [196]. The downregulation of chemerin expression in malignant adrenocortical carcinoma [197] and acute myeloid leukemia [198] could be assumed as a survival tactic of the cancer cells to evade the immunosuppressive effect of chemerin. Contrarily, its expression is positively associated with malignancy in non-small cell lung carcinoma [199] and gastric cancers [200]. The complexity of chemerin role in breast cancer could be explained by the conflicting findings of two recent studies. Pachynski and coworkers have reported that the expression of chemerin in the breast tumor microenvironment suppresses the progression of disease by promoting the infiltration of immune cells [117]. Contrarily, El-Sagheer *et al* has reported a comparatively higher expression of chemerin in malignant tissue, suggesting a significant association of chemerin with poor prognosis in breast cancer patients [201].

First, we focus on the cancer suppressive potential of chemerin that could mainly be attributed to its immunomodulatory and chemoattractant potential. Binding of chemerin with its receptors CMKLR1 and GPR1 has reported to induce receptor internalization and deactivation [202]. Such internalization of the G-protein coupled receptors further recruits β-arrestin 1 and 2 with a subsequent activation of β-arrestin 2 dependent ERK1/2 activation [202]. It is worth mentioning here that the role of β-arrestin 2 in cancer stands dubious till date. Although a few reports have suggested its tumor promoting potential in renal [203] and breast cancer [204], a considerable data suggests that the activation of β-arrestin 2 inhibits tumorigenesis. By acting as a corepressor of androgen receptors, β-arrestin 2 downregulated FOXO1 with subsequent inhibition of cell proliferation in prostate cancer cells [203,205]. Moreover, its depletion/less expression has resulted into aggressive cancer invasion with poor prognosis in non-small cell lung cancer [206] and hepatocellular carcinoma [207]. Importantly, the β-arrestin mediated activation of ERK1/2 promotes the cytosolic functions of ERK1/2 by impeding its nuclear translocation [208]. Contrarily, the AMP-PKA mediated activation of ERK1/2 promotes the nuclear translocation of ERK1/2 with subsequent expression of proliferative genes. Hence, the chemerin-β arrestin signaling may contribute to its tumor suppressive potential. Besides, chemerin dependent activation of β-arrestin 1 and 2 may account for the dubious role of chemerin in different types of cancers as mentioned above.

Chemerin is reported to upregulate the expression of serum response factor (SRF) in lymphocytes and gastric carcinoma cells through Rho/ROCK signaling [209]. Importantly, the overexpression of SRF was found associated with poor prognosis and shorter survival rates in patients with gastric carcinoma [210], bone and prostate malignancies [211]. SRF exerts its cellular effects either by the activation of ternary complex factor (TCF) [212] or myocardin-related transcription factor (MRTF) dependent gene expression [213]. Besides an established role of SRF and MAPK signaling in cell proliferation, chemerin signaling through CMKLR1 and GPR1 had resulted into different response in different cell lines. It stimulated Rho/MAPK/SRF signaling in human lymphocytes and gastric carcinoma cells, however it exhibited different pattern of downstream signaling in the mentioned cell types [209]. In lymphocytes, the mentioned chemerin-induced expression of SRF upregulated ERG1 and cFOS with an antiproliferative effect at the concentration of 10 nM, however such effect was not observed at the other tested concentrations (1, 30 and 100 nM). Contrarily, chemerin-induced SRF upregulated vinculin instead of ERG1 and cFOS in gastric carcinoma cells with no effect on cell proliferation at all tested concentrations [209].

Since ERG1 inhibits cell proliferation and promotes apoptosis through activating p53 pathway [214,215], chemerin induced upregulation of ERG1 could possibly account for its tumor suppressive properties. However, the role of cFOS and vinculin in cancer differs in different types of malignancies. The upregulation of cFOS by chemerin signaling is reported in a number of cell lines [209,216,217]. Although cFOS was initially believed to act as an oncogene [218,219], its role in cancer progression stands dubious since several studies have reported its cancer suppressive potential in hepatocellular carcinoma [220], ovarian [221,222] and prostate cancer [223]. Likewise, vinculin is an adhesion related protein whose expression is negatively associated with cancer metastasis and poor prognosis [224], its upregulation in gastric cancer patients is positively associated with poor prognosis and shorter survival [225]. As we have mentioned the opposite role of chemerin in human lymphocytes and gastric carcinoma cells with its cancer suppressive effect in the former and cancer supportive potential in the later [209], the chemerin-induced upregulation of cFOS and vinculin could possibly be attributed to the mentioned dual behavior of chemerin in hepatocellular [220,226] and gastric carcinoma [225] respectively. Moreover, chemerin is found to inhibit invasion and metastasis of hepatocellular carcinoma cells by upregulating PTEN and downregulating p-Akt activities [196].

The role of the polarization of naive macrophages to either M1 or M2 phenotype in cancer progression and metastasis has been established already [227]. While M1 macrophages exhibit tumor retardation and impede the progression and metastasis of cancer cells, the M2 phenotype exerts the opposite effect. Because of their anti-inflammatory and pro-tumor attributes, a higher infiltration of cancer tissue with M2 macrophages is reported to be associated with poor prognosis and less survival in different cancers [228–230]. Contrarily, the pro-inflammatory and tumor suppressive properties of M1 macrophages help these macrophages to suppress the invasion and spread of cancer. For instance, a higher infiltration of M1 phenotype in cancerous tissue (M2/M1 < 3) showed better survival with significant inhibition of cancer progression in colon cancer patients [231]. Out of the two mentioned macrophages populations, chemerin exerts a chemoattractant effect on CMKLR1 expressing M1 macrophages selectively [232]. Hence, the chemerin-induced infiltration of M1 macrophages in the cancerous tissue could be attributed as one of the mechanisms by which chemerin exerts its cancer suppressive effect. In addition, the chemerin enhanced infiltration of CMKLR1 positive natural killer cells (NK) in melanoma with a relatively reduced colocalization of plasmacytoid dendtritic cells (pDC) and myeloid-derived suppressor cells resulted into the inhibition of tumorigenesis by bolstering the immune response [233]. The downregulation of CMKLR1 receptors attenuated this tumor-suppressive effect of chemerin, indicating a major contribution of NK cell infiltration in the chemerin induced tumor suppression.

Now we discuss the properties of chemerin that support the invasion and spread of cancer cells. Being a chemoattractant adipokine, chemerin has a strong potential to bolster the obesity associated chronic inflammation [234]. This notion is further attested by the fact that the serum chemerin levels are positively associated with inflammatory cytokines and glycated hemoglobin and both of these factors are associated with chronic inflammation [235,236]. These inflammatory cytokines, particularly IL-6, IL-1β and TNFα upregulate the expression of CMKLR1 receptors on the surface of the endothelial cells [237]. The CMKLR1 signaling was shown to exert angiogenic effect on the endothelial cells via MAPK and PI3K/Akt dependent upregulation of MMP2 and MMP9 expression [237]. Chemerin upregulated the VEGF expression in endothelial cells [238] and gastric cancer cells along with an upregulation of p38 MAP kinase, ERK1/2 and MMP7 [239]. The angiogenic potential of chemerin was also confirmed in the mice model [240]. It was reported to induce proliferation, migration, and tube formation in HUVEC cells through phosphorylation of Akt and p42/44 ERK. Further, the mentioned effects were completely abrogated by silencing CMKLR1 receptors in HUVEC cells that suggested a strong angiogenic potential of chemerin. Apart from its chemoattractant effect on macrophages and NK, it also exerts significant migration and recruitment of pDC through CMKLR1 receptors [241]. Although a further study is required to authenticate such chemerin-induced infiltration of pDC in cancers, it is important to mention here that the tumor microenvironment modifies the tumor infiltrating pDC. The downregulation of IRF7 pathway [242] as well as other factors of tumor microenvironment such as PGE2 and TGFβ [243] abrogate the ability of pDC to release INFα. The lack of INFα results into an immunosuppressive tumor microenvironment that facilitates the progression and metastasis of cancer cells as reported in patients with head and neck cancer [244].

### Neutrophil Gelatinase-Associated Lipocalin/Lipocalin 2

Lipocalin 2 (LCN2) is a glycoproteins that performs various physiological functions such as the remodeling of adipose tissue, iron homeostasis, immune responses and the transport of hydrophilic substances across the cell membrane. The levels of LCN2 are positively associated with obesity [245]. Although a few studies have reported its antiproliferative and anti-metastatic role in cancer [246,247], a considerable part of the literature presents an entirely different picture with LCN2 as a promoter of cell proliferation, invasion, metastasis and poor prognosis in different types of cancers (Please see Supplementary Material Table 1). With a 27-fold higher expression in pancreatic cancer cells (compared with normal ductal cells) and an elevated serum level in 94% of the pancreatic cancer patients, LCN2 is reported as an early detection marker for pancreatic malignancies [248]. Likewise, its expression is upregulated in the advanced stages (stage II and III) of breast cancer patients suggesting its critical role in cancer invasion and nodal metastasis [249].

The expression of LCN2 is significantly contributed by the regulatory T cells of the tumor microenvironment. On one side, these regulatory T cells exhibit an immunosuppressive effect that favors the survival of cancer cells within the tumor microenvironment [250]. Additionally, the FoxP3+ regulatory cells release pro-inflammatory cytokines such as IL17, IL1β and TNFα that help recruit NF-κB on the LCN2 promotor region [251]. Hence, the release of these pro-inflammatory cytokines from the regulatory T cells of the tumor microenvironment directly upregulates the expression and levels of LCN2 in the cancer cells. Moreover, the C-X-C motif chemokine receptor 7 signaling in cancer cells upregulates the expression and release of MMP2 and MMP9 into the extracellular matrix [252]. LCN2 treatment has itself resulted into an increased expression of MMP1, MMP3 and MMP9 along with an upregulation of IL-1β, IL-6, IL-8, ICAM-1 and C-X-C motif chemokine 2/monocyte chemoattractant protein 1 (MCP-1) in pancreatic cancer stellate cells [253]. The release of MMP9 and LCN2 into the extracellular space leads to the formation of LCN2-MMP9 complex into the extracellular matrix. In such complex, LCN2 activates MMP9 [254], increases its stability by preventing its degradation [255] and promotes its enzymatic activity [256]. LCN2-MMP9 complex has been found to promote proliferation and metastasis in various cancer cells such as the gastric [255], breast [257] and thyroid carcinoma cells [258]. Higher levels of LCN2-MMP9 complex are found in the urine samples of cancer patients suggesting the significance of the extracellular LCN2-MMP9 complex in the proliferation and metastasis of cancer cells [259]. The role of LCN2 in cancer metastasis is further confirmed by the fact that its deficiency in murine breast cancer cells had resulted into a significant reduction in the size and metastasis of the primary tumors with a reduced MMP9 levels in blood [260]. Likewise, the downregulation of NF-κB in human thyroid carcinoma cells had resulted into a failure in growing the cancer cells and inability to grow the xenograft via downregulating LCN2 [261]. Contrarily, its upregulation has produced opposite effects in human PANC1 pancreatic carcinoma cells with an aggressive invasion and metastasis [262]. LCN2 promotes EMT by upregulating mesenchymal markers such as fibronectin and vimentin in breast cancer cells while inhibiting the epithelial marker E-cadherin [249]. Silencing of LCN2 expression in the highly aggressive breast cancer cell line MDA-MB-231 has resulted into a diminished mesenchymal transition that has further confirmed the involvement of LCN2 in promoting EMT [249].

The upregulation of Ras/MAPK signaling in Ras-transformed cells promotes the phosphorylation and degradation of E-cadherin [263]. LCN2 was reported to block EMT in Ras-transformed 4T1 cells by antagonizing such degradation of E-cadherin [263]. It also exhibited anti-metastatic and antiangiogenic effect in these cells via inhibiting Ras-induced VEGF and upregulating caveolin-1 and anti-angiogenic thrombospondin. Contrarily, it promoted invasion, metastasis and EMT in non Ras-transformed 4T1 cells via attenuating PI3K/Akt pathway [264]. It is important to mention here that the PI3K/Akt signaling promotes cancer cell survival by inhibiting tumor suppressor tuberous sclerosis complex 2 (TSC2). However, it exhibits both suppressive and promoting effects on cancer cell invasion and metastasis. For instance, PI3K/Akt was reported to suppress cancer metastasis in breast cancer cells [265] and promote cell migration and invasion in esophageal [266] and renal carcinoma [267]. The question arises as to how the LCN2-induced attenuation of PI3K/Akt pathway could account for the pro-metastatic effect of LCN2. It is recently reported that the underlying mechanism of Akt-induced metastasis involves the upregulation of MMP2 and MMP9 [267]. As the MMPs are upregulated by LCN2 itself [253] as well as by other players of the tumor microenvironment such as the CXC motif chemokines [252], the LCN2 mediated suppression of Akt could not impede the invasion and metastasis of cancer cells. Further, the downregulation of Akt signaling could promote metastasis via TSC2 mediated activation of Rho/GTPase signaling [268]. A higher expression of TSC2 in breast cancer patients was positively associated with aggressive metastasis and poor survival rate [268]. Since Akt boosts the phosphorylation and degradation of the nuclear factor of activated T-cells (NFAT) through ubiquitin ligase MDM2 [269], the downregulation of Akt signaling could also promote metastasis by enhancing the transcriptional activity of NFAT [269].

Once synthesized within the cancer cells, LCN2 is released into the extracellular matrix where it forms LCN2-catecholate-Fe^3+^ (LCF) complex. Moreover, the LCF complex enters the cancer cells through the LCN2 receptors, increasing the intracellular iron concentration. It is important to mention here that the hydroxy groups of catechol reduce the iron ions from ferric to ferrous state in the absence of LCN2. This could further precipitate into the generation of ROS that could be detrimental for the survival of the cancer cells. Since LCN2 binds with catechol through O-sulfonation or O-hydroxylation, it prevents the reduction of iron ions into the ferrous state [270]. Thereby it blocks the generation of ROS, a change that prevents the anchorage free cancer cells from the caspase-independent ROS mediated cell death mechanisms [271]. Additionally, the increase in cytosolic iron level inactivates the pro-apoptotic Bcl-2-like protein 11 (BIM) [272]. The increase in intracellular iron stores in breast cancer cells has resulted into a hepcidin mediated degradation of ferroportin, attenuating iron efflux and promoting cancer cell survival and proliferation [273].

### Uridine

Uridine is a nucleoside involved in many physiological functions such as the homeostatic regulation of body temperature, glycogen storage and energy balance, however its physiological role has not gained that much attention of the research community as compared to adenosine. It is synthesized in hepatocytes and adipocytes where the adipocytes are the major source of uridine synthesis under fasting conditions [274]. Plasma uridine levels increase during fasting that attenuate body metabolism through hypothalamic pathways, thus reducing the body temperature [274]. Contrarily, a meal induces the secretion of bile into the small intestine, resulting into the biliary clearance of uridine which precipitates into a postprandial decline in plasma uridine levels. Interestingly, such postprandial decrease in plasma uridine levels is not seen in obese individuals. The results from a recent study has shown a postprandial increase in plasma uridine levels in obese individuals [275]. Since the plasma levels of uridine are increased in obesity, we will now focus on the possible role of uridine and its salvage pathway in supporting/promoting cancer cells.

Till date, no specific receptors for uridine have been identified. However, most of its physiological functions are conferred through G protein coupled purine receptors such as P2Y2, P2Y4, P2Y6 and P2Y14. The mentioned purine receptors, especially P2Y2 and P2Y6 play a significant role in tumorigenesis and metastasis [276,277]. UDP released by the doxorubicin treated breast cancer cells, upregulates the expression as well as the enzymatic effect of MMP9 via P2Y6 mediated activation of MAPK and NF-κB signaling [278]. Such UDP-induced effect of MMP9 was found to promote metastasis of breast cancer cells in both the *in vitro* and *in vivo* models of breast cancer.

The overexpression of two important enzymes of uridine salvage pathway i.e., uridine phosphorylase and uridine-cytidine kinase in different malignancies such as oral squamous cell carcinoma [279], breast [280], thyroid [281] and lung cancers [282] is considered as an indicator of nodal metastasis and poor prognosis. Guan and coworkers [281] have investigated the involvement of uridine phosphorylase on the proliferation and metastasis of the thyroid carcinoma cells. The inhibition of uridine phosphorylase expression resulted into a significant decline in proliferation, invasion and metastasis of cancer cells. Their findings suggested a possible involvement of uridine phosphorylase in the regulation of EMT, however, a further investigation is required to validate this postulation.

### Uric Acid

The biosynthesis of uric acid (UA) from xanthine, during the adenine and guanine degradation, takes place in skeletal muscles, liver, and adipocytes. A number of clinical studies have confirmed a positive correlation between serum UA levels and obesity [283–285]. While evaluating the correlation between cancer and hyperuricemia, one must consider the fact that the higher serum UA levels in cancer patients could be consequent to the cancer itself. The extracellular release of UA that is secondary to the cancer-associated cell necrosis may account for hyperuricemia in cancer patients. However, the findings of several prospective studies have confirmed a significantly higher occurrence of cancer in individuals with higher serum UA levels, proposing hyperuricemia as a potential risk factor for developing cancer [286–288]. A serum UA level above 358 µM was reported as the prospective risk factor in harboring prostate cancers [289]. Moreover, the survival rate of cancer patients with higher serum UA levels is reported as a potential indicator of poor prognosis and diminished survival rate in a number of malignancies such as renal [290], colorectal [291] gastric cancers [292] and osteosarcoma [293]. Now we focus on the possible underlying mechanism that could be attributed to the cancer-supportive nature of UA.

UA exhibits a dual redox behavior with its potential anti-oxidant effect in the extracellular fluid [294] and an oxidant nature in the intracellular environment [295,296]. It activates NADPH mediated oxidative stress in a number of cells such as the adipocytes [297], endothelial cells [298], vascular smooth muscles cells [299], proximal tubular cells [300] and hepatocytes [301]. It is important to mention here that the generation of reactive oxygen species (ROS) during the intracellular oxidative stress is one of the non-apoptotic cell-death mechanisms that has been observed in the extracellular matrix-detached cells [271]. The cancer cells neutralize ROS by upgrading Nrf2 expression to survive and metastasize [302]. By activating NADPH oxidase (NOX) activity, UA induces oxidative stress that might be oppressive for cancer cell survival and metastasis. Moreover, UA was initially hypothesized as a potential anti-cancer agent amid its remarkable extracellular antioxidant properties [303]. However, the extensive research in the following years presented an entirely different picture where UA appeared to be associated with higher cancer risk, poor prognosis and aggressive metastasis as discussed above.

An elevated level of xanthine oxidoreductase (XOR) helps depleting the intracellular xanthine and hypoxanthine levels by converting these metabolites into UA along with ROS generation. A higher expression of XOR is hence believed to suppress tumorigenesis as it boosts the degradation of purines, thus making them unavailable for nucleic acid synthesis. That is why, a downregulation of XOR is reported in a number of aggressive malignancies [304–306]. By downregulating XOR, the cancer cells maintain a pool of purines that are utilized into the replication of genetic material in these rapidly dividing cells. The circulating UA enters the cells through special transporter proteins such as urate transporter 1 (Urat1) and glucose transporter 9 (GLUT9). As the intracellular UA levels rise, the activity of XOR is attenuated. The decline in XOR activity precipitates into a higher intracellular purine level that helps the cancer cells to proliferate at a higher rate. Further, hyperuricemia may downregulate the expression of adiponectin via attenuating peroxisome proliferator-activated receptor gamma (PPARγ) in adipocytes [307]. Being a potential inhibitor of cell proliferation, adiponectin serves as an endogenous anticancer marker and its downregulation by UA may facilitate proliferation and metastasis of cancer cells as discussed above. Moreover, the downregulation of XOR in cancer cells is associated with an increased expression of two important factors, the inhibitor of differentiation 1 (ID1) and cyclooxygenase 2 (COX-2) [308,309]. Such upregulation of ID1 and COX-2 is associated with higher migratory properties, metabolic adaptations for survival in drastic conditions, enhanced tumorigenic angiogenesis and metastasis [309–312]. Hence, the hyperuricemia may potentiate invasiveness and metastasis in cancer cells by increasing the ID1 and COX-2 expression via XOR downregulation. Besides, a higher expression of COX-2 and other inflammatory mediators may turn into a hyperuricemia induced chronic inflammatory condition which further promotes the survival and metastasis of cancer cells [313,314].

Besides the downregulation of XOR, the activation of NOX in cancer cells may also upregulate the expression of COX-2 [315]. Being an activator of NOX, UA may upregulate COX-2 expression by NOX pathway as well. Further, UA has been recently reported to increase the expression of proinflammatory cytokine TNF-α and toll like receptor 4 (TLR4) in macrophages [316]. It is important to mention here that the activated tumor-associated macrophages may promote cancer progression by upregulating COX-2 in cancer cells via IL-1β auto amplification [315]. Such auto amplification of IL-1β is found to involve NLRP3 inflammasome activation in cancer-associated macrophages [317]. Once activated, these NLRP3 inflammasomes may drastically promote cancer angiogenesis, endothelial cell proliferation, and immunosuppressive cell activation via releasing IL-1β and IL-18 [318,319]. Importantly, UA is reported to contribute in hepatic steatosis by activating NLRP3 inflammasomes in hepatocytes [296]. Thenceforth, it can be anticipated that UA may exhibit similar kind of inflammasome activation in cancer-associated immune cells to spur tumor invasion and metastasis. Further experiments are required however to attest such claim.

## Conclusion

**FIGURE 4:**
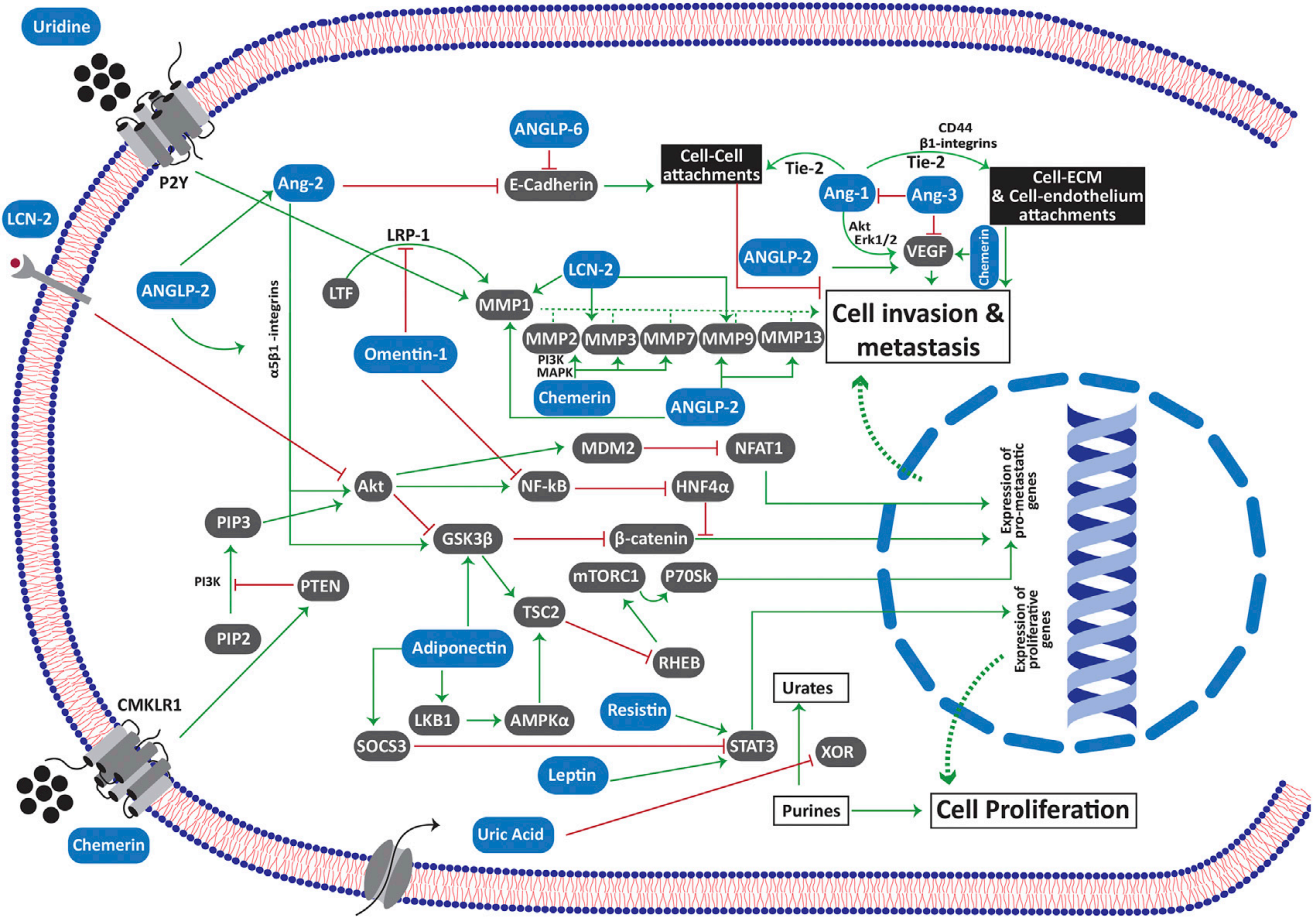
Mediation of different cell signaling pathways by adipokines in cancer cell. Abbreviations: Akt protein kinase B; ANG angiopoietin; ANGPL angiopoietin like protein; CMKLR chemerin like receptor; ERK extracellular-signal-related kinase; GSK3 glycogen synthase kinase 3; HNF hepatic nuclear factor; LCN2 lipocalin 2; LKB liver kinase B; LRP low intensity lipoprotein receptor related protein; :LTF lactotransferrin; MDM2 mouse double minute 2 homolog; MMP matrix metalloproteinase; NFAT nuclear factor of activated T cells; NF-κB nuclear factor kappa B; PIP2 phosphatidylinositol 4,5-bisphosphate; PIP3 phosphatidylinositol 3,4,5-triphosphate; PTEN phosphatase and tensin homolog; RHEB Ras homolog enriched in brain; SOCS3 suppressor of cytokine signaling 3; STAT3 signal transducer and activator of transcription 3; TSC tuberous sclerosis complex; VEGF vascular endothelial growth factor; XOR xanthine oxidoreductase.

The correlation between obesity and cancer is well-established. However, the obesity mediators contribute to the tumor growth and metastasis via extensive cell signaling pathways that requires a comprehensive review. A general conclusion of the above discussion is concisely presented in Figure 4. The adipokines regulate the proliferation and metastasis of cancer cells through complicated and interrelated pathways. Despite of their well-documented role in the disease progression, the underlying mechanisms exploited by these adipokines remain largely skeptical. For some of the adipokines, such as Ang 1, chemerin and LCN2, the consideration of their role as an anti-cancer or pro-cancer stands debatable because of their dubious role in activating both the cancer suppressive and cancer promoting signaling (please see the supplementary material). Moreover, a given adipokine may provoke proliferation and metastasis in one type of disease and may impede cancer progression in another type of cancer [209]. A number of adipokines potentiate the belligerence of the disease by promoting cell proliferation, metastasis and abating the immune checkpoints. These cytokines include leptin, resistin and ANGLPs. Contrarily, omentin and adiponectin exhibit an inhibitory effect on cancer cells by impeding cell proliferation, metastasis and inducing apoptosis. Such protective role of these anti-cancer adipokines is attested by the fact that the expression of these mediators is downregulated in aggressive cancers. Most of the adipokines promote the spread of cancer cells via upregulating the angiogenic as well as the EMT markers. Some of these markers exhibit strong immunosuppressive effect that helps the cancer cells to escape the immune checkpoints. It is important to mention that the scarcity of either of the three major external factors i.e., the nutrients, growth factors and growing space stops the entry of a cell into the cell cycle at the late G1 phase. This is the major regulatory check point that keeps the normal cells in quiescence under the unfavorable drastic conditions. Contrarily, the cancer cells escape this checkpoint to survive under the extremely harsh conditions. The adipokines provoke such tumorigenic transition by upregulating the expression of Myc, hTERT, E2F1, CycD and other proliferative genes. The adipokine mediated tumorigenic shift of the cellular microenvironment greatly affects the survival rate, remission and therapeutic success. Since the adipokine levels greatly vary among individuals, a consideration of patient adipokine profile should not be ignored while predicting the overall disease progression.

## References

[1] Madsen MS Siersbæk R Boergesen M Nielsen R Mandrup S. Peroxisome Proliferator-Activated Receptor γ and C/EBPα Synergistically Activate Key Metabolic Adipocyte Genes by Assisted Loading. Mol Cel Biol (2014) 34(6):939–54. 10.1128/mcb.01344-13 PMC3958030 24379442

[2] Shamsi F Xue R Huang TL Lundh M Liu Y Leiria L FGF6 and FGF9 Regulate UCP1 Expression Independent of Brown Adipogenesis. Nat Commun (2020) 11(1):1421. 10.1038/s41467-020-15055-9 32184391 PMC7078224

[3] Leitner BP Huang S Brychta RJ Duckworth CJ Baskin AS McGehee S Mapping of Human Brown Adipose Tissue in Lean and Obese Young Men. Proc Natl Acad Sci USA (2017) 114(32):8649–54. 10.1073/pnas.1705287114 28739898 PMC5559032

[4] Wu J Boström P Sparks LM Ye L Choi JH Giang A-H Beige Adipocytes Are a Distinct Type of Thermogenic Fat Cell in Mouse and Human. Cell (2012) 150(2):366–76. 10.1016/j.cell.2012.05.016 22796012 PMC3402601

[5] Garcia RA Roemmich JN Claycombe KJ. Evaluation of Markers of Beige Adipocytes in white Adipose Tissue of the Mouse. Nutr Metab (2016) 13:24. 10.1186/s12986-016-0081-2 PMC4797138 26997968

[6] Okla M Ha J-H Temel RE Chung S. BMP7 Drives Human Adipogenic Stem Cells into Metabolically Active Beige Adipocytes. Lipids (2015) 50(2):111–20. 10.1007/s11745-014-3981-9 25534037 PMC4306630

[7] Liu P Huang S Ling S Xu S Wang F Zhang W Foxp1 Controls Brown/beige Adipocyte Differentiation and Thermogenesis through Regulating β3-AR Desensitization. Nat Commun (2019) 10(1):5070. 10.1038/s41467-019-12988-8 31699980 PMC6838312

[8] Organization WH. *Obesity And Overweight*. News Room: Fact Sheets 2021. Available from: https://www.who.int/news-room/fact-sheets/detail/obesity-and-overweight .

[9] Reeves GK Pirie K Beral V Green J Spencer E Bull D. Cancer Incidence and Mortality in Relation to Body Mass index in the Million Women Study: Cohort Study. Bmj (2007) 335(7630):1134. 10.1136/bmj.39367.495995.ae 17986716 PMC2099519

[10] Arnold M Pandeya N Byrnes G Renehan AG Stevens GA Ezzati M Global burden of Cancer Attributable to High Body-Mass index in 2012: a Population-Based Study. Lancet Oncol (2015) 16(1):36–46. 10.1016/s1470-2045(14)71123-4 25467404 PMC4314462

[11] Odutola MK Olukomogbon T Igbinoba F Otu TI Ezeome E Hassan R Cancers Attributable to Overweight and Obesity from 2012 to 2014 in Nigeria: A Population-Based Cancer Registry Study. Front Oncol (2019) 9:460. 10.3389/fonc.2019.00460 31245287 PMC6579889

[12] de Gonzalez AB Sweetland S Spencer E. A Meta-Analysis of Obesity and the Risk of Pancreatic Cancer. Br J Cancer (2003) 89(3):519–23. 10.1038/sj.bjc.6601140 12888824 PMC2394383

[13] Holt DJ Grainger DW. Senescence and Quiescence Induced Compromised Function in Cultured Macrophages. Biomaterials (2012) 33(30):7497–507. 10.1016/j.biomaterials.2012.06.099 22809642 PMC3422570

[14] Liu H Adler AS Segal E Chang HY. A Transcriptional Program Mediating Entry into Cellular Quiescence. Plos Genet (2007) 3(6):e91. 10.1371/journal.pgen.0030091 17559306 PMC1904355

[15] Seyfried TN Huysentruyt LC. On the Origin of Cancer Metastasis. Crit Rev oncogenesis (2013) 18(1-2):43–73. 10.1615/critrevoncog.v18.i1-2.40 23237552 PMC3597235

[16] Piva de Freitas P Senna CG Tabai M Chone CT Altemani A. Metastatic Basal Cell Carcinoma: A Rare Manifestation of a Common Disease. Case Rep Med (2017) 2017:8929745. 10.1155/2017/8929745 29279714 PMC5723960

[17] García Franco CE Torre W Tamura A Guillén-Grima F San-Julian M Martin-Algarra S Long-term Results after Resection for Bone Sarcoma Pulmonary Metastases. Eur J Cardiothorac Surg (2010) 37(5):1205–8. 10.1016/j.ejcts.2009.11.026 20022760

[18] Cho YK Lee J Kim HS Park JY Lee WJ Kim YJ Metabolic Health Is a Determining Factor for Incident Colorectal Cancer in the Obese Population: A Nationwide Population‐based Cohort Study. Cancer Med (2021) 10(1):220–9. 10.1002/cam4.3607 33216467 PMC7826459

[19] James DB Mary KG Christian W. TNM Classification of Malignant Tumours. Wiley-Blackwell (2016).

[20] McAlpine J Leon-Castillo A Bosse T. The Rise of a Novel Classification System for Endometrial Carcinoma; Integration of Molecular Subclasses. J Pathol (2018) 244(5):538–49. 10.1002/path.5034 29344951

[21] Yanovich G Agmon H Harel M Sonnenblick A Peretz T Geiger T. Clinical Proteomics of Breast Cancer Reveals a Novel Layer of Breast Cancer Classification. Cancer Res (2018) 78(20):6001–10. 10.1158/0008-5472.CAN-18-1079 30154156 PMC6193543

[22] Ilie M Hofman V Dietel M Soria J-C Hofman P. Assessment of the PD-L1 Status by Immunohistochemistry: Challenges and Perspectives for Therapeutic Strategies in Lung Cancer Patients. Virchows Arch (2016) 468(5):511–25. 10.1007/s00428-016-1910-4 26915032

[23] Mamatjan Y Agnihotri S Goldenberg A Tonge P Mansouri S Zadeh G Molecular Signatures for Tumor Classification. J Mol Diagn (2017) 19(6):881–91. 10.1016/j.jmoldx.2017.07.008 28867603

[24] Caldefie-Chézet F Damez M de Latour M Konska G Mishellani F Fusillier C Leptin: A Proliferative Factor for Breast Cancer? Biochem Biophysical Res Commun (2005) 334(3):737–41. 10.1016/j.bbrc.2005.06.077 16009333

[25] Yang G Fan W Luo B Xu Z Wang P Tang S Circulating Resistin Levels and Risk of Colorectal Cancer: A Meta-Analysis. Biomed Res Int (2016) 2016:7367485. 10.1155/2016/7367485 27642602 PMC5013211

[26] Hu W-W Tang C-H Sun Y Lu T-T Jiang P Wu Y-M Correlation between Resistin Gene Polymorphism and Clinical Aspects of Lung Cancer. Medicine (2017) 96(52):e9485. 10.1097/md.0000000000009485 29384942 PMC6392976

[27] Diakowska D Markocka-Mączka K Szelachowski P Grabowski K. Serum Levels of Resistin, Adiponectin, and Apelin in Gastroesophageal Cancer Patients. Dis Markers (2014) 2014:619649. 10.1155/2014/619649 25049439 PMC4094727

[28] Cymbaluk-Płoska A Chudecka-Głaz A Pius-Sadowska E Sompolska-Rzechuła A Machaliński B Menkiszak J. Circulating Serum Level of Visfatin in Patients with Endometrial Cancer. Biomed Res Int (2018) 2018:8576179. 10.1155/2018/8576179 29516012 PMC5817356

[29] Vallega KA Liu N Myers JS Yu K Sang Q-XA. Elevated Resistin Gene Expression in African American Estrogen and Progesterone Receptor Negative Breast Cancer. PloS one (2016) 11(6):e0157741. 10.1371/journal.pone.0157741 27314854 PMC4912107

[30] Funcke J-B Scherer PE. Beyond Adiponectin and Leptin: Adipose Tissue-Derived Mediators of Inter-organ Communication. J Lipid Res (2019) 60–16481684. 10.1194/jlr.R094060 PMC6795086 31209153

[31] Wang H Liang X Li M Tao X Tai S Fan Z Chemokine (CCmotif) Ligand 18 Upregulates Slug Expression to Promote Stem‐cell like Features by Activating the Mammalian Target of Rapamycin Pathway in Oral Squamous Cell Carcinoma. Cancer Sci (2017) 108(8):1584–93. 10.1111/cas.13289 28574664 PMC5543498

[32] Park J Scherer PE. Leptin and Cancer: from Cancer Stem Cells to Metastasis. Endocrine-related cancer (2011) 18(4):C25–C29. 10.1530/erc-11-0163 21680729 PMC4110513

[33] Hardwick JCH Van Den Brink GR Offerhaus GJ Van Deventer SJH Peppelenbosch MP. Leptin Is a Growth Factor for Colonic Epithelial Cells. Gastroenterology (2001) 121(1):79–90. 10.1053/gast.2001.25490 11438496

[34] Attoub S Noe V Pirola L Bruyneel E Chastre E Mareel M Leptin Promotes Invasiveness of Kidney and Colonic Epithelial Cells via Phosphoinositide 3‐kinase‐, Rho‐, and Rac‐dependent Signaling Pathways. FASEB j (2000) 14(14):2329–38. 10.1096/fj.00-0162 11053255

[35] Buyse M Berlioz F Guilmeau S Tsocas A Voisin T Péranzi G PepT1-mediated Epithelial Transport of Dipeptides and Cephalexin Is Enhanced by Luminal Leptin in the Small Intestine. J Clin Invest (2001) 108(10):1483–94. 10.1172/jci13219 11714740 PMC209419

[36] Koda M Sulkowska M Kanczuga-Koda L Surmacz E Sulkowski S. Overexpression of the Obesity Hormone Leptin in Human Colorectal Cancer. J Clin Pathol (2007) 60(8):902–6. 10.1136/jcp.2006.041004 17660334 PMC1994494

[37] Gu L Wang C-D Cao C Cai L-R Li D-H Zheng Y-Z. Association of Serum Leptin with Breast Cancer: A Meta-Analysis. Medicine (2019) 98(5):e14094. 10.1097/md.0000000000014094 30702563 PMC6380739

[38] Jeong Y-J Bong J-G Park S-H Choi J-H Oh H-K. Expression of Leptin, Leptin Receptor, Adiponectin, and Adiponectin Receptor in Ductal CarcinomaIn Situand Invasive Breast Cancer. J Breast Cancer (2011) 14(2):96–103. 10.4048/jbc.2011.14.2.96 21847403 PMC3148540

[39] Harris HR Tworoger SS Hankinson SE Rosner BA Michels KB. Plasma Leptin Levels and Risk of Breast Cancer in Premenopausal Women. Cancer Prev Res (2011) 4(9):1449–56. 10.1158/1940-6207.capr-11-0125 PMC3168067 21680707

[40] Gu F Kraft P Rice M Michels KB. Leptin and Leptin Receptor Genes in Relation to Premenopausal Breast Cancer Incidence and Grade in Caucasian Women. Breast Cancer Res Treat (2012) 131(1):17–25. 10.1007/s10549-011-1778-6 21947707 PMC3627481

[41] Ferla R Bonomi M Otvos L Jr Surmacz E. Glioblastoma-derived Leptin Induces Tube Formation and Growth of Endothelial Cells: Comparison with VEGF Effects. BMC cancer (2011) 11:303. 10.1186/1471-2407-11-303 21771332 PMC3146945

[42] Wang X-J Yuan S-L Lu Q Lu Y-R Zhang J Liu Y Potential Involvement of Leptin in Carcinogenesis of Hepatocellular Carcinoma. Wjg (2004) 10(17):2478–81. 10.3748/wjg.v10.i17.2478 15300888 PMC4572145

[43] Zhou J Lei W Shen L Luo H-S Shen Z-X. Primary Study of Leptin and Human Hepatocellular Carcinoma *In Vitro* . Wjg (2008) 14(18):2900–4. 10.3748/wjg.14.2900 18473418 PMC2710735

[44] Saxena NK Sharma D Ding X Lin S Marra F Merlin D Concomitant Activation of the JAK/STAT, PI3K/AKT, and ERK Signaling Is Involved in Leptin-Mediated Promotion of Invasion and Migration of Hepatocellular Carcinoma Cells. Cancer Res (2007) 67(6):2497–507. 10.1158/0008-5472.can-06-3075 17363567 PMC2925446

[45] Ptak A Kolaczkowska E Gregoraszczuk EL. Leptin Stimulation of Cell Cycle and Inhibition of Apoptosis Gene and Protein Expression in OVCAR-3 Ovarian Cancer Cells. Endocrine (2013) 43(2):394–403. 10.1007/s12020-012-9788-7 22968658 PMC3593082

[46] Pan W Allison MB Sabatini P Rupp A Adams J Patterson C Transcriptional and Physiological Roles for STAT Proteins in Leptin Action. Mol Metab (2019) 22:121–31. 10.1016/j.molmet.2019.01.007 30718218 PMC6437596

[47] Saxena NK Vertino PM Anania FA Sharma D. Leptin-induced Growth Stimulation of Breast Cancer Cells Involves Recruitment of Histone Acetyltransferases and Mediator Complex to CYCLIN D1 Promoter via Activation of Stat3. J Biol Chem (2007) 282(18):13316–25. 10.1074/jbc.m609798200 17344214 PMC2923657

[48] Saxena NK Titus MA Ding X Floyd J Srinivasan S Sitaraman SV Leptin as a Novel Profibrogenic Cytokine in Hepatic Stellate Cells: Mitogenesis and Inhibition of Apoptosis Mediated by Extracellular Regulated Kinase (Erk) and Akt Phosphorylation. FASEB j (2004) 18(13):1612–4. 10.1096/fj.04-1847fje 15319373 PMC2924993

[49] Higurashi T Endo H Uchiyama T Uchiyama S Yamada E Ohkubo H Conditional Knockout of the Leptin Receptor in the Colonic Epithelium Revealed the Local Effects of Leptin Receptor Signaling in the Progression of Colonic Tumors in Mice. Carcin (2014) 35(9):2134–41. 10.1093/carcin/bgu135 24958593

[50] Xiong H Zhang Z-G Tian X-Q Sun D-F Liang Q-C Zhang Y-J Inhibition of JAK1, 2/STAT3 Signaling Induces Apoptosis, Cell Cycle Arrest, and Reduces Tumor Cell Invasion in Colorectal Cancer Cells. Neoplasia (2008) 10(3):287–97. 10.1593/neo.07971 18320073 PMC2259457

[51] Lin Q Lai R Chirieac LR Li C Thomazy VA Grammatikakis I Constitutive Activation of JAK3/STAT3 in Colon Carcinoma Tumors and Cell Lines. Am J Pathol (2005) 167(4):969–80. 10.1016/s0002-9440(10)61187-x 16192633 PMC1603671

[52] Nowakowska-Zajdel E Mazurek U Stachowicz M Niedworok E Fatyga E Muc-Wierzgoń M. Cellular Signal Transduction Pathways by Leptin in Colorectal Cancer Tissue: Preliminary Results. ISRN Endocrinol (2011) 2011:575397. 10.5402/2011/575397 22363883 PMC3262645

[53] Mittenbühler MJ Sprenger H-G Gruber S Wunderlich CM Kern L Brüning JC Hepatic Leptin Receptor Expression Can Partially Compensate for IL-6Rα Deficiency in DEN-Induced Hepatocellular Carcinoma. Mol Metab (2018) 17:122–33. 10.1016/j.molmet.2018.08.010 30224299 PMC6197506

[54] Zheng Q Banaszak L Fracci S Basali D Dunlap SM Hursting SD Leptin Receptor Maintains Cancer Stem-like Properties in Triple Negative Breast Cancer Cells. Endocrine-related cancer (2013) 20(6):797–808. 10.1530/erc-13-0329 24025407 PMC3843956

[55] Thiagarajan PS Zheng Q Bhagrath M Mulkearns-Hubert EE Myers MG Lathia JD STAT3 Activation by Leptin Receptor Is Essential for TNBC Stem Cell Maintenance. Endocr Relat Cancer (2017) 24(8):415–26. 10.1530/erc-16-0349 28729467 PMC5551450

[56] Aparicio T Kotelevets L Tsocas A Laigneau JP Sobhani I Chastre E Leptin Stimulates the Proliferation of Human colon Cancer Cells *In Vitro* but Does Not Promote the Growth of colon Cancer Xenografts in Nude Mice or Intestinal Tumorigenesis in ApcMin/+ Mice. Gut (2005) 54(8):1136–45. 10.1136/gut.2004.060533 15857934 PMC1774895

[57] Deo DD Rao AP Bose SS Ouhtit A Baliga SB Rao SA Differential Effects of Leptin on the Invasive Potential of Androgen-dependent and -independent Prostate Carcinoma Cells. J Biomed Biotechnol (2008) 2008:163902. 10.1155/2008/163902 18584049 PMC2435597

[58] Naviglio S Di Gesto D Sorrentino A Illiano F Sorvillo L Abbruzzese A Leptin Enhances Growth Inhibition by cAMP Elevating Agents through Apoptosis of MDA-MB-231 Breast Cancer Cells. Cancer Biol Ther (2009) 8(12):1183–90. 10.4161/cbt.8.12.8562 19662684

[59] Spina A Di Maiolo F Esposito A Sapio L Chiosi E Sorvillo L cAMP Elevation Down-Regulates β3 Integrin and Focal Adhesion Kinase and Inhibits Leptin-Induced Migration of MDA-MB-231 Breast Cancer Cells. BioResearch open access (2012) 1(6):324–32. 10.1089/biores.2012.0270 23515360 PMC3559230

[60] Catalano S Mauro L Bonofiglio D Pellegrino M Qi H Rizza P *In Vivo* and *In Vitro* Evidence that PPARγ Ligands Are Antagonists of Leptin Signaling in Breast Cancer. Am J Pathol (2011) 179(2):1030–40. 10.1016/j.ajpath.2011.04.026 21704006 PMC3157221

[61] Alshaker H Krell J Frampton AE Waxman J Blyuss O Zaikin A Leptin Induces Upregulation of Sphingosine Kinase 1 in Oestrogen Receptor-Negative Breast Cancer via Src Family Kinase-Mediated, Janus Kinase 2-independent Pathway. Breast Cancer Res : BCR (2014) 16(5):426. 10.1186/s13058-014-0426-6 25482303 PMC4303110

[62] Bjørbaek C El-Haschimi K Frantz JD Flier JS. The Role of SOCS-3 in Leptin Signaling and Leptin Resistance. J Biol Chem (1999) 274(42):30059–65. 10.1074/jbc.274.42.30059 10514492

[63] Wan J Che Y Kang N Wu W. SOCS3 Blocks HIF-1α Expression to Inhibit Proliferation and Angiogenesis of Human Small Cell Lung Cancer by Downregulating Activation of Akt, but Not STAT3. Mol Med Rep (2015) 12(1):83–92. 10.3892/mmr.2015.3368 25695729 PMC4438922

[64] Knobelspies H Zeidler J Hekerman P Bamberg-Lemper S Becker W. Mechanism of Attenuation of Leptin Signaling under Chronic Ligand Stimulation. BMC Biochem (2010) 11:2. 10.1186/1471-2091-11-2 20059770 PMC2821298

[65] Bode J Ludwig S Freitas CA Schaper F Wammers M Melmed S The MKK6/p38 Mitogen-Activated Protein Kinase Pathway Is Capable of Inducing SOCS3 Gene Expression and Inhibits IL-6-Induced Transcription. Biol Chem (2001) 382:1447–53. 10.1515/bc.2001.178 11727828

[66] Thompson KJ Lau KN Johnson S Martinie JB Iannitti DA McKillop IH Leptin Inhibits Hepatocellular Carcinoma Proliferation via P38-MAPK-dependent Signalling. Hpb (2011) 13(4):225–33. 10.1111/j.1477-2574.2010.00259.x 21418127 PMC3081622

[67] Inagaki-Ohara K Mayuzumi H Kato S Minokoshi Y Otsubo T Kawamura YI Enhancement of Leptin Receptor Signaling by SOCS3 Deficiency Induces Development of Gastric Tumors in Mice. Oncogene (2014) 33(1):74–84. 10.1038/onc.2012.540 23178499

[68] Ramani K Yang H Xia M Ara AI Mato JM Lu SC. Leptin's Mitogenic Effect in Human Liver Cancer Cells Requires Induction of Both Methionine Adenosyltransferase 2A and 2beta. Hepatology (2008) 47(2):521–31. 10.1002/hep.22064 18041713 PMC2387125

[69] Stefanou N Papanikolaou V Furukawa Y Nakamura Y Tsezou A. Leptin as a Critical Regulator of Hepatocellular Carcinoma Development through Modulation of Human Telomerase Reverse Transcriptase. BMC cancer (2010) 10:442. 10.1186/1471-2407-10-442 20723213 PMC2931493

[70] Kasiappan R Sun Y Lungchukiet P Quarni W Zhang X Bai W. Vitamin D Suppresses Leptin Stimulation of Cancer Growth through microRNA. Cancer Res (2014) 74(21):6194–204. 10.1158/0008-5472.can-14-1702 25252917 PMC4216743

[71] Fukada T Ohtani T Yoshida Y Shirogane T Nishida K Nakajima K STAT3 Orchestrates Contradictory Signals in Cytokine-Induced G1 to S Cell-Cycle Transition. EMBO J (1998) 17(22):6670–7. 10.1093/emboj/17.22.6670 9822610 PMC1171012

[72] Harbuzariu A Rampoldi A Daley-Brown DS Candelaria P Harmon TL Lipsey CC Leptin-Notch Signaling axis Is Involved in Pancreatic Cancer Progression. Oncotarget (2017) 8(5):7740–52. 10.18632/oncotarget.13946 27999190 PMC5352357

[73] Feldheim J Kessler AF Schmitt D Salvador E Monoranu CM Feldheim JJ Ribosomal Protein S27/Metallopanstimulin-1 (RPS27) in Glioma-A New Disease Biomarker? Cancers (2020) 12(5):1085. 10.3390/cancers12051085 PMC7281545 32349320

[74] Cao D Luo Y Qin S Yu M Mu Y Ye G Metallopanstimulin-1 (MPS-1) Mediates the Promotion Effect of Leptin on Colorectal Cancer through Activation of JNK/c-Jun Signaling Pathway. Cel Death Dis (2019) 10(9):655. 10.1038/s41419-019-1911-8 PMC6736844 31506433

[75] Yang Z-Y Jiang H Qu Y Wei M Yan M Zhu Z-G Metallopanstimulin-1 Regulates Invasion and Migration of Gastric Cancer Cells Partially through Integrin β4. Carcinogenesis (2013) 34(12):2851–60. 10.1093/carcin/bgt226 23803695

[76] Christopoulos PF Msaouel P Koutsilieris M. The Role of the Insulin-like Growth Factor-1 System in Breast Cancer. Mol Cancer (2015) 14:43. 10.1186/s12943-015-0291-7 25743390 PMC4335664

[77] Min DY Jung E Kim J Lee YH Shin SY. Leptin Stimulates IGF-1 Transcription by Activating AP-1 in Human Breast Cancer Cells. BMB Rep (2019) 52(6):385–90. 10.5483/bmbrep.2019.52.6.189 30293548 PMC6605521

[78] Yang W-H Chang A-C Wang S-W Wang S-J Chang Y-S Chang T-M Leptin Promotes VEGF-C Production and Induces Lymphangiogenesis by Suppressing miR-27b in Human Chondrosarcoma Cells. Scientific Rep (2016) 6(1):28647. 10.1038/srep28647 PMC4921910 27345723

[79] Carino C Olawaiye AB Cherfils S Serikawa T Lynch MP Rueda BR Leptin Regulation of Proangiogenic Molecules in Benign and Cancerous Endometrial Cells. Int J Cancer (2008) 123(12):2782–90. 10.1002/ijc.23887 18798554 PMC2892183

[80] Zhou W Guo S Gonzalez-Perez RR. Leptin Pro-angiogenic Signature in Breast Cancer Is Linked to IL-1 Signalling. Br J Cancer (2011) 104(1):128–37. 10.1038/sj.bjc.6606013 21139583 PMC3039812

[81] Gonzalez-Perez RR Xu Y Guo S Watters A Zhou W Leibovich SJ. Leptin Upregulates VEGF in Breast Cancer via Canonic and Non-canonical Signalling Pathways and NFκB/HIF-1α Activation. Cell Signal (2010) 22(9):1350–62. 10.1016/j.cellsig.2010.05.003 20466060 PMC2928711

[82] Guo S Gonzalez-Perez RR. Notch, IL-1 and Leptin Crosstalk Outcome (NILCO) Is Critical for Leptin-Induced Proliferation, Migration and VEGF/VEGFR-2 Expression in Breast Cancer. PloS one (2011) 6(6):e21467. 10.1371/journal.pone.0021467 21731759 PMC3121792

[83] Zhao X Dong Y Zhang J Li D Hu G Yao J Leptin Changes Differentiation Fate and Induces Senescence in Chondrogenic Progenitor Cells. Cell Death Dis (2016) 7(4):e2188. 10.1038/cddis.2016.68 27077804 PMC4855655

[84] He J-Y Wei X-H Li S-J Liu Y Hu H-L Li Z-Z Adipocyte-derived IL-6 and Leptin Promote Breast Cancer Metastasis via Upregulation of Lysyl Hydroxylase-2 Expression. Cell Commun signaling : CCS (2018) 16(1):100. 10.1186/s12964-018-0309-z 30563531 PMC6299564

[85] Jaffe T Schwartz B. Leptin Promotes Motility and Invasiveness in Human colon Cancer Cells by Activating Multiple Signal-Transduction Pathways. Int J Cancer (2008) 123(11):2543–56. 10.1002/ijc.23821 18767036

[86] Strong AL Strong TA Rhodes LV Semon JA Zhang X Shi Z Obesity Associated Alterations in the Biology of Adipose Stem Cells Mediate Enhanced Tumorigenesis by Estrogen Dependent Pathways. Breast Cancer Res (2013) 15(5):R102. 10.1186/bcr3569 24176089 PMC3978929

[87] Yan D Avtanski D Saxena NK Sharma D. Leptin-induced Epithelial-Mesenchymal Transition in Breast Cancer Cells Requires β-Catenin Activation via Akt/GSK3- and MTA1/Wnt1 Protein-dependent Pathways. J Biol Chem (2012) 287(11):8598–612. 10.1074/jbc.m111.322800 22270359 PMC3318705

[88] Liang X Wang S Wang X Zhang L Zhao H Zhang L. Leptin Promotes the Growth of Breast Cancer by Upregulating the Wnt/β-Catenin Pathway. Exp Ther Med (2018) 16(2):767–71. 10.3892/etm.2018.6212 30116331 PMC6090264

[89] Haque I Ghosh A Acup S Banerjee S Dhar K Ray A Leptin-induced ER-α-Positive Breast Cancer Cell Viability and Migration Is Mediated by Suppressing CCN5-Signaling via Activating JAK/AKT/STAT-pathway. BMC cancer (2018) 18(1):99. 10.1186/s12885-018-3993-6 29370782 PMC5785848

[90] Knight BB Oprea-Ilies GM Nagalingam A Yang L Cohen C Saxena NK Survivin Upregulation, Dependent on Leptin-EGFR-Notch1 axis, Is Essential for Leptin-Induced Migration of Breast Carcinoma Cells. Endocrine-related cancer (2011) 18(4):413–28. 10.1530/erc-11-0075 21555376 PMC3361735

[91] Battle M Gillespie C Quarshie A Lanier V Harmon T Wilson K Obesity Induced a Leptin-Notch Signaling axis in Breast Cancer. Int J Cancer (2014) 134(7):1605–16. 10.1002/ijc.28496 24114531 PMC3947126

[92] Mociño-Rodríguez MD Santillán-Benítez JG Dozal-Domínguez DS Hernández-Navarro MD Flores-Merino MV Sandoval-Cabrera A Expression of AdipoR1 and AdipoR2 Receptors as Leptin-Breast Cancer Regulation Mechanisms. Dis Markers (2017) 2017:4862016. 10.1155/2017/4862016 29311755 PMC5605926

[93] Wu D-C Zhang M-F Su S-G Fang H-Y Wang X-H He D HEY2, a Target of miR-137, Indicates Poor Outcomes and Promotes Cell Proliferation and Migration in Hepatocellular Carcinoma. Oncotarget (2016) 7(25):38052–63. 10.18632/oncotarget.9343 27191260 PMC5122371

[94] Tanaka T Terai Y Kogata Y Ashihara K Maeda K Fujiwara S CD24 Expression as a Marker for Predicting Clinical Outcome and Invasive Activity in Uterine Cervical Cancer. Oncol Rep (2015) 34(5):2282–8. 10.3892/or.2015.4257 26351781 PMC4583540

[95] Xiaoping L Xiaowei Z Leizhen Z Weijian G. Expression and Significance of CD44 and P-AKT in Pancreatic Head Cancer. World J Surg Oncol (2015) 13:334. 10.1186/s12957-015-0746-8 26666511 PMC4678728

[96] Tsai CF Chen JH Wu CT Chang PC Wang SL Yeh WL. Induction of Osteoclast-like Cell Formation by Leptin-Induced Soluble Intercellular Adhesion Molecule Secreted from Cancer Cells. Ther Adv Med Oncol (2019) 11:1758835919846806. 10.1177/1758835919846806 31205504 PMC6535721

[97] Dong Z Fu S Xu X Yang Y Du L Li W Leptin-mediated Regulation of ICAM-1 Is Rho/ROCK Dependent and Enhances Gastric Cancer Cell Migration. Br J Cancer (2014) 110(7):1801–10. 10.1038/bjc.2014.70 24548863 PMC3974087

[98] Strong AL Ohlstein JF Biagas BA Rhodes LV Pei DT Tucker HA Leptin Produced by Obese Adipose Stromal/stem Cells Enhances Proliferation and Metastasis of Estrogen Receptor Positive Breast Cancers. Breast Cancer Res (2015) 17(1):112. 10.1186/s13058-015-0622-z 26286584 PMC4541745

[99] Sabol RA Bowles AC Côté A Wise R O'Donnell B Matossian MD Leptin Produced by Obesity-Altered Adipose Stem Cells Promotes Metastasis but Not Tumorigenesis of Triple-Negative Breast Cancer in Orthotopic Xenograft and Patient-Derived Xenograft Models. Breast Cancer Res : BCR (2019) 21(1):67. 10.1186/s13058-019-1153-9 31118047 PMC6530039

[100] Wei L Li K Pang X Guo B Su M Huang Y Leptin Promotes Epithelial-Mesenchymal Transition of Breast Cancer via the Upregulation of Pyruvate Kinase M2. J Exp Clin Cancer Res : CR (2016) 35(1):166. 10.1186/s13046-016-0446-4 27769315 PMC5073421

[101] Wang L Tang C Cao H Li K Pang X Zhong L Activation of IL-8 via PI3K/Akt-dependent Pathway Is Involved in Leptin-Mediated Epithelial-Mesenchymal Transition in Human Breast Cancer Cells. Cancer Biol Ther (2015) 16(8):1220–30. 10.1080/15384047.2015.1056409 26121010 PMC4622725

[102] Xu M Cao FL Li N Gao X Su X Jiang X. Leptin Induces Epithelial-To-Mesenchymal Transition via Activation of the ERK Signaling Pathway in Lung Cancer Cells. Oncol Lett (2018) 16(4):4782–8. 10.3892/ol.2018.9230 30250542 PMC6144616

[103] Fazolini NP Cruz AL Werneck MB Viola JP Maya-Monteiro CM Bozza PT. Leptin Activation of mTOR Pathway in Intestinal Epithelial Cell Triggers Lipid Droplet Formation, Cytokine Production and Increased Cell Proliferation. Cell Cycle (2015) 14(16):2667–76. 10.1080/15384101.2015.1041684 26017929 PMC4614828

[104] Lee S-M Choi H-J Oh C-H Oh J-W Han J-S. Leptin Increases TNF-α Expression and Production through Phospholipase D1 in Raw 264.7 Cells. PloS one (2014) 9(7):e102373. 10.1371/journal.pone.0102373 25047119 PMC4105621

[105] Wu D Cheng J Sun G Wu S Li M Gao Z p70S6K Promotes IL-6-induced Epithelial-Mesenchymal Transition and Metastasis of Head and Neck Squamous Cell Carcinoma. Oncotarget (2016) 7(24):36539–50. 10.18632/oncotarget.9282 27174914 PMC5095019

[106] Hao Y Baker D Ten Dijke P. TGF-β-Mediated Epithelial-Mesenchymal Transition and Cancer Metastasis. Ijms (2019) 20(11):2767. 10.3390/ijms20112767 PMC6600375 31195692

[107] Xing S Yu W Zhang X Luo Y Lei Z Huang D Isoviolanthin Extracted from Dendrobium Officinale Reverses TGF-Β1-Mediated Epithelial-Mesenchymal Transition in Hepatocellular Carcinoma Cells via Deactivating the TGF-β/Smad and PI3K/Akt/mTOR Signaling Pathways. Ijms (2018) 19(6):1556. 10.3390/ijms19061556 PMC6032198 29882900

[108] Ogunwobi OO Wang T Zhang L Liu C. Cyclooxygenase-2 and Akt Mediate Multiple Growth-Factor-Induced Epithelial-Mesenchymal Transition in Human Hepatocellular Carcinoma. J Gastroenterol Hepatol (2012) 27(3):566–78. 10.1111/j.1440-1746.2011.06980.x 22097969 PMC3288221

[109] Bhat AA Ahmad R Uppada SB Singh AB Dhawan P. Claudin-1 Promotes TNF-α-Induced Epithelial-Mesenchymal Transition and Migration in Colorectal Adenocarcinoma Cells. Exp Cel Res (2016) 349(1):119–27. 10.1016/j.yexcr.2016.10.005 PMC6166648 27742576

[110] Wang H Wang H-S Zhou B-H Li C-L Zhang F Wang X-F Epithelial-mesenchymal Transition (EMT) Induced by TNF-α Requires AKT/GSK-3β-mediated Stabilization of Snail in Colorectal Cancer. PloS one (2013) 8(2):e56664. 10.1371/journal.pone.0056664 23431386 PMC3576347

[111] Valle A Sastre-Serra J Pol C Miró AM Oliver J Roca P. Proteomic Analysis of MCF-7 Breast Cancer Cell Line Exposed to Leptin. Anal Cell Pathol (2011) 34(3):147–57. 10.1155/2011/405253 PMC4605800 21673435

[112] Katsogiannou M Andrieu C Rocchi P. Heat Shock Protein 27 Phosphorylation State Is Associated with Cancer Progression. Front Genet (2014) 5:346. 10.3389/fgene.2014.00346 25339975 PMC4186339

[113] Mendonsa AM Chalfant MC Gorden LD VanSaun MN. Modulation of the Leptin Receptor Mediates Tumor Growth and Migration of Pancreatic Cancer Cells. PloS one (2015) 10(4):e0126686. 10.1371/journal.pone.0126686 25919692 PMC4412670

[114] Giordano C Vizza D Panza S Barone I Bonofiglio D Lanzino M Leptin Increases HER2 Protein Levels through a STAT3-Mediated Up-Regulation of Hsp90 in Breast Cancer Cells. Mol Oncol (2013) 7(3):379–91. 10.1016/j.molonc.2012.11.002 23228483 PMC5528468

[115] Lipsey CC Harbuzariu A Robey RW Huff LM Gottesman MM Gonzalez-Perez RR. Leptin Signaling Affects Survival and Chemoresistance of Estrogen Receptor Negative Breast Cancer. Ijms (2020) 21(11):3794. 10.3390/ijms21113794 PMC7311967 32471192

[116] Fiedor E Zajda K Gregoraszczuk EL. Leptin Receptor Antagonists' Action on HDAC Expression Eliminating the Negative Effects of Leptin in Ovarian Cancer. Cancer Genomics Proteomics (2018) 15(4):329–36. 10.21873/cgp.20091 29976638 PMC6070714

[117] Pachynski RK Wang P Salazar N Zheng Y Nease L Rosalez J Chemerin Suppresses Breast Cancer Growth by Recruiting Immune Effector Cells into the Tumor Microenvironment. Front Immunol (2019) 10:983. 10.3389/fimmu.2019.00983 31139180 PMC6518384

[118] Harbuzariu A Gonzalez-Perez RR. Leptin-Notch axis Impairs 5-fluorouracil Effects on Pancreatic Cancer. Oncotarget (2018) 9(26):18239–53. 10.18632/oncotarget.24435 29719602 PMC5915069

[119] Osaki M Okada F. Exosomes and Their Role in Cancer Progression. Yonago Acta Med (2019) 62(2):182–90. 10.33160/yam.2019.06.002 31320822 PMC6584259

[120] Giordano C Gelsomino L Barone I Panza S Augimeri G Bonofiglio D Leptin Modulates Exosome Biogenesis in Breast Cancer Cells: An Additional Mechanism in Cell-To-Cell Communication. Jcm (2019) 8(7):1027. 10.3390/jcm8071027 PMC6678227 31336913

[121] Raut PK Choi DY Kim SH Hong JT Kwon TK Jeong JH Estrogen Receptor Signaling Mediates Leptin-Induced Growth of Breast Cancer Cells via Autophagy Induction. Oncotarget (2017) 8(65):109417–35. 10.18632/oncotarget.22684 29312618 PMC5752531

[122] Nepal S Kim MJ Hong JT Kim SH Sohn D-H Lee SH Autophagy Induction by Leptin Contributes to Suppression of Apoptosis in Cancer Cells and Xenograft Model: Involvement of p53/FoxO3A axis. Oncotarget (2015) 6(9):7166–81. 10.18632/oncotarget.3347 25704884 PMC4466676

[123] Shrestha M Park P-H. p53 Signaling Is Involved in Leptin-Induced Growth of Hepatic and Breast Cancer Cells. Korean J Physiol Pharmacol (2016) 20(5):487–98. 10.4196/kjpp.2016.20.5.487 27610035 PMC5014995

[124] Yehuda-Shnaidman E Nimri L Tarnovscki T Kirshtein B Rudich A Schwartz B. Secreted Human Adipose Leptin Decreases Mitochondrial Respiration in HCT116 colon Cancer Cells. PloS one (2013) 8(9):e74843. 10.1371/journal.pone.0074843 24073224 PMC3779244

[125] Park J Kusminski CM Chua SC Scherer PE. Leptin Receptor Signaling Supports Cancer Cell Metabolism through Suppression of Mitochondrial Respiration *In Vivo* . Am J Pathol (2010) 177(6):3133–44. 10.2353/ajpath.2010.100595 21056997 PMC2993284

[126] Wang C-Q Wang Y Huang B-F Tang C-H Du Z Zeng Y High Expression of Both Resistin and Fascin-1 Predicts a Poor Prognosis in Patients with Colorectal Cancer. Biomed Research International (2020) 2020:8753175. 10.1155/2020/8753175 32420377 PMC7201636

[127] Mihajlovic M Ninic A Sopic M Miljkovic M Stefanovic A Vekic J Association Among Resistin, Adenylate Cyclase-Associated Protein 1 and High-Density Lipoprotein Cholesterol in Patients with Colorectal Cancer: a Multi-Marker Approach, as a Hallmark of Innovative Predictive, Preventive, and Personalized Medicine. EPMA J (2019) 10(3):307–16. 10.1007/s13167-019-00178-x 31462946 PMC6695467

[128] Avtanski D Garcia A Caraballo B Thangeswaran P Marin S Bianco J Resistin Induces Breast Cancer Cells Epithelial to Mesenchymal Transition (EMT) and Stemness through Both Adenylyl Cyclase-Associated Protein 1 (CAP1)-dependent and CAP1-independent Mechanisms. Cytokine (2019) 120:155–64. 10.1016/j.cyto.2019.04.016 31085453

[129] Weber D Satlof L Lavi A Bahl K Kaiser M Chen K SAT-335 Resistin Induces Epithelial to Mesenchymal Transition (EMT) in Breast Cancer Cells through Activation of AXL Tyrosine Kinase Receptor. J Endocr Soc (2019) 3(Suppl. 1):SAT-335. 10.1210/js.2019-sat-335

[130] Qiu L Zhang G-F Yu L Wang H-Y Jia X-J Wang T-J. Novel Oncogenic and Chemoresistance-Inducing Functions of Resistin in Ovarian Cancer Cells Require miRNAs-Mediated Induction of Epithelial-To-Mesenchymal Transition. Scientific Rep (2018) 8(1):12522. 10.1038/s41598-018-30978-6 PMC6104088 30131543

[131] Yang C-C Chang S-F Chao J-K Lai Y-L Chang W-E Hsu W-H Activation of AMP-Activated Protein Kinase Attenuates Hepatocellular Carcinoma Cell Adhesion Stimulated by Adipokine Resistin. BMC cancer (2014) 14:112. 10.1186/1471-2407-14-112 24555415 PMC3936704

[132] Huang W-S Yang J-T Lu C-C Chang S-F Chen C-N Su Y-P Fulvic Acid Attenuates Resistin-Induced Adhesion of HCT-116 Colorectal Cancer Cells to Endothelial Cells. Ijms (2015) 16(12):29370–82. 10.3390/ijms161226174 26690142 PMC4691117

[133] Hsieh Y-Y Shen C-H Huang W-S Chin C-C Kuo Y-H Hsieh M Resistin-induced Stromal Cell-Derived Factor-1 Expression through Toll-like Receptor 4 and Activation of P38 MAPK/NFκB Signaling Pathway in Gastric Cancer Cells. J Biomed Sci (2014) 21(1):59. 10.1186/1423-0127-21-59 24929539 PMC4089564

[134] Deshmukh SK Srivastava SK Zubair H Bhardwaj A Tyagi N Al-Ghadhban A Resistin Potentiates Chemoresistance and Stemness of Breast Cancer Cells: Implications for Racially Disparate Therapeutic Outcomes. Cancer Lett (2017) 396:21–9. 10.1016/j.canlet.2017.03.010 28302531 PMC5437742

[135] Mohammadi M Hedayati M Zarghami N Ghaemmaghami S. RESISTIN EFFECT ON TELOMERASE GENE EXPRESSION IN GASTRIC CANCER CELL LINE AGS. Acta Endo (Buc) (2016) 12(2):145–9. 10.4183/aeb.2016.145 PMC6535295 31149079

[136] Malvi P Chaube B Singh SV Mohammad N Vijayakumar MV Singh S Elevated Circulatory Levels of Leptin and Resistin Impair Therapeutic Efficacy of Dacarbazine in Melanoma under Obese State. Cancer Metab (2018) 6(1):2. 10.1186/s40170-018-0176-5 29568521 PMC5859707

[137] Liu Z Shi A Song D Han B Zhang Z Ma L Resistin Confers Resistance to Doxorubicin-Induced Apoptosis in Human Breast Cancer Cells through Autophagy Induction. Am J Cancer Res (2017) 7(3):574–83. 28401013 PMC5385645

[138] Kim JG Kim EO Jeong BR Min YJ Park JW Kim ES Visfatin Stimulates Proliferation of MCF-7 Human Breast Cancer Cells. Mol Cell (2010) 30(4):341–5. 10.1007/s10059-010-0124-x 20848232

[139] Buldak RJ Buldak L Polaniak R Kukla M Birkner E Kubina R Visfatin Affects Redox Adaptative Responses and Proliferation in Me45 Human Malignant Melanoma Cells: an *In Vitro* Study. Oncol Rep (2013) 29(2):771–8. 23232726 10.3892/or.2012.2175

[140] Zhao H Tang W Chen X Wang S Wang X Xu H The NAMPT/E2F2/SIRT1 axis Promotes Proliferation and Inhibits P53-dependent Apoptosis in Human Melanoma Cells. Biochem Biophysical Res Commun (2017) 493(1):77–84. 10.1016/j.bbrc.2017.09.071 28919418

[141] Adya R Tan BK Punn A Chen J Randeva HS. Visfatin Induces Human Endothelial VEGF and MMP-2/9 Production via MAPK and PI3K/Akt Signalling Pathways: Novel Insights into Visfatin-Induced Angiogenesis. Cardiovasc Res (2008) 78(2):356–65. 10.1093/cvr/cvm111 18093986

[142] Wang P Xu T-Y Guan Y-F Su D-F Fan G-R Miao C-Y. Perivascular Adipose Tissue-Derived Visfatin Is a Vascular Smooth Muscle Cell Growth Factor: Role of Nicotinamide Mononucleotide. Cardiovasc Res (2009) 81(2):370–80. 10.1093/cvr/cvn288 18952695

[143] Kim S-R Bae S-K Choi K-S Park S-Y Jun HO Lee J-Y Visfatin Promotes Angiogenesis by Activation of Extracellular Signal-Regulated Kinase 1/2. Biochem Biophysical Res Commun (2007) 357(1):150–6. 10.1016/j.bbrc.2007.03.105 17408594

[144] Liu P Li H Cepeda J Xia Y Kempf JA Ye H Regulation of Inflammatory Cytokine Expression in Pulmonary Epithelial Cells by Pre-B-cell colony-enhancing Factor via a Nonenzymatic and AP-1-dependent Mechanism. J Biol Chem (2009) 284(40):27344–51. 10.1074/jbc.m109.002519 19654329 PMC2785662

[145] Nowell MA Richards PJ Fielding CA Ognjanovic S Topley N Williams AS Regulation of Pre-B Cell colony-enhancing Factor by STAT-3-dependent Interleukin-6trans-Signaling: Implications in the Pathogenesis of Rheumatoid Arthritis. Arthritis Rheum (2006) 54(7):2084–95. 10.1002/art.21942 16802343

[146] Park H-J Kim S-R Kim SS Wee H-J Bae M-K Ryu MH Visfatin Promotes Cell and Tumor Growth by Upregulating Notch1 in Breast Cancer. Oncotarget (2014) 5(13):5087–99. 10.18632/oncotarget.2086 24970818 PMC4148124

[147] Lu G-W Wang Q-J Xia M-M Qian J. Elevated Plasma Visfatin Levels Correlate with Poor Prognosis of Gastric Cancer Patients. Peptides (2014) 58:60–4. 10.1016/j.peptides.2014.05.016 24911837

[148] Yang J Zhang K Song H Wu M Li J Yong Z Visfatin Is Involved in Promotion of Colorectal Carcinoma Malignancy through an Inducing EMT Mechanism. Oncotarget (2016) 7(22):32306–17. 10.18632/oncotarget.8615 27058759 PMC5078014

[149] Liu T Miao Z Jiang J Yuan S Fang W Li B Visfatin Mediates SCLC Cells Migration across Brain Endothelial Cells through Upregulation of CCL2. Int J Mol Sci (2015) 16(5):11439–51. 10.3390/ijms160511439 25993304 PMC4463709

[150] Lim SY Yuzhalin AE Gordon-Weeks AN Muschel RJ. Targeting the CCL2-CCR2 Signaling axis in Cancer Metastasis. Oncotarget (2016) 7(19):28697–710. 10.18632/oncotarget.7376 26885690 PMC5053756

[151] Wang G-J Shen N-J Cheng L Yehan Fang F Huang H Li K-H. Visfatin Triggers the *In Vitro* Migration of Osteosarcoma Cells via Activation of NF-κB/IL-6 Signals. Eur J Pharmacol (2016) 791:322–30. 10.1016/j.ejphar.2016.08.029 27568842

[152] Cao Z Liang N Yang H Li S. Visfatin Mediates Doxorubicin Resistance in Human Non-small-cell Lung Cancer via Akt-Mediated Up-Regulation of ABCC1. Cel Prolif (2017) 50(5):e12366. 10.1111/cpr.12366 PMC6529115 28762597

[153] Kay-Pong Y Chi LA-Y Samuel CM. Interactions of Omentin and Lactotransferrin in the Progression of Metastatic Ovarian Cancer. FASEB J (2019) 33(1_Suppl. ment):704.5.

[154] Li D Zhao X Xiao Y Mei H Pu J Xiang X Intelectin 1 Suppresses Tumor Progression and Is Associated with Improved Survival in Gastric Cancer. Oncotarget (2015) 6(18):16168–82. 10.18632/oncotarget.3753 25965823 PMC4599263

[155] Yeung CLA Co NN Onstad M Yeung T-L Leung CS Schmandt R Abstract 4887: Omentin: A Novel Adipokine in the Omental Microenvironment Associated with Ovarian Cancer Progression. Cancer Res (2014) 74(19 Suppl. ment):4887.

[156] Kenny HA Chiang C-Y White EA Schryver EM Habis M Romero IL Mesothelial Cells Promote Early Ovarian Cancer Metastasis through Fibronectin Secretion. J Clin Invest (2014) 124(10):4614–28. 10.1172/jci74778 25202979 PMC4191043

[157] Hiyoshi M Tsuno NH Otani K Kawai K Nishikawa T Shuno Y Adiponectin Receptor 2 Is Negatively Associated with Lymph Node Metastasis of Colorectal Cancer. Oncol Lett (2012) 3(4):756–60. 10.3892/ol.2012.583 22740988 PMC3362449

[158] Taliaferro-Smith L Nagalingam A Zhong D Zhou W Saxena NK Sharma D. LKB1 Is Required for Adiponectin-Mediated Modulation of AMPK-S6k axis and Inhibition of Migration and Invasion of Breast Cancer Cells. Oncogene (2009) 28(29):2621–33. 10.1038/onc.2009.129 19483724 PMC2945727

[159] Corradetti MN Inoki K Bardeesy N DePinho RA Guan K-L. Regulation of the TSC Pathway by LKB1: Evidence of a Molecular Link between Tuberous Sclerosis Complex and Peutz-Jeghers Syndrome. Genes Develop (2004) 18(13):1533–8. 10.1101/gad.1199104 15231735 PMC443516

[160] Gwinn DM Shackelford DB Egan DF Mihaylova MM Mery A Vasquez DS AMPK Phosphorylation of Raptor Mediates a Metabolic Checkpoint. Mol Cel (2008) 30(2):214–26. 10.1016/j.molcel.2008.03.003 PMC2674027 18439900

[161] Amaral CL Freitas LB Tamura RE Tavares MR Pavan ICB Bajgelman MC S6Ks Isoforms Contribute to Viability, Migration, Docetaxel Resistance and Tumor Formation of Prostate Cancer Cells. BMC cancer (2016) 16:602. 10.1186/s12885-016-2629-y 27491285 PMC4974797

[162] Zhang S Hu B Lv X Chen S Liu W Shao Z. The Prognostic Role of Ribosomal Protein S6 Kinase 1 Pathway in Patients with Solid Tumors: A Meta-Analysis. Front Oncol (2019) 9(390). 10.3389/fonc.2019.00390 PMC6527894 31139572

[163] Mauro L Pellegrino M Giordano F Ricchio E Rizza P De Amicis F Estrogen Receptor‐α Drives Adiponectin Effects on Cyclin D1 Expression in Breast Cancer Cells. FASEB j. (2015) 29(5):2150–60. 10.1096/fj.14-262808 25657113

[164] Wang Y Lam JB Lam KSL Liu J Lam MC Hoo RLC Adiponectin Modulates the Glycogen Synthase Kinase-3β/β-Catenin Signaling Pathway and Attenuates Mammary Tumorigenesis of MDA-MB-231 Cells in Nude Mice. Cancer Res (2006) 66(23):11462–70. 10.1158/0008-5472.can-06-1969 17145894

[165] Miyazaki T Bub JD Uzuki M Iwamoto Y. Adiponectin Activates C-Jun NH2-terminal Kinase and Inhibits Signal Transducer and Activator of Transcription 3. Biochem Biophysical Res Commun (2005) 333(1):79–87. 10.1016/j.bbrc.2005.05.076 15936715

[166] Partida-Pérez M de la Luz Ayala-Madrigal M Peregrina-Sandoval J Macías-Gómez N Moreno-Ortiz J Leal-Ugarte E Association of LEP and ADIPOQ Common Variants with Colorectal Cancer in Mexican Patients. Cancer Biomark (2010) 7(3):117–21. 10.3233/CBM-2010-0154 21263187 PMC12922883

[167] Li G Cong L Gasser J Zhao J Chen K Li F. Mechanisms Underlying the Anti-proliferative Actions of Adiponectin in Human Breast Cancer Cells, MCF7-Dependency on the cAMP/protein Kinase-A Pathway. Nutr Cancer (2011) 63(1):80–8. 10.1080/01635581.2010.516472 21108124

[168] Körner A Pazaitou-Panayiotou K Kelesidis T Kelesidis I Williams CJ Kaprara A Total and High-Molecular-Weight Adiponectin in Breast Cancer: *In Vitro* and *In Vivo* Studies. J Clin Endocrinol Metab (2007) 92(3):1041–8. 10.1210/jc.2006-1858 17192291

[169] Cong L Gasser J Zhao J Yang B Li F Zhao AZ. Human Adiponectin Inhibits Cell Growth and Induces Apoptosis in Human Endometrial Carcinoma Cells, HEC-1-A and RL95-2. Endocr Relat Cancer (2007) 14(3):713–20. 10.1677/erc-07-0065 17914101

[170] Dieudonne M-N Bussiere M Dos Santos E Leneveu M-C Giudicelli Y Pecquery R. Adiponectin Mediates Antiproliferative and Apoptotic Responses in Human MCF7 Breast Cancer Cells. Biochem Biophysical Res Commun (2006) 345(1):271–9. 10.1016/j.bbrc.2006.04.076 16678125

[171] Zakikhani M Dowling RJO Sonenberg N Pollak MN. The Effects of Adiponectin and Metformin on Prostate and colon Neoplasia Involve Activation of AMP-Activated Protein Kinase. Cancer Prev Res (2008) 1(5):369–75. 10.1158/1940-6207.capr-08-0081 19138981

[172] Fenton JI Birmingham JM Hursting SD Hord NG. Adiponectin Blocks Multiple Signaling Cascades Associated with Leptin-Induced Cell Proliferation inApcMin/+ colon Epithelial Cells. Int J Cancer (2008) 122(11):2437–45. 10.1002/ijc.23436 18338750

[173] Jardé T Perrier S Vasson M-P Caldefie-Chézet F. Molecular Mechanisms of Leptin and Adiponectin in Breast Cancer. Eur J Cancer (2011) 47(1):33–43. 10.1016/j.ejca.2010.09.005 20889333

[174] Luo Z Saha AK Xiang X Ruderman NB. AMPK, the Metabolic Syndrome and Cancer. Trends Pharmacol Sci (2005) 26(2):69–76. 10.1016/j.tips.2004.12.011 15681023

[175] Kim AY Lee YS Kim KH Lee JH Lee HK Jang S-H Adiponectin Represses Colon Cancer Cell Proliferation via AdipoR1- and -R2-Mediated AMPK Activation. Mol Endocrinol (2010) 24(7):1441–52. 10.1210/me.2009-0498 20444885 PMC5417469

[176] Kitajima D Kasamatsu A Nakashima D Miyamoto I Kimura Y Saito T Tie2 Regulates Tumor Metastasis of Oral Squamous Cell Carcinomas. J Cancer (2016) 7(5):600–7. 10.7150/jca.13820 27053959 PMC4820737

[177] Kitajima D Kasamatsu A Nakashima D Miyamoto I Kimura Y Endo-Sakamoto Y Evidence for Critical Role of Tie2/Ang1 Interaction in Metastatic Oral Cancer. Oncol Lett (2018) 15(5):7237–42. 10.3892/ol.2018.8212 29731883 PMC5920920

[178] Michael IP Orebrand M Lima M Pereira B Volpert O Quaggin SE Angiopoietin-1 Deficiency Increases Tumor Metastasis in Mice. BMC cancer (2017) 17(1):539. 10.1186/s12885-017-3531-y 28800750 PMC5553747

[179] Wu FT Lee CR Bogdanovic E Prodeus A Gariépy J Kerbel RS. Vasculotide Reduces Endothelial Permeability and Tumor Cell Extravasation in the Absence of Binding to or Agonistic Activation of Tie2. EMBO Mol Med (2015) 7(6):770–87. 10.15252/emmm.201404193 25851538 PMC4459817

[180] Ou X-L Chen H-J Sun W-H Hang C Yang L Guan Y-Y Effects of Angiopoietin-1 on Attachment and Metastasis Ofhuman Gastric Cancer Cell Line BGC-823. Wjg (2009) 15(43):5432–41. 10.3748/wjg.15.5432 19916173 PMC2778099

[181] Holopainen T Huang H Chen C Kim KE Zhang L Zhou F Angiopoietin-1 Overexpression Modulates Vascular Endothelium to Facilitate Tumor Cell Dissemination and Metastasis Establishment. Cancer Res (2009) 69(11):4656–64. 10.1158/0008-5472.can-08-4654 19487284

[182] Xu Y Liu Y-j. Yu Q. Angiopoietin-3 Inhibits Pulmonary Metastasis by Inhibiting Tumor Angiogenesis. Cancer Res (2004) 64(17):6119–26. 10.1158/0008-5472.can-04-1054 15342395

[183] Imanishi Y Hu B Jarzynka MJ Guo P Elishaev E Bar-Joseph I Angiopoietin-2 Stimulates Breast Cancer Metastasis through the α5β1 Integrin-Mediated Pathway. Cancer Res (2007) 67(9):4254–63. 10.1158/0008-5472.can-06-4100 17483337 PMC2881574

[184] Li C Li Q Cai Y He Y Lan X Wang W Overexpression of Angiopoietin 2 Promotes the Formation of Oral Squamous Cell Carcinoma by Increasing Epithelial-Mesenchymal Transition-Induced Angiogenesis. Cancer Gene Ther (2016) 23(9):295–302. 10.1038/cgt.2016.30 27492854 PMC5033983

[185] Han HH Kim BG Lee JH Kang S Kim JE Cho NH. Angiopoietin-2 Promotes ER+ Breast Cancer Cell Survival in Bone Marrow Niche. Endocr Relat Cancer (2016) 23(8):609–23. 10.1530/erc-16-0086 27353038 PMC5064757

[186] Sasaki H Suzuki A Shitara M Hikosaka Y Okuda K Moriyama S Angiopoietin-like Protein ANGPTL2 Gene Expression Is Correlated with Lymph Node Metastasis in Lung Cancer. Oncol Lett (2012) 4(6):1325–8. 10.3892/ol.2012.924 23205131 PMC3506759

[187] Aoi J Endo M Kadomatsu T Miyata K Nakano M Horiguchi H Angiopoietin-like Protein 2 Is an Important Facilitator of Inflammatory Carcinogenesis and Metastasis. Cancer Res (2011) 71(24):7502–12. 10.1158/0008-5472.can-11-1758 22042794

[188] Endo M Nakano M Kadomatsu T Fukuhara S Kuroda H Mikami S Tumor Cell-Derived Angiopoietin-like Protein ANGPTL2 Is a Critical Driver of Metastasis. Cancer Res (2012) 72(7):1784–94. 10.1158/0008-5472.can-11-3878 22345152

[189] Wang X Hu Z Wang Z Cui Y Cui X. Angiopoietin-like Protein 2 Is an Important Facilitator of Tumor Proliferation, Metastasis, Angiogenesis and Glycolysis in Osteosarcoma. Am J Transl Res (2019) 11(10):6341–55. 31737187 PMC6834488

[190] Odagiri H Kadomatsu T Endo M Masuda T Morioka MS Fukuhara S The Secreted Protein ANGPTL2 Promotes Metastasis of Osteosarcoma Cells through Integrin 5 1, P38 MAPK, and Matrix Metalloproteinases. Sci Signaling (2014) 7(309):ra7. 10.1126/scisignal.2004612 24448647

[191] Galaup A Cazes A Le Jan S Philippe J Connault E Le Coz E Angiopoietin-like 4 Prevents Metastasis through Inhibition of Vascular Permeability and Tumor Cell Motility and Invasiveness. Proc Natl Acad Sci (2006) 103(49):18721–6. 10.1073/pnas.0609025103 17130448 PMC1693729

[192] Zhang H Wong CCL Wei H Gilkes DM Korangath P Chaturvedi P HIF-1-dependent Expression of Angiopoietin-like 4 and L1CAM Mediates Vascular Metastasis of Hypoxic Breast Cancer Cells to the Lungs. Oncogene (2012) 31(14):1757–70. 10.1038/onc.2011.365 21860410 PMC3223555

[193] Padua D Zhang XH-F Wang Q Nadal C Gerald WL Gomis RR TGFβ Primes Breast Tumors for Lung Metastasis Seeding through Angiopoietin-like 4. Cell (2008) 133(1):66–77. 10.1016/j.cell.2008.01.046 18394990 PMC2390892

[194] Marchiò S Soster M Cardaci S Muratore A Bartolini A Barone V A Complex of α 6 Integrin and E‐cadherin Drives Liver Metastasis of Colorectal Cancer Cells through Hepatic Angiopoietin‐like 6. EMBO Mol Med (2012) 4(11):1156–75. 10.1002/emmm.201101164 23070965 PMC3494873

[195] Chu SH Lee MK Ahn KY Im JA Park MS Lee DC Chemerin and Adiponectin Contribute Reciprocally to Metabolic Syndrome. PLoS One (2012) 7(4):e34710. 10.1371/journal.pone.0034710 22509348 PMC3324504

[196] Li J-J Yin H-K Guan D-X Zhao J-S Feng Y-X Deng Y-Z Chemerin Suppresses Hepatocellular Carcinoma Metastasis through CMKLR1-PTEN-Akt axis. Br J Cancer (2018) 118(10):1337–48. 10.1038/s41416-018-0077-y 29717200 PMC5959946

[197] Liu-Chittenden Y Jain M Gaskins K Wang S Merino MJ Kotian S RARRES2 Functions as a Tumor Suppressor by Promoting β-catenin Phosphorylation/degradation and Inhibiting P38 Phosphorylation in Adrenocortical Carcinoma. Oncogene (2017) 36(25):3541–52. 10.1038/onc.2016.497 28114280 PMC5481486

[198] Zhang J Zhou J Tang X Zhou L-Y Zhai L-L Vanessa ME-D Reduced Expression of Chemerin Is Associated with Poor Clinical Outcome in Acute Myeloid Leukemia. Oncotarget (2017) 8(54):92536–44. 10.18632/oncotarget.21440 29190935 PMC5696201

[199] Xu C-H Yang Y Wang Y-C Yan J Qian L-H. Prognostic Significance of Serum Chemerin Levels in Patients with Non-small Cell Lung Cancer. Oncotarget (2017) 8(14):22483–9. 10.18632/oncotarget.14956 28160556 PMC5410238

[200] Zhang J Jin H-C Zhu A-K Ying R-C Wei W Zhang F-J. Prognostic Significance of Plasma Chemerin Levels in Patients with Gastric Cancer. Peptides (2014) 61:7–11. 10.1016/j.peptides.2014.08.007 25152503

[201] El-Sagheer G Gayyed M Ahmad A Abd El-Fattah A Mohamed M. Expression of Chemerin Correlates with a Poor Prognosis in Female Breast Cancer Patients. Breast Cancer (Dove Med Press) (2018) 10:169–76. 10.2147/bctt.s178181 30498371 PMC6207381

[202] De Henau O Degroot G-N Imbault V Robert V De Poorter C McHeik S Signaling Properties of Chemerin Receptors CMKLR1, GPR1 and CCRL2. PloS one (2016) 11(10):e0164179. 10.1371/journal.pone.0164179 27716822 PMC5055294

[203] Duan X Kong Z Liu Y Zeng Z Li S Wu W β-Arrestin2 Contributes to Cell Viability and Proliferation via the Down-Regulation of FOXO1 in Castration-Resistant Prostate Cancer. J Cel Physiol. (2015) 230(10):2371–81. 10.1002/jcp.24963 25752515

[204] Ge L Shenoy SK Lefkowitz RJ DeFea K. Constitutive Protease-Activated Receptor-2-Mediated Migration of MDA MB-231 Breast Cancer Cells Requires Both β-Arrestin-1 and -2. J Biol Chem (2004) 279(53):55419–24. 10.1074/jbc.m410312200 15489220

[205] Lakshmikanthan V Zou L Kim JI Michal A Nie Z Messias NC Identification of Arrestin2 as a Corepressor of Androgen Receptor Signaling in Prostate Cancer. Proc Natl Acad Sci (2009) 106(23):9379–84. 10.1073/pnas.0900258106 19458261 PMC2695075

[206] Cong L Qiu Z-y. Zhao Y Wang W-b. Wang C-x. Shen H-c. Loss of β-arrestin-2 and Activation of CXCR2 Correlate with Lymph Node Metastasis in Non-small Cell Lung Cancer. J Cancer (2017) 8(14):2785–92. 10.7150/jca.19631 28928867 PMC5604210

[207] Sun W-Y Hu S-S Wu J-J Huang Q Ma Y Wang Q-T Down-regulation of β-arrestin2 Promotes Tumour Invasion and Indicates Poor Prognosis of Hepatocellular Carcinoma. Scientific Rep (2016) 6(1):35609. 10.1038/srep35609 PMC5069669 27759077

[208] DeFea KA Zalevsky J Thoma MS Déry O Mullins RD Bunnett NW. β-Arrestin-Dependent Endocytosis of Proteinase-Activated Receptor 2 Is Required for Intracellular Targeting of Activated Erk1/2. J Cel Biol (2000) 148(6):1267–82. 10.1083/jcb.148.6.1267 PMC2174299 10725339

[209] Rourke JL Dranse HJ Sinal CJ. CMKLR1 and GPR1 Mediate Chemerin Signaling through the RhoA/ROCK Pathway. Mol Cell Endocrinol (2015) 417:36–51. 10.1016/j.mce.2015.09.002 26363224

[210] Yin J Lv X Hu S Zhao X Liu Q Xie H. Overexpression of Serum Response Factor Is Correlated with Poor Prognosis in Patients with Gastric Cancer. Hum Pathol (2019) 85:10–7. 10.1016/j.humpath.2018.10.018 30500416

[211] O'Hurley G Prencipe M Lundon D O'Neill A Boyce S O'Grady A The Analysis of Serum Response Factor Expression in Bone and Soft Tissue Prostate Cancer Metastases. Prostate (2014) 74(3):306–13. 10.1002/pros.22752 24249383

[212] Cen B Selvaraj A Burgess RC Hitzler JK Ma Z Morris SW Megakaryoblastic Leukemia 1, a Potent Transcriptional Coactivator for Serum Response Factor (SRF), Is Required for Serum Induction of SRF Target Genes. Mol Cel Biol (2003) 23(18):6597–608. 10.1128/mcb.23.18.6597-6608.2003 PMC193697 12944485

[213] Selvaraj A Prywes R. Expression Profiling of Serum Inducible Genes Identifies a Subset of SRF Target Genes that Are MKL Dependent. BMC Mol Biol (2004) 5(1):13. 10.1186/1471-2199-5-13 15329155 PMC516031

[214] Nair P Muthukkumar S Sells SF Han S-S Sukhatme VP Rangnekar VM. Early Growth Response-1-dependent Apoptosis Is Mediated by P53. J Biol Chem (1997) 272(32):20131–8. 10.1074/jbc.272.32.20131 9242687

[215] Krones-Herzig A Adamson E Mercola D. Early Growth Response 1 Protein, an Upstream Gatekeeper of the P53 Tumor Suppressor, Controls Replicative Senescence. Proc Natl Acad Sci (2003) 100(6):3233–8. 10.1073/pnas.2628034100 12629205 PMC152275

[216] Li L Huang C Zhang X Wang J Ma P Liu Y Chemerin-derived Peptide C-20 Suppressed Gonadal Steroidogenesis. Am J Reprod Immunol (2014) 71(3):265–77. 10.1111/aji.12164 24506805 PMC4092037

[217] Muruganandan S Dranse HJ Rourke JL McMullen NM Sinal CJ. Chemerin Neutralization Blocks Hematopoietic Stem Cell Osteoclastogenesis. Stem Cells (2013) 31(10):2172–82. 10.1002/stem.1450 23766088

[218] Guo JC Li J Zhao YP Zhou L Cui QC Zhou WX Expression of C-Fos Was Associated with Clinicopathologic Characteristics and Prognosis in Pancreatic Cancer. PLoS One (2015) 10(3):e0120332. 10.1371/journal.pone.0120332 25789763 PMC4366380

[219] Lu C Shen Q DuPré E Kim H Hilsenbeck S Brown PH. cFos Is Critical for MCF-7 Breast Cancer Cell Growth. Oncogene (2005) 24(43):6516–24. 10.1038/sj.onc.1208905 16027729

[220] Mikula M Gotzmann J Fischer ANM Wolschek MF Thallinger C Schulte-Hermann R The Proto-Oncoprotein C-Fos Negatively Regulates Hepatocellular Tumorigenesis. Oncogene (2003) 22(43):6725–38. 10.1038/sj.onc.1206781 14555986

[221] Mahner S Baasch C Schwarz J Hein S Wölber L Jänicke F C-fos Expression Is a Molecular Predictor of Progression and Survival in Epithelial Ovarian Carcinoma. Br J Cancer (2008) 99(8):1269–75. 10.1038/sj.bjc.6604650 18854825 PMC2570515

[222] Oliveira-Ferrer L Rößler K Haustein V Schröder C Wicklein D Maltseva D c-FOS Suppresses Ovarian Cancer Progression by Changing Adhesion. Br J Cancer (2014) 110(3):753–63. 10.1038/bjc.2013.774 24322891 PMC3915133

[223] Zhang X Zhang L Yang H Huang X Otu H Libermann TA c-Fos as a Proapoptotic Agent in TRAIL-Induced Apoptosis in Prostate Cancer Cells. Cancer Res (2007) 67(19):9425–34. 10.1158/0008-5472.can-07-1310 17909052 PMC2941899

[224] Li T Guo H Song Y Zhao X Shi Y Lu Y Loss of Vinculin and Membrane-Bound β-catenin Promotes Metastasis and Predicts Poor Prognosis in Colorectal Cancer. Mol Cancer (2014) 13(1):263. 10.1186/1476-4598-13-263 25496021 PMC4320448

[225] Zhang M Liu P Xu F He Y Xie X Jiang X. Retracted : Vinculin Promotes Gastric Cancer Proliferation and Migration and Predicts Poor Prognosis in Patients with Gastric Cancer. J Cel Biochem (2019) 120(8):14107–15. 10.1002/jcb.28686 30989694

[226] Pan Y Zhou F He C Hui L Huang T Wei Y. Leptin-LepRb Expressed in Gastric Cancer Patients and Related to Cancer-Related Depression. Biomed Res Int (2017) 2017:6482842. 10.1155/2017/6482842 28316984 PMC5337857

[227] Cheng H Wang Z Fu L Xu T. Macrophage Polarization in the Development and Progression of Ovarian Cancers: An Overview. Front Oncol (2019) 9:421. 10.3389/fonc.2019.00421 31192126 PMC6540821

[228] Yafei Z Jun G Guolan G. Correlation between Macrophage Infiltration and Prognosis of Ovarian Cancer-A Preliminary Study. Biomed Res (2016) 27:305–12.

[229] Tian Z Hou X Liu W Han Z Wei L. Macrophages and Hepatocellular Carcinoma. Cel Biosci (2019) 9(1):79. 10.1186/s13578-019-0342-7 PMC6761725 31572568

[230] Jeong H Hwang I Kang SH Shin HC Kwon SY. Tumor-Associated Macrophages as Potential Prognostic Biomarkers of Invasive Breast Cancer. J Breast Cancer (2019) 22(1):38–51. 10.4048/jbc.2019.22.e5 30941232 PMC6438840

[231] Lee YS Song SJ Hong HK Oh BY Lee WY Cho YB. The FBW7-MCL-1 axis Is Key in M1 and M2 Macrophage-Related colon Cancer Cell Progression: Validating the Immunotherapeutic Value of Targeting PI3Kγ. Exp Mol Med (2020) 52(5):815–31. 10.1038/s12276-020-0436-7 32444799 PMC7272616

[232] Herová M Schmid M Gemperle C Hersberger M. ChemR23, the Receptor for Chemerin and Resolvin E1, Is Expressed and Functional on M1 but Not on M2 Macrophages. J.I. (2015) 194(5):2330–7. 10.4049/jimmunol.1402166 25637017

[233] Pachynski RK Zabel BA Kohrt HE Tejeda NM Monnier J Swanson CD The Chemoattractant Chemerin Suppresses Melanoma by Recruiting Natural Killer Cell Antitumor Defenses. J Exp Med (2012) 209(8):1427–35. 10.1084/jem.20112124 22753924 PMC3409495

[234] Dimitriadis GK Kaur J Adya R Miras AD Mattu HS Hattersley JG Chemerin Induces Endothelial Cell Inflammation: Activation of Nuclear Factor-Kappa Beta and Monocyte-Endothelial Adhesion. Oncotarget (2018) 9(24):16678–90. 10.18632/oncotarget.24659 29682177 PMC5908278

[235] Zylla S Pietzner M Kühn J-P Völzke H Dörr M Nauck M Serum Chemerin Is Associated with Inflammatory and Metabolic Parameters-Results of a Population-Based Study. Obesity (2017) 25(2):468–75. 10.1002/oby.21735 28071854

[236] Liu S Hempe JM McCarter RJ Li S Fonseca VA. Association between Inflammation and Biological Variation in Hemoglobin A1c in U.S. Nondiabetic Adults. J Clin Endocrinol Metab (2015) 100(6):2364–71. 10.1210/jc.2014-4454 25867810 PMC4454807

[237] Kaur J Adya R Tan BK Chen J Randeva HS. Identification of Chemerin Receptor (ChemR23) in Human Endothelial Cells: Chemerin-Induced Endothelial Angiogenesis. Biochem Biophysical Res Commun (2010) 391(4):1762–8. 10.1016/j.bbrc.2009.12.150 20044979

[238] Bozaoglu K Curran JE Stocker CJ Zaibi MS Segal D Konstantopoulos N Chemerin, a Novel Adipokine in the Regulation of Angiogenesis. J Clin Endocrinol Metab (2010) 95(5):2476–85. 10.1210/jc.2010-0042 20237162 PMC2869547

[239] Wang C Wu WKK Liu X To K-F Chen GG Yu J Increased Serum Chemerin Level Promotes Cellular Invasiveness in Gastric Cancer: a Clinical and Experimental Study. Peptides (2014) 51:131–8. 10.1016/j.peptides.2013.10.009 24274970

[240] Nakamura N Naruse K Kobayashi Y Miyabe M Saiki T Enomoto A Chemerin Promotes Angiogenesis *In Vivo* . Physiol Rep (2018) 6(24):e13962. 10.14814/phy2.13962 30588761 PMC6306368

[241] Skrzeczynska-Moncznik J Wawro K Stefanska A Oleszycka E Kulig P Zabel BA Potential Role of Chemerin in Recruitment of Plasmacytoid Dendritic Cells to Diseased Skin. Biochem biophysical Res Commun (2009) 380:323–7. 10.1016/j.bbrc.2009.01.071 PMC6289204 19168032

[242] Bidwell BN Slaney CY Withana NP Forster S Cao Y Loi S Silencing of Irf7 Pathways in Breast Cancer Cells Promotes Bone Metastasis through Immune Escape. Nat Med (2012) 18(8):1224–31. 10.1038/nm.2830 22820642

[243] Bekeredjian-Ding I Schäfer M Hartmann E Pries R Parcina M Schneider P Tumour-derived Prostaglandin E2and Transforming Growth Factor-β Synergize to Inhibit Plasmacytoid Dendritic Cell-Derived Interferon-α. Immunology (2009) 128(3):439–50. 10.1111/j.1365-2567.2009.03134.x 20067543 PMC2770691

[244] Hartmann E Wollenberg B Rothenfusser S Wagner M Wellisch D Mack B Identification and Functional Analysis of Tumor-Infiltrating Plasmacytoid Dendritic Cells in Head and Neck Cancer. Cancer Res (2003) 63(19):6478–87. 14559840

[245] Auguet T Quintero Y Terra X Martínez S Lucas A Pellitero S Upregulation of Lipocalin 2 in Adipose Tissues of Severely Obese Women: Positive Relationship with Proinflammatory Cytokines. Obesity (Silver Spring) (2011) 19(12):2295–300. 10.1038/oby.2011.61 21455126

[246] Lee H-J Lee E-K Lee K-J Hong S-W Yoon Y Kim J-S. Ectopic Expression of Neutrophil Gelatinase-Associated Lipocalin Suppresses the Invasion and Liver Metastasis of colon Cancer Cells. Int J Cancer (2006) 118(10):2490–7. 10.1002/ijc.21657 16381001

[247] Feng M Feng J Chen W Wang W Wu X Zhang J Lipocalin2 Suppresses Metastasis of Colorectal Cancer by Attenuating NF-κb-dependent Activation of Snail and Epithelial Mesenchymal Transition. Mol Cancer (2016) 15(1):77. 10.1186/s12943-016-0564-9 27912767 PMC5135816

[248] Moniaux N Chakraborty S Yalniz M Gonzalez J Shostrom VK Standop J Early Diagnosis of Pancreatic Cancer: Neutrophil Gelatinase-Associated Lipocalin as a Marker of Pancreatic Intraepithelial Neoplasia. Br J Cancer (2008) 98(9):1540–7. 10.1038/sj.bjc.6604329 18392050 PMC2391106

[249] Yang J Bielenberg DR Rodig SJ Doiron R Clifton MC Kung AL Lipocalin 2 Promotes Breast Cancer Progression. Proc Natl Acad Sci (2009) 106(10):3913–8. 10.1073/pnas.0810617106 19237579 PMC2656179

[250] Zhang Y Lazarus J Steele NG Yan W Lee H-J Nwosu ZC Regulatory T-Cell Depletion Alters the Tumor Microenvironment and Accelerates Pancreatic Carcinogenesis. Cancer Discov (2020) 10(3):422–39. 10.1158/2159-8290.cd-19-0958 31911451 PMC7224338

[251] Karlsen JR Borregaard N Cowland JB. Induction of Neutrophil Gelatinase-Associated Lipocalin Expression by Co-stimulation with Interleukin-17 and Tumor Necrosis Factor-α Is Controlled by IκB-ζ but Neither by C/EBP-β Nor C/EBP-δ. J Biol Chem (2010) 285(19):14088–100. 10.1074/jbc.m109.017129 20220144 PMC2863242

[252] Wani NA Nasser MW Ahirwar DK Zhao H Miao Z Shilo K C-X-C Motif Chemokine 12/C-X-C Chemokine Receptor Type 7 Signaling Regulates Breast Cancer Growth and Metastasis by Modulating the Tumor Microenvironment. Breast Cancer Res (2014) 16(3):R54. 10.1186/bcr3665 24886617 PMC4076630

[253] Gomez-Chou SB Swidnicka-Siergiejko AK Badi N Chavez-Tomar M Lesinski GB Bekaii-Saab T Lipocalin-2 Promotes Pancreatic Ductal Adenocarcinoma by Regulating Inflammation in the Tumor Microenvironment. Cancer Res (2017) 77(10):2647–60. 10.1158/0008-5472.can-16-1986 28249896 PMC5441230

[254] Tschesche H Zölzer V Triebel S Bartsch S. The Human Neutrophil Lipocalin Supports the Allosteric Activation of Matrix Metalloproteinases. Eur J Biochem (2001) 268(7):1918–28. 10.1046/j.1432-1327.2001.02066.x 11277914

[255] Kubben FJGM Sier CFM Hawinkels LJAC Tschesche H van Duijn W Zuidwijk K Clinical Evidence for a Protective Role of Lipocalin-2 against MMP-9 Autodegradation and the Impact for Gastric Cancer. Eur J Cancer (2007) 43(12):1869–76. 10.1016/j.ejca.2007.05.013 17604154

[256] Nuntagowat C Leelawat K Tohtong R. NGAL Knockdown by siRNA in Human Cholangiocarcinoma Cells Suppressed Invasion by Reducing NGAL/MMP-9 Complex Formation. Clin Exp Metastasis (2010) 27(5):295–305. 10.1007/s10585-010-9327-y 20373132

[257] Fernández CA Yan L Louis G Yang J Kutok JL Moses MA. The Matrix Metalloproteinase-9/neutrophil Gelatinase-Associated Lipocalin Complex Plays a Role in Breast Tumor Growth and Is Present in the Urine of Breast Cancer Patients. Clin Cancer Res (2005) 11(15):5390–5. 10.1158/1078-0432.ccr-04-2391 16061852

[258] Volpe V Raia Z Sanguigno L Somma D Mastrovito P Moscato F NGAL Controls the Metastatic Potential of Anaplastic Thyroid Carcinoma Cells. J Clin Endocrinol Metab (2013) 98(1):228–35. 10.1210/jc.2012-2528 23150684

[259] Roy R Louis G Loughlin KR Wiederschain D Kilroy SM Lamb CC Tumor-specific Urinary Matrix Metalloproteinase Fingerprinting: Identification of High Molecular Weight Urinary Matrix Metalloproteinase Species. Clin Cancer Res (2008) 14(20):6610–7. 10.1158/1078-0432.ccr-08-1136 18927302 PMC2879331

[260] Leng X Ding T Lin H Wang Y Hu L Hu J Inhibition of Lipocalin 2 Impairs Breast Tumorigenesis and Metastasis. Cancer Res (2009) 69(22):8579–84. 10.1158/0008-5472.can-09-1934 19887608

[261] Iannetti A Pacifico F Acquaviva R Lavorgna A Crescenzi E Vascotto C The Neutrophil Gelatinase-Associated Lipocalin (NGAL), a NF- B-Regulated Gene, Is a Survival Factor for Thyroid Neoplastic Cells. Proc Natl Acad Sci (2008) 105(37):14058–63. 10.1073/pnas.0710846105 18768801 PMC2544578

[262] Leung L Radulovich N Zhu CQ Organ S Bandarchi B Pintilie M Lipocalin2 Promotes Invasion, Tumorigenicity and Gemcitabine Resistance in Pancreatic Ductal Adenocarcinoma. PLoS One (2012) 7(10):e46677. 10.1371/journal.pone.0046677 23056397 PMC3464270

[263] Hanai J-i. Mammoto T Seth P Mori K Karumanchi SA Barasch J Lipocalin 2 Diminishes Invasiveness and Metastasis of Ras-Transformed Cells. J Biol Chem (2005) 280(14):13641–7. 10.1074/jbc.m413047200 15691834

[264] Shi H Gu Y Yang J Xu L Mi W Yu W. Lipocalin 2 Promotes Lung Metastasis of Murine Breast Cancer Cells. J Exp Clin Cancer Res (2008) 27(1):83. 10.1186/1756-9966-27-83 19077278 PMC2614970

[265] Gu Y Zhang J Mi W Yang J Han F Lu X Silencing of GM3 Synthase Suppresses Lung Metastasis of Murine Breast Cancer Cells. Breast Cancer Res (2008) 10(1):R1. 10.1186/bcr1841 18171481 PMC2374951

[266] Li B Xu WW Lam AKY Wang Y Hu H-F Guan XY Significance of PI3K/AKT Signaling Pathway in Metastasis of Esophageal Squamous Cell Carcinoma and its Potential as a Target for Anti-metastasis Therapy. Oncotarget (2017) 8(24):38755–66. 10.18632/oncotarget.16333 28418888 PMC5503569

[267] Yue Y Hui K Wu S Zhang M Que T Gu Y MUC15 Inhibits Cancer Metastasis via PI3K/AKT Signaling in Renal Cell Carcinoma. Cel Death Dis (2020) 11(5):336. 10.1038/s41419-020-2518-9 PMC7205982 32382053

[268] Liu H Radisky DC Nelson CM Zhang H Fata JE Roth RA Mechanism of Akt1 Inhibition of Breast Cancer Cell Invasion Reveals a Protumorigenic Role for TSC2. Proc Natl Acad Sci (2006) 103(11):4134–9. 10.1073/pnas.0511342103 16537497 PMC1390746

[269] Qin J-J Li X Wang W Zi X Zhang R. Targeting the NFAT1-MDM2-MDMX Network Inhibits the Proliferation and Invasion of Prostate Cancer Cells, Independent of P53 and Androgen. Front Pharmacol (2017) 8:917. 10.3389/fphar.2017.00917 29311926 PMC5735069

[270] Bao G Clifton M Hoette TM Mori K Deng S-X Qiu A Iron Traffics in Circulation Bound to a Siderocalin (Ngal)-Catechol Complex. Nat Chem Biol (2010) 6(8):602–9. 10.1038/nchembio.402 20581821 PMC2907470

[271] Schafer ZT Grassian AR Song L Jiang Z Gerhart-Hines Z Irie HY Antioxidant and Oncogene rescue of Metabolic Defects Caused by Loss of Matrix Attachment. Nature (2009) 461(7260):109–13. 10.1038/nature08268 19693011 PMC2931797

[272] Devireddy LR Gazin C Zhu X Green MR. A Cell-Surface Receptor for Lipocalin 24p3 Selectively Mediates Apoptosis and Iron Uptake. Cell (2005) 123(7):1293–305. 10.1016/j.cell.2005.10.027 16377569

[273] Zhang S Chen Y Guo W Yuan L Zhang D Xu Y Disordered Hepcidin-Ferroportin Signaling Promotes Breast Cancer Growth. Cell Signal (2014) 26(11):2539–50. 10.1016/j.cellsig.2014.07.029 25093806

[274] Deng Y Wang ZV Gordillo R An Y Zhang C Liang Q An Adipo-Biliary-Uridine axis that Regulates Energy Homeostasis. Science (2017) 355(6330). 10.1126/science.aaf5375 PMC5832364 28302796

[275] Kohli R Bhattacharjee J Inge TH. Postprandial Uridine Physiology Is Altered by Obesity. Gastroenterology (2018) 155(5):1645–6. 10.1053/j.gastro.2018.07.043 30142337 PMC6428419

[276] Placet M Arguin G Molle CM Babeu J-P Jones C Carrier JC The G Protein-Coupled P2Y6 Receptor Promotes Colorectal Cancer Tumorigenesis by Inhibiting Apoptosis. Biochim Biophys Acta (Bba) - Mol Basis Dis (2018) 1864(5):1539–51. 10.1016/j.bbadis.2018.02.008 29454075

[277] Qiu Y Liu Y Li WH Zhang HQ Tian XX Fang WG. P2Y2 Receptor Promotes the Migration and Invasion of Breast Cancer Cells via EMT-Related Genes Snail and E-Cadherin. Oncol Rep (2018) 39(1):138–50. 10.3892/or.2017.6081 29115551 PMC5783596

[278] Ma X Pan X Wei Y Tan B Yang L Ren H Chemotherapy-induced Uridine Diphosphate Release Promotes Breast Cancer Metastasis through P2Y6 Activation. Oncotarget (2016) 7(20):29036–50. 10.18632/oncotarget.8664 27074554 PMC5045376

[279] Miyashita H Takebayashi Y Eliason JF Fujimori F Nitta Y Sato A Uridine Phosphorylase Is a Potential Prognostic Factor in Patients with Oral Squamous Cell Carcinoma. Cancer (2002) 94(11):2959–66. 10.1002/cncr.10568 12115385

[280] Shen G He P Mao Y Li P Luh F Ding G Overexpression of Uridine-Cytidine Kinase 2 Correlates with Breast Cancer Progression and Poor Prognosis. J Breast Cancer (2017) 20(2):132–41. 10.4048/jbc.2017.20.2.132 28690649 PMC5500396

[281] Guan Y Bhandari A Zhang X Wang O. Uridine Phosphorylase 1 Associates to Biological and Clinical Significance in Thyroid Carcinoma Cell Lines. J Cel Mol Med (2019) 23(11):7438–48. 10.1111/jcmm.14612 PMC6815846 31496029

[282] Wu Y Jamal M Xie T Sun J Song T Yin Q Uridine‐cytidine Kinase 2 (UCK2): A Potential Diagnostic and Prognostic Biomarker for Lung Cancer. Cancer Sci (2019) 110(9):2734–47. 10.1111/cas.14125 31278886 PMC6726693

[283] Mazidi M Katsiki N Mikhailidis DP Banach M. The Link between Insulin Resistance Parameters and Serum Uric Acid Is Mediated by Adiposity. Atherosclerosis (2018) 270:180–6. 10.1016/j.atherosclerosis.2017.12.033 29459295

[284] Mele C Tagliaferri MA Saraceno G Mai S Vietti R Zavattaro M Serum Uric Acid Potentially Links Metabolic Health to Measures of Fuel Use in Lean and Obese Individuals. Nutr Metab Cardiovasc Dis (2018) 28(10):1029–35. 10.1016/j.numecd.2018.06.010 30139687

[285] Ali N Perveen R Rahman S Mahmood S Rahman S Islam S Prevalence of Hyperuricemia and the Relationship between Serum Uric Acid and Obesity: A Study on Bangladeshi Adults. PloS one (2018) 13(11):e0206850. 10.1371/journal.pone.0206850 30383816 PMC6211757

[286] Levine W Dyer AR Shekelle RB Schoenberger JA Stamler J. Serum Uric Acid and 11.5-year Mortality of Middle-Aged Women: Findings of the Chicago Heart Association Detection Project in Industry. J Clin Epidemiol (1989) 42(3):257–67. 10.1016/0895-4356(89)90061-9 2709083

[287] Kolonel LN Yoshizawa C Nomura AM Stemmermann GN. Relationship of Serum Uric Acid to Cancer Occurrence in a Prospective Male Cohort. Cancer Epidemiol Biomarkers Prev (1994) 3:225–8. 8019371

[288] Yan S Zhang P Xu W Liu Y Wang B Jiang T Serum Uric Acid Increases Risk of Cancer Incidence and Mortality: A Systematic Review and Meta-Analysis. Mediators Inflamm (2015) 2015:764250. 10.1155/2015/764250 26504361 PMC4609511

[289] Hammarsten J Damber J-E Peeker R Mellström D Högstedt B. A Higher Prediagnostic Insulin Level Is a Prospective Risk Factor for Incident Prostate Cancer. Cancer Epidemiol (2010) 34(5):574–9. 10.1016/j.canep.2010.06.014 20702155

[290] Yim K Bindayi A McKay R Mehrazin R Raheem OA Field C Rising Serum Uric Acid Level Is Negatively Associated with Survival in Renal Cell Carcinoma. Cancers (2019) 11(4):536. 10.3390/cancers11040536 PMC6520981 30991671

[291] Cetin AO Omar M Calp S Tunca H Yimaz N Ozseker B Hyperuricemia at the Time of Diagnosis Is a Factor for Poor Prognosis in Patients with Stage II and III Colorectal Cancer (Uric Acid and Colorectal Cancer). Asian Pac J Cancer Prev (2017) 18(2):485–90. 10.22034/APJCP.2017.18.2.485 28345834 PMC5454747

[292] Subandrate S Lea K Safyudin. The Correlation between Uric Acid and Stages of Malignancy Among Gastric Cancer Patient in Palembang, Indonesia. J Phys Conf Ser (2019) 1246:012062. 10.1088/1742-6596/1246/1/012062

[293] Wang S Liu X He Z Chen X Li W. Hyperuricemia Has an Adverse Impact on the Prognosis of Patients with Osteosarcoma. Tumor Biol (2016) 37(1):1205–10. 10.1007/s13277-015-3830-3 26282000

[294] Fabbrini E Serafini M Colic Baric I Hazen SL Klein S. Effect of Plasma Uric Acid on Antioxidant Capacity, Oxidative Stress, and Insulin Sensitivity in Obese Subjects. Diabetes (2014) 63(3):976–81. 10.2337/db13-1396 24353177 PMC3931399

[295] Gersch C Palii SP Kim KM Angerhofer A Johnson RJ Henderson GN. Inactivation of Nitric Oxide by Uric Acid. Nucleosides, Nucleotides and Nucleic Acids (2008) 27(8):967–78. 10.1080/15257770802257952 PMC2701227 18696365

[296] Wan X Xu C Lin Y Lu C Li D Sang J Uric Acid Regulates Hepatic Steatosis and Insulin Resistance through the NLRP3 Inflammasome-dependent Mechanism. J Hepatol (2016) 64(4):925–32. 10.1016/j.jhep.2015.11.022 26639394

[297] Sautin YY Nakagawa T Zharikov S Johnson RJ. Adverse Effects of the Classic Antioxidant Uric Acid in Adipocytes: NADPH Oxidase-Mediated Oxidative/nitrosative Stress. Am J Physiology-Cell Physiol (2007) 293(2):C584–C596. 10.1152/ajpcell.00600.2006 17428837

[298] Yu M-A Sánchez-Lozada LG Johnson RJ Kang D-H. Oxidative Stress with an Activation of the Renin-Angiotensin System in Human Vascular Endothelial Cells as a Novel Mechanism of Uric Acid-Induced Endothelial Dysfunction. J Hypertens (2010) 28(6):1234–42. 10.1097/hjh.0b013e328337da1d 20486275

[299] Corry DB Eslami P Yamamoto K Nyby MD Makino H Tuck ML. Uric Acid Stimulates Vascular Smooth Muscle Cell Proliferation and Oxidative Stress via the Vascular Renin-Angiotensin System. J Hypertens (2008) 26(2):269–75. 10.1097/hjh.0b013e3282f240bf 18192841

[300] Cirillo P Gersch MS Mu W Scherer PM Kim KM Gesualdo L Ketohexokinase-dependent Metabolism of Fructose Induces Proinflammatory Mediators in Proximal Tubular Cells. Jasn (2009) 20(3):545–53. 10.1681/asn.2008060576 19158351 PMC2653686

[301] Lanaspa MA Sanchez-Lozada LG Choi Y-J Cicerchi C Kanbay M Roncal-Jimenez CA Uric Acid Induces Hepatic Steatosis by Generation of Mitochondrial Oxidative Stress. J Biol Chem (2012) 287(48):40732–44. 10.1074/jbc.m112.399899 23035112 PMC3504786

[302] DeNicola GM Karreth FA Humpton TJ Gopinathan A Wei C Frese K Oncogene-induced Nrf2 Transcription Promotes ROS Detoxification and Tumorigenesis. Nature (2011) 475(7354):106–9. 10.1038/nature10189 21734707 PMC3404470

[303] Ames BN Cathcart R Schwiers E Hochstein P. Uric Acid Provides an Antioxidant Defense in Humans against Oxidant- and Radical-Caused Aging and Cancer: a Hypothesis. Proc Natl Acad Sci (1981) 78(11):6858–62. 10.1073/pnas.78.11.6858 6947260 PMC349151

[304] Linder N Haglund C Lundin M Nordling S Ristimäki A Kokkola A Decreased Xanthine Oxidoreductase Is a Predictor of Poor Prognosis in Early-Stage Gastric Cancer. J Clin Pathol (2006) 59(9):965–71. 10.1136/jcp.2005.032524 16935971 PMC1860491

[305] Linder N Martelin E Lundin M Louhimo J Nordling S Haglund C Xanthine Oxidoreductase - Clinical Significance in Colorectal Cancer and *In Vitro* Expression of the Protein in Human colon Cancer Cells. Eur J Cancer (2009) 45(4):648–55. 10.1016/j.ejca.2008.10.036 19112016

[306] Linder N Lundin J Isola J Lundin M Raivio KO Joensuu H. Down-Regulated Xanthine Oxidoreductase Is a Feature of Aggressive Breast Cancer. Clin Cancer Res (2005) 11:4372–81. 10.1158/1078-0432.ccr-04-2280 15958620

[307] Cheung KJ Tzameli I Pissios P Rovira I Gavrilova O Ohtsubo T Xanthine Oxidoreductase Is a Regulator of Adipogenesis and PPARγ Activity. Cel Metab (2007) 5(2):115–28. 10.1016/j.cmet.2007.01.005 17276354

[308] Fini MA Monks J Farabaugh SM Wright RM. Contribution of Xanthine Oxidoreductase to Mammary Epithelial and Breast Cancer Cell Differentiation in Part Modulates Inhibitor of Differentiation-1. Mol Cancer Res (2011) 9(9):1242–54. 10.1158/1541-7786.mcr-11-0176 21775420 PMC3175308

[309] Fini MA Orchard-Webb D Kosmider B Amon JD Kelland R Shibao G Migratory Activity of Human Breast Cancer Cells Is Modulated by Differential Expression of Xanthine Oxidoreductase. J Cel Biochem (2008) 105(4):1008–26. 10.1002/jcb.21901 PMC2587521 18767115

[310] Chen D Forootan SS Gosney JR Forootan FS Ke Y. Increased Expression of Id1 and Id3 Promotes Tumorigenicity by Enhancing Angiogenesis and Suppressing Apoptosis in Small Cell Lung Cancer. Genes Cancer (2014) 5(5-6):212–25. 10.18632/genesandcancer.20 25061504 PMC4104762

[311] Sharma BK Kolhe R Black SM Keller JR Mivechi NF Satyanarayana A. Inhibitor of Differentiation 1 Transcription Factor Promotes Metabolic Reprogramming in Hepatocellular Carcinoma Cells. FASEB j (2016) 30(1):262–75. 10.1096/fj.15-277749 26330493 PMC4684534

[312] Singh B Berry JA Shoher A Ayers GD Wei C Lucci A. COX-2 Involvement in Breast Cancer Metastasis to Bone. Oncogene (2007) 26(26):3789–96. 10.1038/sj.onc.1210154 17213821

[313] Baldwin W McRae S Marek G Wymer D Pannu V Baylis C Hyperuricemia as a Mediator of the Proinflammatory Endocrine Imbalance in the Adipose Tissue in a Murine Model of the Metabolic Syndrome. Diabetes (2011) 60(4):1258–69. 10.2337/db10-0916 21346177 PMC3064099

[314] Gandhi J Khera L Gaur N Paul C Kaul R. Role of Modulator of Inflammation Cyclooxygenase-2 in Gammaherpesvirus Mediated Tumorigenesis. Front Microbiol (2017) 8:538. 10.3389/fmicb.2017.00538 28400769 PMC5368278

[315] Hou Z. Falcone D. J. Subbaramaiah K. Dannenberg A. J.. Macrophages induce COX-2 expression in breast cancer cells: role of IL-1β autoamplification. Carcin (2011) 32(5):695–702. 10.1093/carcin/bgr027 PMC3086701 21310944

[316] Martínez-Reyes C. P. Manjarrez-Reyna A. N. Méndez-García L. A. Aguayo-Guerrero J. A. Aguirre-Sierra B. Villalobos-Molina R Uric Acid Has Direct Proinflammatory Effects on Human Macrophages by Increasing Proinflammatory Mediators and Bacterial Phagocytosis Probably via URAT1. Biomolecules (2020) 10:576. 10.3390/biom10040576 PMC7225983 32283759

[317] Lee HE Lee JY Yang G Kang HC Cho Y-Y Lee HS Inhibition of NLRP3 Inflammasome in Tumor Microenvironment Leads to Suppression of Metastatic Potential of Cancer Cells. Scientific Rep (2019) 9(1):12277. 10.1038/s41598-019-48794-x PMC6706417 31439870

[318] Bent R Moll L Grabbe S Bros M. Interleukin-1 Beta-A Friend or Foe in Malignancies? Ijms (2018) 19(8):2155. 10.3390/ijms19082155 PMC6121377 30042333

[319] El-Deeb MMK El-Sheredy HG Mohammed AF. The Possible Role of Interleukin (IL)-18 and Nitrous Oxide and Their Relation to Oxidative Stress in the Development and Progression of Breast Cancer. Asian Pac J Cancer Prev (2019) 20(9):2659–65. 10.31557/apjcp.2019.20.9.2659 31554361 PMC6976825

