# Supplementary material for: The Adipokine Component in the Molecular Regulation of Cancer Cell Survival, Proliferation and Metastasis
Source: Pathol Oncol Res. 2021 Sep 13;27:1609828. doi: 10.3389/pore.2021.1609828 (PMC8473628; doi:10.3389/pore.2021.1609828)
Supplement: Supplementary file 1 [file Table1.DOCX]

**Supplementary Material**

Table S1: The expression and role of obesity mediators in cancer

| **Marker** | **Pathways activated** | | **Over-expression**  **(Disease Type)** |
| --- | --- | --- | --- |
|  | **Cancer-supportive** | **Cancer-suppressing** |  |
| Leptin | Upregulation of  Jak/STAT3, p44/p42, Erk1/2, Akt [1-5], CycD1 [1], MAT1A, MAT1β [6], hTERT [7], cdx2 & barx1 [8], PLOD2 [9], Notch 1, Survivin [10], Hey2, Oct-4, CD133, CD24/44, ALDH [11], SERPINE 1, IL-6, MMP-2, SNAI 2, TWIST 1 [12, 13], mTORC, p70S6, COX-2, TGFβ, MCP-1, CINC1 [14], MMP13 [15], hsp27 [16], MLC1, VEGFC, CCND1 [17].  Downregulation of  Bad, TNFR1, caspase 6 [18], CCN5 [19] | Upregulation of MAPK (androgen insensitive cells) [20], SOCS3 [8, 21], p57/p21 [22]  Downregulation of Sirtuin1 [22] | Breast [23-25], Colorectal [17, 26], thyroid [27, 28], gastric [29, 30], Endometrial [31, 32], Ovarian [33, 34], Lung [35, 36], Pancreatic [15, 37, 38], Prostate [39-41], Skin [42], Renal [43] |
| Resistin | Upregulation of SNAIL, SLUG, ZEB1, TWIST1, Fibronectin, Vimentin [44-46], ICAM1, VCAM1, LC3, BECN1, LAMP1, LC3B-II, ALDH1, CD44 (cytosolic), Oct4, NANONG, Sox2 [46, 47], CXCR4 [48], hTERT [49], Caveolin-1, STAT3 [47], JNK, AMPK/mTOR/ULK1 signaling [50]  Downregulation of E-cadherin, Claudin-1 [44-46], SQSTM1 [50] |  | Breast [25, 47, 50-52], Colon/Colorectal [53], Oral squamous cell carcinoma [54], Lung [55], Ovarian [46], Renal [56], Ovarian [57], Gastric/Gastroesophageal [48, 58] |
| Pre-B-cell colony enhancing factor 1/ Visfatin/ nicotinamide  phosphorybosyl transferase (NAMPT) | Upregulation of PI3K, P38 MAPK, JAK/STAT, JNK and Notch1 [59-63] [64], Rho/ROCK [65], CCL2 [65], NFκB, IL6 [66], Snail, vimentin and β-catenin, phosphorylation of GSK3β [67], MRP-1 [68]  Downregulation of P53 dependent apoptosis [69] |  | Breast [25, 64], Endometrial [70], Hepatocellular [71], Colorectal [72, 73], Gastric [74], Oral squamous cell carcinoma [75], Renal [56], Urothelial [76] |
| Omentin |  | Upregulation of HNF4α [77],  Downregulation of MMP-1 via attenuating LTF [78], attenuates PI3K/Akt mediated upregulation of NF-κB signaling [77] | Prostate [79, 80], Lung [81], Pancreatic [82] |
| Adiponectin |  | Upregulation of LKB1 [83], SOCS3 [84, 85], p53, p21, p27, Bax [86-88].  Downregulation of mTORC1 [83, 89-91], β-catenin [92], STAT3 [84, 85], PI3k/Akt [93, 94], Erk1/2 [86, 95, 96], NF-κB [97], CycD1,c-myc, Bcl2 [86-88], SREBP-1c [88]. | Renal [43, 56], **^Ɨ^** Breast [98] |
| Angiopoietin | Ang1 Potentiates Cell-ECM; Cell-endothelium attachment by upregulating β1-integrin and CD44 [99, 100]. Ang1 Upregulates VEGF mediated angiogenesis via Erk1/3, Akt signaling [101].  Ang2 activates ILK, GSK3β and Akt signaling via α5β1 integrins [102] and promotes EMT via downregulating E-cadherins [103]  Ang2 downregulates Ang1/Tie2 signaling in ER+ dormant cancers via activating β1 integrin [104] | Ang1 upregulates cell-cell attachments via Tie-2/Ang1 signaling [105-107].  Angiopoietin-3 Downregulates Ang1 and VEGF mediated angiogenesis [101] | Breast [102, 108-111], Gastric [111-113], Colorectal [114], Hepatocellular [115], Cervical [116], Pancreatic [117], Endometrial [118], Lung [119, 120] |
| Angiopoietin like proteins | ANGLP-2 promotes metastasis via EMT [121, 122], upregulation of VEGF, Ang2 and hexokinase-2 [123]; p38 MAPK, MMP1, MMP9, MMP13, integrin α5β1 [124].  ANGLP-4 promotes metastasis by downregulating endothelial cell interactions [125, 126].  ANGLP-6 induces cell invasion via α6 integrin/E-cadherin signaling [127] |  | Lung [121, 128, 129], Prostate [130], Pancreatic [131], Gastric [130], Breast [132, 133], Cervical [134] |
| Chemerin/ Retinoic acid receptor responder protein 2 | Upregulation of vinculin [135], MMP2, MMP9 via PI3k/Akt and MAPK pathways [136], VEGF [137], p38MAPK, Erk1/2, MMP7 [138], p42/44 Erk [139], recruitment of immunosuppressive pDC [140, 141]. | Upregulation of cytosolic ERK1/2 [142, 143], ERG1 and cFOS [135], PTEN [144], chemoattractant effect on tumor suppressive M1 macrophages [145] and NK cells [146].  Downregulation of FOXO1 [147, 148] | Colorectal [149, 150], Gastric [151], Breast [152], Lung [153], Oesophageal [154], Oral squamous cell carcinoma [155], Non-small cell lung carcinoma [156], Ovarian [157], **^Ɨ^**Hepatocellular carcinoma [30] |
| Lipocalin 2 | Upregulation of MMP1, MMP3, MMP9, IL1β, IL-6, IL-8, ICAM-1,  CXC motif chemokines 2/MCP-1 [158-161] [162], fibronectin, vimentin [163], escape from caspase-independent cell death via upregulation of LCF complex [164] inactivation of BIM via upregulation of cytosolic iron levels [165].  Downregulation of E-cadherin [163] | Upregulation of caveolin-1, E-cadherin, thrombospondin-1 in Ras- transformed cells [166]  Downregulation of  VEGF, Ras/MAPK, Ras/PI3K in Ras-Transformed cells [166, 167] | Pancreatic [168, 169], Cholangiocarcinoma [162], Breast [163, 170, 171], Prostate [172, 173], ovarian [174], Endometrial [175, 176], Colon [177], Thyroid [178, 179], Cervical [180, 181], Hepatocellular [182],  Gastric [161] |

**^Ɨ^** indicates either a negative correlation between marker expression and cancer progression, or a retardation of tumor size because of the expression of the marker.

# References:

1. Saxena, N.K., et al., *Concomitant activation of the JAK/STAT, PI3K/AKT, and ERK signaling is involved in leptin-mediated promotion of invasion and migration of hepatocellular carcinoma cells.* Cancer research, 2007. **67**(6): p. 2497-2507.

2. Saxena, N.K., et al., *Leptin as a novel profibrogenic cytokine in hepatic stellate cells: mitogenesis and inhibition of apoptosis mediated by extracellular regulated kinase (Erk) and Akt phosphorylation.* FASEB journal : official publication of the Federation of American Societies for Experimental Biology, 2004. **18**(13): p. 1612-1614.

3. Saxena, N.K., et al., *leptin-induced growth stimulation of breast cancer cells involves recruitment of histone acetyltransferases and mediator complex to CYCLIN D1 promoter via activation of Stat3.* The Journal of biological chemistry, 2007. **282**(18): p. 13316-13325.

4. Higurashi, T., et al., *Conditional knockout of the leptin receptor in the colonic epithelium revealed the local effects of leptin receptor signaling in the progression of colonic tumors in mice.* Carcinogenesis, 2014. **35**(9): p. 2134-41.

5. Pan, W., et al., *Transcriptional and physiological roles for STAT proteins in leptin action.* Molecular Metabolism, 2019. **22**: p. 121-131.

6. Ramani, K., et al., *Leptin's mitogenic effect in human liver cancer cells requires induction of both methionine adenosyltransferase 2A and 2beta.* Hepatology (Baltimore, Md.), 2008. **47**(2): p. 521-531.

7. Stefanou, N., et al., *Leptin as a critical regulator of hepatocellular carcinoma development through modulation of human telomerase reverse transcriptase.* BMC cancer, 2010. **10**: p. 442-442.

8. Inagaki-Ohara, K., et al., *Enhancement of leptin receptor signaling by SOCS3 deficiency induces development of gastric tumors in mice.* Oncogene, 2014. **33**(1): p. 74-84.

9. He, J.-Y., et al., *Adipocyte-derived IL-6 and leptin promote breast Cancer metastasis via upregulation of Lysyl Hydroxylase-2 expression.* Cell communication and signaling : CCS, 2018. **16**(1): p. 100-100.

10. Knight, B.B., et al., *Survivin upregulation, dependent on leptin-EGFR-Notch1 axis, is essential for leptin-induced migration of breast carcinoma cells.* Endocrine-related cancer, 2011. **18**(4): p. 413-428.

11. Harbuzariu, A., et al., *Leptin-Notch signaling axis is involved in pancreatic cancer progression.* Oncotarget, 2017. **8**(5): p. 7740-7752.

12. Sabol, R.A., et al., *Leptin produced by obesity-altered adipose stem cells promotes metastasis but not tumorigenesis of triple-negative breast cancer in orthotopic xenograft and patient-derived xenograft models.* Breast cancer research : BCR, 2019. **21**(1): p. 67-67.

13. Strong, A.L., et al., *Leptin produced by obese adipose stromal/stem cells enhances proliferation and metastasis of estrogen receptor positive breast cancers.* Breast Cancer Research, 2015. **17**(1): p. 112.

14. Fazolini, N.P.B., et al., *Leptin activation of mTOR pathway in intestinal epithelial cell triggers lipid droplet formation, cytokine production and increased cell proliferation.* Cell cycle (Georgetown, Tex.), 2015. **14**(16): p. 2667-2676.

15. Mendonsa, A.M., et al., *Modulation of the leptin receptor mediates tumor growth and migration of pancreatic cancer cells.* PloS one, 2015. **10**(4): p. e0126686-e0126686.

16. Valle, A., et al., *Proteomic analysis of MCF-7 breast cancer cell line exposed to leptin.* Analytical cellular pathology (Amsterdam), 2011. **34**(3): p. 147-157.

17. Nowakowska-Zajdel, E., et al., *Cellular signal transduction pathways by leptin in colorectal cancer tissue: preliminary results.* ISRN endocrinology, 2011. **2011**: p. 575397-575397.

18. Ptak, A., E. Kolaczkowska, and E.L. Gregoraszczuk, *Leptin stimulation of cell cycle and inhibition of apoptosis gene and protein expression in OVCAR-3 ovarian cancer cells.* Endocrine, 2013. **43**(2): p. 394-403.

19. Haque, I., et al., *Leptin-induced ER-α-positive breast cancer cell viability and migration is mediated by suppressing CCN5-signaling via activating JAK/AKT/STAT-pathway.* BMC cancer, 2018. **18**(1): p. 99-99.

20. Deo, D.D., et al., *Differential effects of leptin on the invasive potential of androgen-dependent and -independent prostate carcinoma cells.* Journal of biomedicine & biotechnology, 2008. **2008**: p. 163902-163902.

21. Knobelspies, H., et al., *Mechanism of attenuation of leptin signaling under chronic ligand stimulation.* BMC biochemistry, 2010. **11**: p. 2-2.

22. Zhao, X., et al., *Leptin changes differentiation fate and induces senescence in chondrogenic progenitor cells.* Cell death & disease, 2016. **7**(4): p. e2188-e2188.

23. Rahmati-Yamchi, M., et al., *Plasma Leptin, hTERT Gene Expression, and Anthropometric Measures in Obese and Non-Obese Women with Breast Cancer.* Breast cancer : basic and clinical research, 2011. **5**: p. 27-35.

24. Wu, M.H., et al., *Circulating levels of leptin, adiposity and breast cancer risk.* British journal of cancer, 2009. **100**(4): p. 578-582.

25. Assiri, A.M.A., H.F.M. Kamel, and M.F.R. Hassanien, *Resistin, visfatin, adiponectin, and leptin: risk of breast cancer in pre- and postmenopausal saudi females and their possible diagnostic and predictive implications as novel biomarkers.* Disease markers, 2015. **2015**: p. 253519-253519.

26. Koda, M., et al., *Overexpression of the obesity hormone leptin in human colorectal cancer.* Journal of clinical pathology, 2007. **60**(8): p. 902-906.

27. Uddin, S., et al., *Role of leptin and its receptors in the pathogenesis of thyroid cancer.* International journal of clinical and experimental pathology, 2011. **4**(7): p. 637-643.

28. Marcello, M.A., et al., *Polymorphism in LEP and LEPR May Modify Leptin Levels and Represent Risk Factors for Thyroid Cancer.* International journal of endocrinology, 2015. **2015**: p. 173218-173218.

29. Ishikawa, M., J. Kitayama, and H. Nagawa, *Expression pattern of leptin and leptin receptor (OB-R) in human gastric cancer.* World journal of gastroenterology, 2006. **12**(34): p. 5517-5522.

30. Pan, Y., et al., *Leptin-LepRb Expressed in Gastric Cancer Patients and Related to Cancer-Related Depression.* BioMed research international, 2017. **2017**: p. 6482842-6482842.

31. Sharma, D., et al., *Leptin promotes the proliferative response and invasiveness in human endometrial cancer cells by activating multiple signal-transduction pathways.* Endocrine-related cancer, 2006. **13**(2): p. 629-640.

32. Ma, Y., et al., *Serum leptin, adiponectin and endometrial cancer risk in Chinese women.* Journal of gynecologic oncology, 2013. **24**(4): p. 336-341.

33. Uddin, S., et al., *Overexpression of leptin receptor predicts an unfavorable outcome in Middle Eastern ovarian cancer.* Molecular cancer, 2009. **8**: p. 74-74.

34. Kato, S., et al., *Leptin stimulates migration and invasion and maintains cancer stem-like properties in ovarian cancer cells: an explanation for poor outcomes in obese women.* Oncotarget, 2015. **6**(25): p. 21100-21119.

35. Tong, X., et al., *Serum and tissue leptin in lung cancer: A meta-analysis.* Oncotarget, 2017. **8**(12): p. 19699-19711.

36. Xu, M., et al., *Leptin induces epithelial-to-mesenchymal transition via activation of the ERK signaling pathway in lung cancer cells.* Oncology letters, 2018. **16**(4): p. 4782-4788.

37. Babic, A., et al., *Pancreatic Cancer Risk Associated with Prediagnostic Plasma Levels of Leptin and Leptin Receptor Genetic Polymorphisms.* Cancer research, 2016. **76**(24): p. 7160-7167.

38. Stolzenberg-Solomon, R.Z., et al., *Circulating Leptin and Risk of Pancreatic Cancer: A Pooled Analysis From 3 Cohorts.* American journal of epidemiology, 2015. **182**(3): p. 187-197.

39. Hu, M.-B., et al., *Genetic polymorphisms in leptin, adiponectin and their receptors affect risk and aggressiveness of prostate cancer: evidence from a meta-analysis and pooled-review.* Oncotarget, 2016. **7**(49): p. 81049-81061.

40. Alshaker, H., et al., *Leptin signalling, obesity and prostate cancer: molecular and clinical perspective on the old dilemma.* Oncotarget, 2015. **6**(34): p. 35556-35563.

41. Hoda, M.R., et al., *The adipocyte-derived hormone leptin has proliferative actions on androgen-resistant prostate cancer cells linking obesity to advanced stages of prostate cancer.* Journal of oncology, 2012. **2012**: p. 280386-280386.

42. Farag, A.G.A., N.F. Elnaidany, and M.M.S. El-Dien, *Immunohistochemical Expression of Leptin in Non Melanoma Skin Cancer.* Journal of clinical and diagnostic research : JCDR, 2016. **10**(8): p. WC08-WC12.

43. Liao, L.M., et al., *Serum leptin and adiponectin levels and risk of renal cell carcinoma.* Obesity (Silver Spring, Md.), 2013. **21**(7): p. 1478-1485.

44. Weber, D., et al., *SAT-335 Resistin Induces Epithelial to Mesenchymal Transition (EMT) in Breast Cancer Cells through Activation of AXL Tyrosine Kinase Receptor.* Journal of the Endocrine Society, 2019. **3**(Suppl 1): p. SAT-335.

45. Avtanski, D., et al., *Resistin induces breast cancer cells epithelial to mesenchymal transition (EMT) and stemness through both adenylyl cyclase-associated protein 1 (CAP1)-dependent and CAP1-independent mechanisms.* Cytokine, 2019. **120**: p. 155-164.

46. Qiu, L., et al., *Novel oncogenic and chemoresistance-inducing functions of resistin in ovarian cancer cells require miRNAs-mediated induction of epithelial-to-mesenchymal transition.* Scientific reports, 2018. **8**(1): p. 12522-12522.

47. Deshmukh, S.K., et al., *Resistin potentiates chemoresistance and stemness of breast cancer cells: Implications for racially disparate therapeutic outcomes.* Cancer letters, 2017. **396**: p. 21-29.

48. Hsieh, Y.-Y., et al., *Resistin-induced stromal cell-derived factor-1 expression through Toll-like receptor 4 and activation of p38 MAPK/ NFκB signaling pathway in gastric cancer cells.* Journal of biomedical science, 2014. **21**(1): p. 59-59.

49. Mohammadi, M., et al., *RESISTIN EFFECT ON TELOMERASE GENE EXPRESSION IN GASTRIC CANCER CELL LINE AGS.* Acta endocrinologica (Bucharest, Romania : 2005), 2016. **12**(2): p. 145-149.

50. Liu, Z., et al., *Resistin confers resistance to doxorubicin-induced apoptosis in human breast cancer cells through autophagy induction.* American journal of cancer research, 2017. **7**(3): p. 574-583.

51. Avtanski, D., et al., *SAT-334 Proinflammatory Cytokines Modulate Resistin Expression in Breast Cancer Cells.* Journal of the Endocrine Society, 2019. **3**(Suppl 1): p. SAT-334.

52. Zeidan, B., et al., *Increased circulating resistin levels in early-onset breast cancer patients of normal body mass index correlate with lymph node negative involvement and longer disease free survival: a multi-center POSH cohort serum proteomics study.* Breast cancer research : BCR, 2018. **20**(1): p. 19-19.

53. Sălăgeanu, A., et al., *Serum levels of adipokines resistin and leptin in patients with colon cancer.* Journal of medicine and life, 2010. **3**(4): p. 416-420.

54. Yang, W.-H., et al., *Association of Resistin Gene Polymorphisms with Oral Squamous Cell Carcinoma Progression and Development.* BioMed research international, 2018. **2018**: p. 9531315-9531315.

55. Demiray, G., et al., *Effects of Serum Leptin and Resistin Levels on Cancer Cachexia in Patients With Advanced-Stage Non-Small Cell Lung Cancer.* Clinical Medicine Insights. Oncology, 2017. **11**: p. 1179554917690144-1179554917690144.

56. Zhang, H.-P., et al., *Association of leptin, visfatin, apelin, resistin and adiponectin with clear cell renal cell carcinoma.* Oncology letters, 2017. **13**(1): p. 463-468.

57. Pang, L., et al., *Resistin promotes the expression of vascular endothelial growth factor in ovary carcinoma cells.* International journal of molecular sciences, 2013. **14**(5): p. 9751-9766.

58. Diakowska, D., et al., *Serum levels of resistin, adiponectin, and apelin in gastroesophageal cancer patients.* Disease markers, 2014. **2014**: p. 619649-619649.

59. Adya, R., et al., *Visfatin induces human endothelial VEGF and MMP-2/9 production via MAPK and PI3K/Akt signalling pathways: novel insights into visfatin-induced angiogenesis.* Cardiovasc Res, 2008. **78**(2): p. 356-65.

60. Wang, P., et al., *Perivascular adipose tissue-derived visfatin is a vascular smooth muscle cell growth factor: role of nicotinamide mononucleotide.* Cardiovasc Res, 2009. **81**(2): p. 370-80.

61. Kim, S.R., et al., *Visfatin promotes angiogenesis by activation of extracellular signal-regulated kinase 1/2.* Biochem Biophys Res Commun, 2007. **357**(1): p. 150-6.

62. Liu, P., et al., *Regulation of inflammatory cytokine expression in pulmonary epithelial cells by pre-B-cell colony-enhancing factor via a nonenzymatic and AP-1-dependent mechanism.* J Biol Chem, 2009. **284**(40): p. 27344-51.

63. Nowell, M.A., et al., *Regulation of pre-B cell colony-enhancing factor by STAT-3-dependent interleukin-6 trans-signaling: implications in the pathogenesis of rheumatoid arthritis.* Arthritis Rheum, 2006. **54**(7): p. 2084-95.

64. Park, H.-J., et al., *Visfatin promotes cell and tumor growth by upregulating Notch1 in breast cancer.* Oncotarget, 2014. **5**(13): p. 5087-5099.

65. Liu, T., et al., *Visfatin Mediates SCLC Cells Migration across Brain Endothelial Cells through Upregulation of CCL2.* Int J Mol Sci, 2015. **16**(5): p. 11439-51.

66. Wang, G.J., et al., *Visfatin triggers the in vitro migration of osteosarcoma cells via activation of NF-kappaB/IL-6 signals.* Eur J Pharmacol, 2016. **791**: p. 322-330.

67. Yang, J., et al., *Visfatin is involved in promotion of colorectal carcinoma malignancy through an inducing EMT mechanism.* Oncotarget, 2016. **7**(22): p. 32306-17.

68. Cao, Z., et al., *Visfatin mediates doxorubicin resistance in human non-small-cell lung cancer via Akt-mediated up-regulation of ABCC1.* Cell proliferation, 2017. **50**(5): p. e12366.

69. Zhao, H., et al., *The NAMPT/E2F2/SIRT1 axis promotes proliferation and inhibits p53-dependent apoptosis in human melanoma cells.* Biochem Biophys Res Commun, 2017. **493**(1): p. 77-84.

70. Cymbaluk-Płoska, A., et al., *Circulating Serum Level of Visfatin in Patients with Endometrial Cancer.* BioMed research international, 2018. **2018**: p. 8576179-8576179.

71. Sun, Y., et al., *Elevated serum visfatin levels are associated with poor prognosis of hepatocellular carcinoma.* Oncotarget, 2017. **8**(14): p. 23427-23435.

72. Neubauer, K., et al., *Nampt/PBEF/visfatin upregulation in colorectal tumors, mirrored in normal tissue and whole blood of colorectal cancer patients, is associated with metastasis, hypoxia, IL1β, and anemia.* BioMed research international, 2015. **2015**: p. 523930-523930.

73. Yang, J., et al., *Visfatin is involved in promotion of colorectal carcinoma malignancy through an inducing EMT mechanism.* Oncotarget, 2016. **7**(22): p. 32306-32317.

74. Gorgian Mohammadi, M., et al., *Adipocyte Derived Hormones Gene Expression, Resistin and Visfatin, in AGS Gastric Cancer Cell Line.* Iranian journal of cancer prevention, 2013. **6**(3): p. 165-169.

75. Yu-Duan, T., et al., *Elevated plasma level of visfatin/pre-b cell colony-enhancing factor in male oral squamous cell carcinoma patients.* Medicina oral, patologia oral y cirugia bucal, 2013. **18**(2): p. e180-e186.

76. Ke, H.-L., et al., *High visfatin expression predicts poor prognosis of upper tract urothelial carcinoma patients.* American journal of cancer research, 2015. **5**(8): p. 2447-2454.

77. Li, D., et al., *Intelectin 1 suppresses tumor progression and is associated with improved survival in gastric cancer.* Oncotarget, 2015. **6**(18): p. 16168-16182.

78. Kay-Pong, Y., L.A.-Y. Chi, and C.M. Samuel, *Interactions of Omentin and Lactotransferrin in the Progression of Metastatic Ovarian Cancer.* The FASEB Journal, 2019. **33**(1_supplement): p. 704.5-704.5.

79. Fryczkowski, M., et al., *Circulating Levels of Omentin, Leptin, VEGF, and HGF and Their Clinical Relevance with PSA Marker in Prostate Cancer.* Disease markers, 2018. **2018**: p. 3852401-3852401.

80. Zhou, L., et al., *Altered circulating levels of adipokine omentin-1 in patients with prostate cancer.* OncoTargets and therapy, 2019. **12**: p. 3313-3319.

81. Ansari, M.H.K., et al., *Association of circulating omentin-1 level with lung cancer in smokers.* Medical journal of the Islamic Republic of Iran, 2018. **32**: p. 133-133.

82. Karabulut, S., et al., *Clinical significance of serum omentin-1 levels in patients with pancreatic adenocarcinoma.* BBA clinical, 2016. **6**: p. 138-142.

83. Taliaferro-Smith, L., et al., *LKB1 is required for adiponectin-mediated modulation of AMPK-S6K axis and inhibition of migration and invasion of breast cancer cells.* Oncogene, 2009. **28**(29): p. 2621-2633.

84. Miyazaki, T., et al., *Adiponectin activates c-Jun NH2-terminal kinase and inhibits signal transducer and activator of transcription 3.* Biochem Biophys Res Commun, 2005. **333**(1): p. 79-87.

85. Partida-Pérez, M., et al., *Association of LEP and ADIPOQ common variants with colorectal cancer in Mexican patients.* Cancer Biomark, 2010. **7**(3): p. 117-21.

86. Dieudonne, M.N., et al., *Adiponectin mediates antiproliferative and apoptotic responses in human MCF7 breast cancer cells.* Biochem Biophys Res Commun, 2006. **345**(1): p. 271-9.

87. Luo, Z., et al., *AMPK, the metabolic syndrome and cancer.* Trends in Pharmacological Sciences, 2005. **26**(2): p. 69-76.

88. Kim, A.Y., et al., *Adiponectin Represses Colon Cancer Cell Proliferation via AdipoR1- and -R2-Mediated AMPK Activation.* Molecular Endocrinology, 2010. **24**(7): p. 1441-1452.

89. Zakikhani, M., et al., *The effects of adiponectin and metformin on prostate and colon neoplasia involve activation of AMP-activated protein kinase.* Cancer Prev Res (Phila), 2008. **1**(5): p. 369-75.

90. Sugiyama, M., et al., *Adiponectin inhibits colorectal cancer cell growth through the AMPK/mTOR pathway.* International journal of oncology, 2009. **34**(2): p. 339-344.

91. Saxena, N.K., et al., *Adiponectin modulates C-jun N-terminal kinase and mammalian target of rapamycin and inhibits hepatocellular carcinoma.* Gastroenterology, 2010. **139**(5): p. 1762-73, 1773.e1-5.

92. Wang, Y., et al., *Adiponectin Modulates the Glycogen Synthase Kinase-3β/β-Catenin Signaling Pathway and Attenuates Mammary Tumorigenesis of MDA-MB-231 Cells in Nude Mice.* Cancer Research, 2006. **66**(23): p. 11462-11470.

93. Habeeb, B.S., J. Kitayama, and H. Nagawa, *Adiponectin supports cell survival in glucose deprivation through enhancement of autophagic response in colorectal cancer cells.* Cancer Sci, 2011. **102**(5): p. 999-1006.

94. Li, G., et al., *Mechanisms Underlying the Anti-Proliferative Actions of Adiponectin in Human Breast Cancer Cells, MCF7–Dependency on the cAMP/Protein Kinase-A Pathway.* Nutrition and Cancer, 2011. **63**(1): p. 80-88.

95. Körner, A., et al., *Total and high-molecular-weight adiponectin in breast cancer: in vitro and in vivo studies.* J Clin Endocrinol Metab, 2007. **92**(3): p. 1041-8.

96. Cong, L., et al., *Human adiponectin inhibits cell growth and induces apoptosis in human endometrial carcinoma cells, HEC-1-A and RL95 2.* Endocr Relat Cancer, 2007. **14**(3): p. 713-20.

97. Fenton, J.I., et al., *Adiponectin blocks multiple signaling cascades associated with leptin-induced cell proliferation in Apc Min/+ colon epithelial cells.* Int J Cancer, 2008. **122**(11): p. 2437-45.

98. Mantzoros, C., et al., *Adiponectin and breast cancer risk.* J Clin Endocrinol Metab, 2004. **89**(3): p. 1102-7.

99. Ou, X.-L., et al., *Effects of angiopoietin-1 on attachment and metastasis of human gastric cancer cell line BGC-823.* World journal of gastroenterology, 2009. **15**(43): p. 5432-5441.

100. Holopainen, T., et al., *Angiopoietin-1 overexpression modulates vascular endothelium to facilitate tumor cell dissemination and metastasis establishment.* Cancer Res, 2009. **69**(11): p. 4656-64.

101. Xu, Y., Y.J. Liu, and Q. Yu, *Angiopoietin-3 inhibits pulmonary metastasis by inhibiting tumor angiogenesis.* Cancer Res, 2004. **64**(17): p. 6119-26.

102. Imanishi, Y., et al., *Angiopoietin-2 stimulates breast cancer metastasis through the alpha(5)beta(1) integrin-mediated pathway.* Cancer research, 2007. **67**(9): p. 4254-4263.

103. Li, C., et al., *Overexpression of angiopoietin 2 promotes the formation of oral squamous cell carcinoma by increasing epithelial–mesenchymal transition-induced angiogenesis.* Cancer Gene Therapy, 2016. **23**(9): p. 295-302.

104. Han, H.H., et al., *Angiopoietin-2 promotes ER+ breast cancer cell survival in bone marrow niche.* Endocr Relat Cancer, 2016. **23**(8): p. 609-23.

105. Kitajima, D., et al., *Tie2 Regulates Tumor Metastasis of Oral Squamous Cell Carcinomas.* Journal of Cancer, 2016. **7**(5): p. 600-607.

106. Kitajima, D., et al., *Evidence for critical role of Tie2/Ang1 interaction in metastatic oral cancer.* Oncology letters, 2018. **15**(5): p. 7237-7242.

107. Michael, I.P., et al., *Angiopoietin-1 deficiency increases tumor metastasis in mice.* BMC cancer, 2017. **17**(1): p. 539-539.

108. Tian, S., et al., *Stabilization of breast cancer xenograft tumour neovasculature by angiopoietin-1.* British journal of cancer, 2002. **86**(4): p. 645-651.

109. Hayes, A.J., et al., *Expression and function of angiopoietin-1 in breast cancer.* British journal of cancer, 2000. **83**(9): p. 1154-1160.

110. Bohn, K.A., et al., *Inhibition of VEGF and Angiopoietin-2 to Reduce Brain Metastases of Breast Cancer Burden.* Frontiers in pharmacology, 2017. **8**: p. 193-193.

111. Tang, S., et al., *miR-218 suppresses gastric cancer cell proliferation and invasion via regulation of angiopoietin-2.* Experimental and therapeutic medicine, 2016. **12**(6): p. 3837-3842.

112. Wang, J., et al., *Antisense angiopoietin-1 inhibits tumorigenesis and angiogenesis of gastric cancer.* World journal of gastroenterology, 2006. **12**(15): p. 2450-2454.

113. Sun, X.-D., et al., *Expression and significance of angiopoietin-2 in gastric cancer.* World journal of gastroenterology, 2004. **10**(9): p. 1382-1385.

114. Hong, S., et al., *Expressions and Clinical Significances of Angiopoietin-1, Angiopoietin-2, and Tie-2 Receptor in Patients With Colorectal Cancer.* Annals of coloproctology, 2017. **33**(1): p. 9-15.

115. Faillaci, F., et al., *Liver Angiopoietin-2 Is a Key Predictor of De Novo or Recurrent Hepatocellular Cancer After Hepatitis C Virus Direct-Acting Antivirals.* Hepatology (Baltimore, Md.), 2018. **68**(3): p. 1010-1024.

116. Yang, P., et al., *The ratio of serum Angiopoietin-1 to Angiopoietin-2 in patients with cervical cancer is a valuable diagnostic and prognostic biomarker.* PeerJ, 2017. **5**: p. e3387-e3387.

117. Wang, H., et al., *MiR-145 functions as a tumor suppressor via regulating angiopoietin-2 in pancreatic cancer cells.* Cancer cell international, 2016. **16**(1): p. 65-65.

118. Xu, X., et al., *Combined silencing of VEGF-A and angiopoietin-2, a more effective way to inhibit the Ishikawa endometrial cancer cell line.* OncoTargets and therapy, 2019. **12**: p. 1215-1223.

119. Xu, Y., et al., *The role of serum angiopoietin-2 levels in progression and prognosis of lung cancer: A meta-analysis.* Medicine, 2017. **96**(37): p. e8063-e8063.

120. Zhou, L., et al., *Plasma angiopoietin-2 is persistently elevated after non-small cell lung cancer surgery and stimulates angiogenesis in vitro.* Medicine, 2016. **95**(32): p. e4493-e4493.

121. Sasaki, H., et al., *Angiopoietin-like protein ANGPTL2 gene expression is correlated with lymph node metastasis in lung cancer.* Oncology letters, 2012. **4**(6): p. 1325-1328.

122. Aoi, J., et al., *Angiopoietin-like protein 2 is an important facilitator of inflammatory carcinogenesis and metastasis.* Cancer Res, 2011. **71**(24): p. 7502-12.

123. Wang, X., et al., *Angiopoietin-like protein 2 is an important facilitator of tumor proliferation, metastasis, angiogenesis and glycolysis in osteosarcoma.* Am J Transl Res, 2019. **11**(10): p. 6341-6355.

124. Odagiri, H., et al., *The secreted protein ANGPTL2 promotes metastasis of osteosarcoma cells through integrin alpha5beta1, p38 MAPK, and matrix metalloproteinases.* Sci Signal, 2014. **7**(309): p. ra7.

125. Zhang, H., et al., *HIF-1-dependent expression of angiopoietin-like 4 and L1CAM mediates vascular metastasis of hypoxic breast cancer cells to the lungs.* Oncogene, 2012. **31**(14): p. 1757-70.

126. Padua, D., et al., *TGFbeta primes breast tumors for lung metastasis seeding through angiopoietin-like 4.* Cell, 2008. **133**(1): p. 66-77.

127. Marchiò, S., et al., *A complex of α6 integrin and E-cadherin drives liver metastasis of colorectal cancer cells through hepatic angiopoietin-like 6.* EMBO molecular medicine, 2012. **4**(11): p. 1156-1175.

128. Kuo, T.-C., et al., *Angiopoietin-like protein 1 suppresses SLUG to inhibit cancer cell motility.* The Journal of clinical investigation, 2013. **123**(3): p. 1082-1095.

129. Wei, X., et al., *Angiopoietin-like protein 2 facilitates non-small cell lung cancer progression by promoting the polarization of M2 tumor-associated macrophages.* American journal of cancer research, 2017. **7**(11): p. 2220-2233.

130. Toiyama, Y., et al., *Serum angiopoietin-like protein 2 as a potential biomarker for diagnosis, early recurrence and prognosis in gastric cancer patients.* Carcinogenesis, 2015. **36**(12): p. 1474-1483.

131. Yoshinaga, T., et al., *Angiopoietin-like Protein 2 is a Useful Biomarker for Pancreatic Cancer that is Associated with Type 2 Diabetes Mellitus and Inflammation.* Journal of Cancer, 2018. **9**(24): p. 4736-4741.

132. Zhang, H., et al., *HIF-1-dependent expression of angiopoietin-like 4 and L1CAM mediates vascular metastasis of hypoxic breast cancer cells to the lungs.* Oncogene, 2012. **31**(14): p. 1757-1770.

133. Kolb, R., et al., *Obesity-associated inflammation promotes angiogenesis and breast cancer via angiopoietin-like 4.* Oncogene, 2019. **38**(13): p. 2351-2363.

134. Nie, D., et al., *Up-regulated of Angiopoietin-Like Protein 4 Predicts Poor Prognosis in Cervical Cancer.* Journal of Cancer, 2019. **10**(8): p. 1896-1901.

135. Rourke, J.L., H.J. Dranse, and C.J. Sinal, *CMKLR1 and GPR1 mediate chemerin signaling through the RhoA/ROCK pathway.* Mol Cell Endocrinol, 2015. **417**: p. 36-51.

136. Kaur, J., et al., *Identification of chemerin receptor (ChemR23) in human endothelial cells: chemerin-induced endothelial angiogenesis.* Biochem Biophys Res Commun, 2010. **391**(4): p. 1762-8.

137. Bozaoglu, K., et al., *Chemerin, a novel adipokine in the regulation of angiogenesis.* The Journal of clinical endocrinology and metabolism, 2010. **95**(5): p. 2476-2485.

138. Wang, C., et al., *Increased serum chemerin level promotes cellular invasiveness in gastric cancer: a clinical and experimental study.* Peptides, 2014. **51**: p. 131-8.

139. Nakamura, N., et al., *Chemerin promotes angiogenesis in vivo.* Physiol Rep, 2018. **6**(24): p. e13962.

140. Skrzeczynska-Moncznik, J., et al., *Potential role of chemerin in recruitment of plasmacytoid dendritic cells to diseased skin.* Biochemical and biophysical research communications, 2009. **380**: p. 323-7.

141. Zabel, B.A., A.M. Silverio, and E.C. Butcher, *Chemokine-like receptor 1 expression and chemerin-directed chemotaxis distinguish plasmacytoid from myeloid dendritic cells in human blood.* J Immunol, 2005. **174**(1): p. 244-51.

142. De Henau, O., et al., *Signaling Properties of Chemerin Receptors CMKLR1, GPR1 and CCRL2.* PloS one, 2016. **11**(10): p. e0164179-e0164179.

143. DeFea, K.A., et al., *beta-arrestin-dependent endocytosis of proteinase-activated receptor 2 is required for intracellular targeting of activated ERK1/2.* J Cell Biol, 2000. **148**(6): p. 1267-81.

144. Li, J.J., et al., *Chemerin suppresses hepatocellular carcinoma metastasis through CMKLR1-PTEN-Akt axis.* Br J Cancer, 2018. **118**(10): p. 1337-1348.

145. Herová, M., et al., *ChemR23, the receptor for chemerin and resolvin E1, is expressed and functional on M1 but not on M2 macrophages.* J Immunol, 2015. **194**(5): p. 2330-7.

146. Pachynski, R.K., et al., *The chemoattractant chemerin suppresses melanoma by recruiting natural killer cell antitumor defenses.* The Journal of experimental medicine, 2012. **209**(8): p. 1427-1435.

147. Lakshmikanthan, V., et al., *Identification of βArrestin2 as a corepressor of androgen receptor signaling in prostate cancer.* Proceedings of the National Academy of Sciences, 2009. **106**(23): p. 9379-9384.

148. Duan, X., et al., *β-Arrestin2 Contributes to Cell Viability and Proliferation via the Down-Regulation of FOXO1 in Castration-Resistant Prostate Cancer.* J Cell Physiol, 2015. **230**(10): p. 2371-81.

149. Eichelmann, F., et al., *Association of Chemerin Plasma Concentration With Risk of Colorectal Cancer.* JAMA network open, 2019. **2**(3): p. e190896-e190896.

150. Alkady, M.M., P.L. Abdel-Messeih, and N.M. Nosseir, *Assessment of Serum Levels of the Adipocytokine Chemerin in Colorectal Cancer Patients.* Journal of medical biochemistry, 2018. **37**(3): p. 313-319.

151. Kumar, J.D., et al., *Chemerin acts via CMKLR1 and GPR1 to stimulate migration and invasion of gastric cancer cells: putative role of decreased TIMP-1 and TIMP-2.* Oncotarget, 2019. **10**(2): p. 98-112.

152. El-Sagheer, G., et al., *Expression of chemerin correlates with a poor prognosis in female breast cancer patients.* Breast cancer (Dove Medical Press), 2018. **10**: p. 169-176.

153. Xu, C.-H., et al., *Prognostic significance of serum chemerin levels in patients with non-small cell lung cancer.* Oncotarget, 2017. **8**(14): p. 22483-22489.

154. Kumar, J.D., et al., *The role of chemerin and ChemR23 in stimulating the invasion of squamous oesophageal cancer cells.* British journal of cancer, 2016. **114**(10): p. 1152-1159.

155. Cao, D., et al., *Metallopanstimulin-1 (MPS-1) mediates the promotion effect of leptin on colorectal cancer through activation of JNK/c-Jun signaling pathway.* Cell death & disease, 2019. **10**(9): p. 655-655.

156. Sotiropoulos, G.P., et al., *Chemerin as a biomarker at the intersection of inflammation, chemotaxis, coagulation, fibrinolysis and metabolism in resectable non-small cell lung cancer.* Lung Cancer, 2018. **125**: p. 291-299.

157. Schutyser, E., et al., *Identification of biologically active chemokine isoforms from ascitic fluid and elevated levels of CCL18/pulmonary and activation-regulated chemokine in ovarian carcinoma.* J Biol Chem, 2002. **277**(27): p. 24584-93.

158. Gomez-Chou, S.B., et al., *Lipocalin-2 Promotes Pancreatic Ductal Adenocarcinoma by Regulating Inflammation in the Tumor Microenvironment.* Cancer research, 2017. **77**(10): p. 2647-2660.

159. Tschesche, H., et al., *The human neutrophil lipocalin supports the allosteric activation of matrix metalloproteinases.* Eur J Biochem, 2001. **268**(7): p. 1918-28.

160. Yan, L., et al., *The high molecular weight urinary matrix metalloproteinase (MMP) activity is a complex of gelatinase B/MMP-9 and neutrophil gelatinase-associated lipocalin (NGAL). Modulation of MMP-9 activity by NGAL.* J Biol Chem, 2001. **276**(40): p. 37258-65.

161. Kubben, F.J.G.M., et al., *Clinical evidence for a protective role of lipocalin-2 against MMP-9 autodegradation and the impact for gastric cancer.* European Journal of Cancer, 2007. **43**(12): p. 1869-1876.

162. Nuntagowat, C., K. Leelawat, and R. Tohtong, *NGAL knockdown by siRNA in human cholangiocarcinoma cells suppressed invasion by reducing NGAL/MMP-9 complex formation.* Clinical & Experimental Metastasis, 2010. **27**(5): p. 295-305.

163. Yang, J., et al., *Lipocalin 2 promotes breast cancer progression.* Proceedings of the National Academy of Sciences of the United States of America, 2009. **106**(10): p. 3913-3918.

164. Schafer, Z.T., et al., *Antioxidant and oncogene rescue of metabolic defects caused by loss of matrix attachment.* Nature, 2009. **461**(7260): p. 109-113.

165. Devireddy, L.R., et al., *A Cell-Surface Receptor for Lipocalin 24p3 Selectively Mediates Apoptosis and Iron Uptake.* Cell, 2005. **123**(7): p. 1293-1305.

166. Venkatesha, S., et al., *Lipocalin 2 Antagonizes the Proangiogenic Action of Ras in Transformed Cells.* Molecular Cancer Research, 2006. **4**(11): p. 821-829.

167. Hanai, J., et al., *Lipocalin 2 diminishes invasiveness and metastasis of Ras-transformed cells.* J Biol Chem, 2005. **280**(14): p. 13641-7.

168. Ochi, N., et al., *Can CXC-Chemokines and Lipocalin 2 in Exocrine Pancreatic Secretions Distinguish Chronic Pancreatitis From Pancreatic Cancer?* Gastrointestinal Cancer Research : GCR, 2010(Suppl 1): p. S30-S30.

169. Kaur, S., et al., *MUC4-mediated regulation of acute phase protein lipocalin 2 through HER2/AKT/NF-κB signaling in pancreatic cancer.* Clinical cancer research : an official journal of the American Association for Cancer Research, 2014. **20**(3): p. 688-700.

170. Shi, H., et al., *Lipocalin 2 promotes lung metastasis of murine breast cancer cells.* Journal of experimental & clinical cancer research : CR, 2008. **27**(1): p. 83-83.

171. Drew, B.G., et al., *Estrogen receptor (ER)α-regulated lipocalin 2 expression in adipose tissue links obesity with breast cancer progression.* The Journal of biological chemistry, 2015. **290**(9): p. 5566-5581.

172. Mahadevan, N.R., et al., *ER stress drives Lipocalin 2 upregulation in prostate cancer cells in an NF-κB-dependent manner.* BMC cancer, 2011. **11**: p. 229-229.

173. Ding, G., et al., *Lipocalin 2 over-expression facilitates progress of castration-resistant prostate cancer via improving androgen receptor transcriptional activity.* Oncotarget, 2016. **7**(39): p. 64309-64317.

174. Cho, H. and J.-H. Kim, *Lipocalin2 expressions correlate significantly with tumor differentiation in epithelial ovarian cancer.* The journal of histochemistry and cytochemistry : official journal of the Histochemistry Society, 2009. **57**(5): p. 513-521.

175. Cymbaluk-Płoska, A., et al., *The role of lipocalin-2 serum levels in the diagnostics of endometrial cancer.* Cancer biomarkers : section A of Disease markers, 2019. **24**(3): p. 315-324.

176. Mannelqvist, M., et al., *Lipocalin 2 expression is associated with aggressive features of endometrial cancer.* BMC cancer, 2012. **12**: p. 169-169.

177. Maier, H.T., et al., *Up-regulation of neutrophil gelatinase-associated lipocalin in colorectal cancer predicts poor patient survival.* World J Surg, 2014. **38**(8): p. 2160-7.

178. Celestino, R., et al., *CRABP1, C1QL1 and LCN2 are biomarkers of differentiated thyroid carcinoma, and predict extrathyroidal extension.* BMC cancer, 2018. **18**(1): p. 68-68.

179. Iannetti, A., et al., *The neutrophil gelatinase-associated lipocalin (NGAL), a NF-kappaB-regulated gene, is a survival factor for thyroid neoplastic cells.* Proc Natl Acad Sci U S A, 2008. **105**(37): p. 14058-63.

180. Vitkauskaitė, A., et al., *Associations among Serum Lipocalin-2 Concentration, Human Papilloma Virus and Clinical Stage of Cervical Cancer.* Medicina (Kaunas, Lithuania), 2019. **55**(6): p. 229.

181. Chung, I.H., et al., *Overexpression of lipocalin 2 in human cervical cancer enhances tumor invasion.* Oncotarget, 2016. **7**(10): p. 11113-11126.

182. Chung, I.H., et al., *Thyroid hormone-mediated regulation of lipocalin 2 through the Met/FAK pathway in liver cancer.* Oncotarget, 2015. **6**(17): p. 15050-15064.
